# Bridging Targeted (Zeno MRM-HR) and Untargeted (SWATH) LC–HRMS in a Single Run for Sensitive Exposomics

**DOI:** 10.1021/acs.analchem.4c01630

**Published:** 2024-07-26

**Authors:** Vinicius Verri Hernandes, Benedikt Warth

**Affiliations:** † Department of Food Chemistry and Toxicology, Faculty of Chemistry, University of Vienna, 1090 Vienna, Austria; ‡ Exposome Austria, Research Infrastructure and National EIRENE Node, 1090 Vienna, Austria

## Abstract

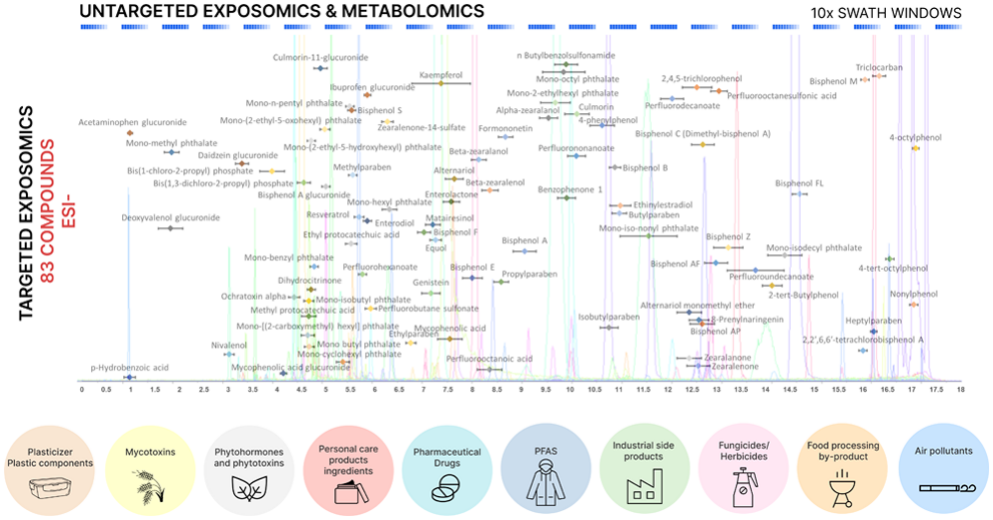

Traditionally, chemical exposure has been assessed by low-resolution mass spectrometry via targeted approaches due to the typically extremely low concentration of such compounds in biological samples. Nevertheless, untargeted approaches are now becoming a promising tool for a broader investigation of the exposome, covering additional compounds, their biotransformation products, and possible metabolic alterations (metabolomics). However, despite broad compound coverage, untargeted metabolomics still underperforms in ultratrace biomonitoring analysis. To overcome these analytical limitations, we present the development of the first combined targeted/untargeted LC–MS method, merging MRM-HR and SWATH experiments in one analytical run, making use of Zeno technology for improved sensitivity. Multiple reaction monitoring transitions were optimized for 135 highly diverse toxicants including mycotoxins, plasticizers, PFAS, personal care products ingredients, and industrial side products as well as potentially beneficial xenobiotics such as phytohormones. As a proof of concept, standard reference materials of human plasma (SRM 1950) and serum (SRM 1958) were analyzed with both Zeno MRM-HR + SWATH and SWATH-only methodologies. Results demonstrated a significant increase in sensitivity represented by the detection of lower concentration levels in spiked SRM materials (mean value: 2.2 and 3 times lower concentrations for SRMs 1950 and 1958, respectively). Overall, the detection frequency was increased by 68% (19 to 32 positive detections) in the MRM-HR + SWATH mode compared to the SWATH-only. This work presents a promising avenue for addressing the outstanding key challenge in the small-molecule omics field: finding a balance between high sensitivity and broad chemical coverage. It was demonstrated for exposomic applications but might be transferred to lipidomics and metabolomics workflows.

## Introduction

Humans are exposed to a myriad of chemicals throughout their lifespan, including industrial pollutants, food nutrients and contaminants, and synthetic products in cosmetics, among many others. With that, there is an increasing interest in the measurement of known and novel chemicals as well as their possible effects on human health, especially under the recently coined idea of exposomics. First conceptualized by Wild,^1^ the exposome has been defined by Miller and Jones^2^ as “the cumulative measure of environmental influences and associated biological responses throughout the lifespan, including exposures from the environment, diet, behavior, and endogenous processes”.

When designing any small-molecule “omics” study such as exposomics or metabolomics, one of the first decisions is typically the choice between targeted and untargeted approaches. The first normally relies on the use of low-resolution instruments (such as quadrupoles or ion traps) and aims at providing quantitative data with increased sensitivity when compared with high-resolution instruments, usually relying on time-of-flight or Orbitrap analyzers. These methods normally include tens to hundreds of compounds and are commonly used for confirming previous hypotheses. In exposomics, for example, these methods are used for large biomonitoring studies due to their capability to provide quantitative information for trace-level chemicals,^3^ ultimately providing policy markers with information for the science-to-policy interface regarding chemical exposure.^4^ Untargeted approaches, on the other hand, have the advantage of detecting, in principle, any compound at a given concentration that can be ionized. For that reason, untargeted methodologies have gained momentum in the last years specially under the strategies termed suspect screening analysis (SSA) and nontargeted analysis (NTA), which enables the screening of hundreds to thousands of (potentially novel and relevant) chemicals.^4^ In addition, untargeted data acquisition approaches can be used for the assessment of the “effects” part of the exposure, i.e., the possible impact on the levels of endogenous metabolites due to a particular exposure pattern via metabolomics strategies.^5^

When acquiring such type of data using LC–MS or LC-HRMS setups, different acquisition modes can be selected. For targeted analysis, multiple reaction monitoring (MRM) is the common choice, and it is based on the detection of usually two transitions (precursor-product ion pairs) for each analyte, allowing for high sensitivity and specificity. For untargeted data, data-dependent acquisition (DDA) and data-independent acquisition (DIA) are the most commonly used approaches. The first relies on the selection of the n most intense *m*/*z* detected at MS^1^ level to further selection and fragmentation (MS^2^ acquisition), normally leading to good quality MS^2^ spectra of a few of the detected features.^6^ DIA, on the other hand, has the capability to fragment virtually all detected signals, later performing spectra deconvolution in order to assign each fragment to the most likely precursor. As a result, the number of MS^2^ spectra acquired is higher, leading to better possibilities of identification, especially for features with lower intensity that would not be selected by DDA experiments. As a downside, deconvolution algorithms are not 100% accurate, and MS^2^ spectra become either more polluted (with missassigned fragments) or less informative (with missing fragments assigned to a different precursor), which can ultimately lead to inadequate spectral matching. SWATH (sequential window acquisition of all theoretical fragments) is one type of DIA acquisition in which sequential ranges of precursor isolation windows are selected and fragmented, allowing for the acquisition of MS^2^ data for virtually all ions detected at the MS^1^ level.^7,8^

Cajka and Fiehn (2016)^9^ reviewed targeted and untargeted metabolomics data to evaluate the possibility of having one method that would provide broad chemical coverage in conjunction with reliable quantitative data using high-resolution mass spectrometry. As future directions, the authors highlight the efforts needed toward the combination of targeted and untargeted metabolomics approaches via untargeted MS^1^ data acquisition and high-resolution MRM transitions for targeting multiple metabolites. More recently, Bird et al. (2023)^10^ have introduced a new workflow based on Orbitrap and/or TOF systems (SQUAD, simultaneous quantitation and discovery analysis) aiming at combining both targeted and untargeted with the main objective of finding the equilibrium between untargeted and targeted approaches in a single experiment.

This paper describes, a targeted/untargeted, single-injection LC–MS method for integrated exposomics and metabolomics, combining the advantages of both approaches, i.e., high sensitivity (targeted) and broad coverage and retrospective data analysis (untargeted). The use of the recently introduced Zeno technology, a linear ion trap stage prior to the TOF analyzer that allows for increased duty cycles of up to 90%,^11^ resulting in significant increases in sensitivity. In addition to presenting the results obtained for different xenobiotics in the targeted mode (135 compounds) as well as the results for the analysis of standard reference materials (SRM 1950 and SRM 1958), we also describe the method development process that is expected to support other researchers working in the field of small-molecule omics.

## Experimental Section

### Chemicals and Sample Preparation

Multicomponent standard solutions containing 135 compounds (Table S1) were prepared at six different concentrations levels. The selection of compounds was intended to represent a highly diverse and commonly observed chemical exposure panel, considering factors such as chemical classes, origin of exposure, exposure route, among others. In addition, the selection of compounds was based on a previous work published by our group for a multiclass targeted methodology.^12^ Concentrations were compound-dependent and are described in detail in Table S1.

Standard reference materials (SRM 1950 and 1958 from NIST) were purchased from NIST and stored at −80 °C. In total, nine replicates of each SRMs were prepared following the protocol previously described by Jamnik et al. (2022).^13^ For extraction, 30 μL of both SRMs were mixed with 120 μL of extraction solution (ACN/MeOH 50:50, v/v) in Eppendorf tubes and sonicated for 10 min on ice. The extraction solution contained labeled IS (Table S2) for accounting for the total variation (sample preparation + instrumental analysis). A 2 h protein precipitation step was performed at −20 °C afterward, followed by centrifugation for 10 min at 18,000*g* at 4 °C. The supernatant (120 μL) was transferred to a new Eppendorf tube and dried under vacuum at 4 °C overnight. From the nine replicates, three were reconstituted using 120 μL of ACN/H_2_O 10:90, v/v. The additional six replicates were used to build a matrix-matched calibration curve with the same solvent composition and spiked at the same levels as the standard solutions in solvent. Samples were vortexed for 10 min after reconstitution and centrifuged at 18,000*g* and 4 °C. Finally, 100 μL of the supernatant was transferred to an amber glass vial for data acquisition. The sample preparation protocol was chosen to maximize chemical coverage.^14^

### LC-HRMS Instrumentation and Parameters

An Infinity II 1290 LC system (Agilent) coupled to a SCIEX ZenoTOF 7600 system with a Turbo V source was used. Chromatographic separation was based on the same method described by Jamnik^13^ et al. (2022). In summary, a 20 min reversed-phase gradient was employed on an HSS T3 column (Waters), with a flow rate of 0.4 mL/min and an injection volume of 5 μL.

LC–MS data was acquired for the Zeno MRM-HR + SWATH methodology as well as for a SWATH-only method. Ion source parameters were standardized across both methodologies as follows: GS1-50 psi; GS2-50 psi; curtain gas-35 psi; CAD gas-9; and temperature −550 °C. In both cases, MS^1^ data were acquired from *m*/*z* 100 to 1000, with declustering potential (DP) at 80 V (−80 V for negative mode) and collision energy (CE) at 10 V (−10 V for negative mode). Spray voltage was set at 5500 V for the positive ionization mode (ESI^+^) and −4500 V for the negative ionization mode (ESI^–^).

For the developed Zeno MRM-HR + SWATH data, accumulation time was set at 0.05 s for individual MRM transitions and at 0.02 s for each SWATH window (*n* = 10). SWATH windows were optimized individually for each SRM using the SWATH windows optimizer provided by SCIEX. Both SWATH-only and MRM-HR + SWATH methods had the exact same parameter for SWATH acquisition, including windows range, DP (80 or −80 V), and CE, as described in detail in Table S3. For the MRM experiment, the Start/Stop option was selected, with the TOF Start Mass at *m*/*z* 50 and the TOF Stop Mass set as compound-dependent by adding 2 *m*/*z* units to precursor *m*/*z*. Retention time windows, declustering potentials, and collision energies were adjusted for each compound (see the Results and Discussion section), and Zeno Pulsing was activated at a threshold of 8000 cps.

To allow for a point of comparison, the cycle time for the SWATH-only method was adjusted to a value similar to the maximum scan time for the MRM-HR + SWATH method. To evaluate possible differences in increased accumulation time for MS^1^ or SWATH windows, two different approaches were taken. For the Zeno MRM-HR + SWATH in the negative mode, accumulation time for MS^1^ was increased from 0.05 to 0.15 s (for both SRM 1958 and SRM 1950). For the positive mode, on the other hand, MS^1^ accumulation time was kept the same as for the MRM-HR + SWATH method (0.05 s), and instead, accumulation times for SWATH-only windows were increased such that the total cycle time would compare to the maximum cycle time for MRM-HR + SWATH. For instance, the maximum scan time for the Zeno MRM-HR + SWATH method was 0.435 s, while the SWATH-only total cycle time was 0.25 s (0.05 s for MS1 and 0.02 s for each of the 10 SWATH window). Therefore, at the maximum number of overlapping MRM windows, the total cycle time would be 0.685 s. Based on that, the SWATH-only method for positive mode was set as total scan time of 0.643 by keeping MS1 acquisition at 0.05 s and increasing SWATH windows accumulation time from 0.02 to 0.05 s. For a direct comparison, key method parameters are summarized in Table 1.

**Table 1:** Parameters Employed across the Different MS Methods^a^

	NEG		POS	
	SWATH-only	MRM-HR + SWATH	SWATH-only	MRM-HR + SWATH
MS^1^ accumulation time (s)	0.15	0.05	0.05	0.05
total number of SWATH windows	10	10	10	10
SWATH window accumulation time (s)	0.06	0.02	0.05	0.02
MRM transitions	-	83	-	52
accumulation time MRM transitions (s)	-	0.05	-	0.05
cycle time (s)	0.843	0.763	0.643	0.685

a For SWATH-only, cycle time represents the total scan time (constant across the whole chromatographic run). For MRM-HR + SWATH, cycle time represents the maximum scan time since cycle time changes along the chromatographic run due to its dependency on the number of overlapping MRM transitions.

Finally, the analytical batch was set by alternating between the MRM-HR + SWATH and SWATH-only data acquisition for the same sample. That approach was taken in order to minimize batch effects in comparison of the results for the same sample between the two different methodologies.

### Compound Optimization

Two key parameters, namely, CE and DP (a voltage applied to the orifice to minimize solvent clusters and help on desolvation of ions) were optimized by either direct infusion (DP) or using an LC–MS method (CE). For DP, submixes of all compounds at either 10 or 100 ng/mL (depending on ionization efficiency) of all standards were constantly injected in the source using a syringe pump set at 5 μL/min. Automatic DP optimization was performed in SCIEX OS in the range from 10 to 300 V. For CE optimization, MS^2^ spectra were first acquired using DDA with the inclusion list for each of the submixes at in the range of 100–1000 ng/mL. MS^2^ spectra were acquired using the CE spread (CES) at 35 ± 15 V. This allows for a combined (and representative) spectra of three collision energies: 20, 35, and 50 V. Next, the five most intense fragments (or fragments known to work best in our targeted approach using low-resolution instruments^13^) were selected for further optimization. Finally, an MRM-HR method was created with all selected transitions, and submixes were injected multiple times using the above-described LC–MS method with one specific CE for each injection, in the range from 15 to 60 V, with steps of 5 V (total of nine injections per submix). Peak areas were used to select the best fragment and CE (Table S1).

### Data Analysis and Quality Check

Targeted data analysis (MRM-HR) was performed using SCIEX OS version 3.0.0.3339. Regardless of the MS mode of acquisition, extracted ion chromatograms for SWATH-only (MS^1^ level) and MRM + SWATH (MS^2^ level) were extracted using the default window of 0.02 Da. Chromatograms were integrated by the AutoPeak method and smoothed using a noise filter algorithm with the "Low" option. Concentrations were calculated for all compounds detected in nonspiked SRMs by standard addition method.

For quality control, peak areas of the internal standards in the ESI^–^ mode were inspected in the MS^1^ mode for both SWATH-only and MRM-HR + SWATH. From the labeled IS employed, none was found to be ionized in ESI^+^ so relative standard deviation values (RSD) were only estimated to be similar to those in ESI^–^. Peak areas for the MRM + SWATH mode have presented higher RSD (Table S4), which may be explained by the lower values for peak areas. These may additionally be related to the fact that accumulation time at MS^1^ for SWATH-only in ESI^–^ mode was set three times longer than that for MRM-HR + SWATH, therefore providing better sensitivity. Nevertheless, it is also important to highlight that this variation is likely to be considerably smaller for the MS^2^ data in MRM-HR + SWATH due to the increased peak areas provided by the Zeno pulsing technique, which are, in fact, the data presented and evaluated along this work. In addition, labeled IS were added to the extraction solution and, therefore, account for the sum of the variances of both extraction process and analytical run. For PFOS, for example, recovery at a very low concentration was reported^13^ as 56 ± 25%, representing 50% of variance only related to sample preparation. As the average RSD was significantly below this value, we may infer that the analytical variance was minimal. Total variance for recovery was also reported^13^ for genistein and estradiol at 30 and 14%, respectively, while RSD in our analysis were at 24 and 23% for MRM-HR + SWATH data.

Finally, MS^2^ matching for both exogenous (xenobiotics) and endogenous (metabolites) compounds was performed in MS-DIAL^15^ (version 5.2.240218.2) against Mass Bank of North America (MoNA) and MS-DIAL spectral libraries. For ESI^+^, LC–MS/MS Positive Mode (MoNA - 99,260 spectra) and ESI(+)–MS/MS from authentic standards (MS-DIAL, 324,191 spectra) were used. For ESI^–^, LC–MS/MS Negative Mode (MoNA - 47,058 spectra) and ESI(−)-MS/MS from authentic standards (MS-DIAL-44,669 spectra) were employed. A spectral match score of 70% was used as a threshold, with no limitation for minimum dot and reverse dot-product scores (since data were manually curated afterward). All method parameters from MS-Dial (from peak detection to identification) are described in Table S5.

## Results and Discussion

### Balancing Accumulation Time, RT Window, and Total Cycle Time

Considering the significant number of compounds included in each method, i.e., 83 in the ESI^–^ mode and 52 in ESI^+^, it is essential to optimize the total cycle time needed to acquire 1 × MS^1^ + 10 × SWATH windows +1 × MS^1^ + 83 × MRM transitions. This is related to the fact that, as a rule of thumb, a chromatographic peak should have at least between six and ten data points^16^ in order to achieve satisfactory Gaussian shape and reproducibility. Consequently, the above-mentioned acquisition cycle must be repeated at least six times along the width of every chromatographic peak for proper quantification. Therefore, the accumulation time for each of the mentioned experiments must be fine-tuned. The parameters found to be the most important are the accumulation time and the retention time window of each MRM transition, both due to the high number of overlapping MRM transitions.

**Figure 1:**
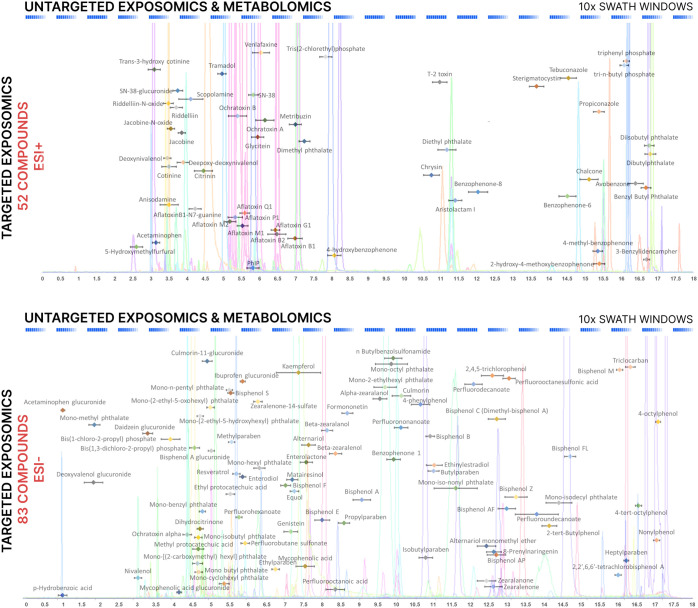
Overview of extracted ion chromatograms, retention times, and detection windows in both ESI^–^ and ESI^+^ modes. A total of 135 compounds from a variety of chemical classes were included in the MRM-HR experiments, with 83 compounds in the ESI^–^ mode and 52 in the ESI^+^ mode. In addition to the targeted quantification of the toxicant panel, the acquisition of SWATH data allows for the untargeted screening of additional xenobiotics, their biotransformation products, as well as endogenous metabolites (no peaks depicted).

To optimize RT windows, MS^1^ level data were acquired for the mixture of analytical standards at the highest concentration level. From these data, we retrieved the peak width for each compound and opted for an RT window that would be defined as 1.5 * total peak width. For example, methylparaben (RT = 5.55 min) was detected with a baseline peak width of ∼6.6 s, and therefore, the RT window was set at 10 s (±5 s). Manual fine-adjustment was also performed afterward, especially for compounds which are prone to suffer from relatively larger RT shifts (such as phthalates and glucuronide conjugates). Final RT windows are described for each compound in Table S1. Figure 1 presents the final and optimized methods with extracted ion chromatograms and RT and RT windows for each compound of the target panel.

### Benchmarking with SRM

#### Evaluation of Detection Levels (Sensitivity)

**Figure 2:**
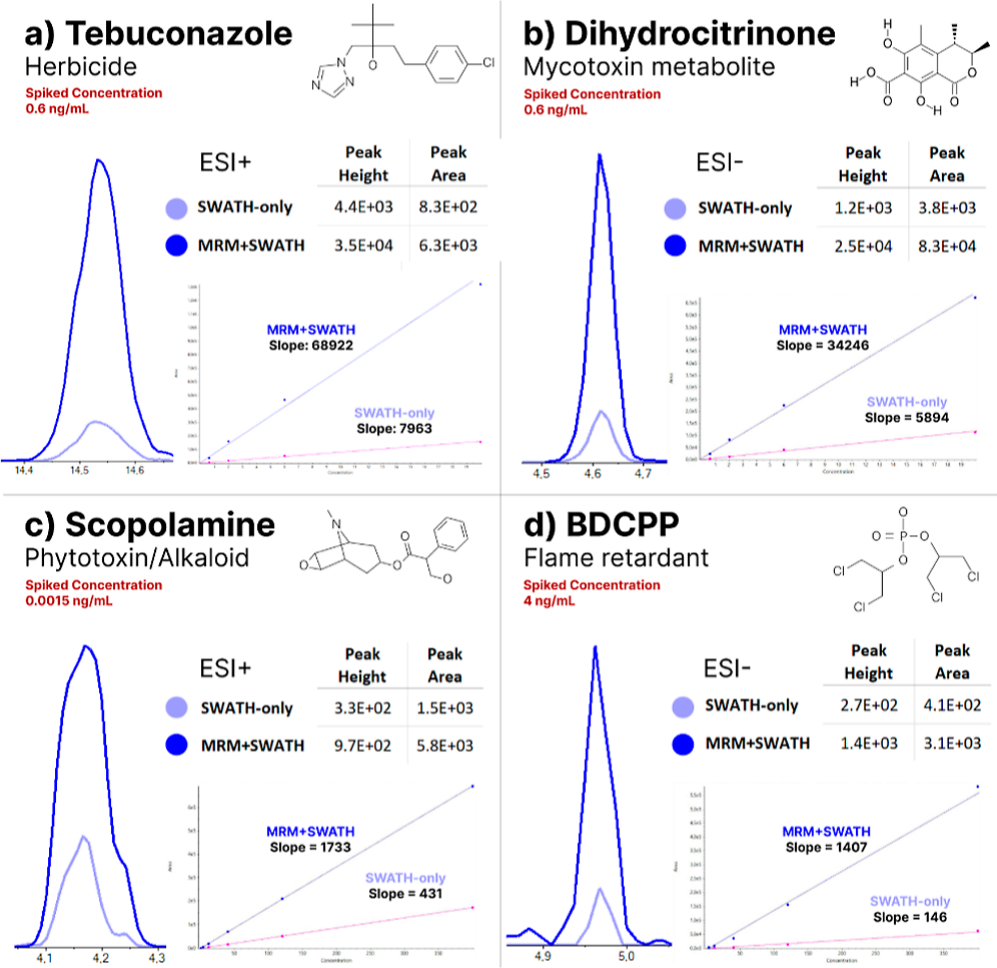
Extracted ion chromatograms for the lowest detected levels of four illustrative compounds in (a) SRM 1950 – ESI^+^; (b) SRM 1950 – ESI^–^; (c) SRM 1958 – ESI^+^; and (d) SRM 1958 ESI^–^. Despite not being able to detect each compound at a lower concentration level, MRM-HR + SWATH approach is able to provide larger peak areas and, consequently, better sensitivity.

One of the main parameters that allows for a sensitivity comparison between two methods is the signal-to-noise ratio (S/N). Nevertheless, comparing S/N between different levels of MS data, i.e., MS^1^ for SWATH-only and MS^2^ for MRM-HR + SWATH would not provide a meaningful comparison. One of the main reasons for this is the expected increase in specificity when dealing with MS^2^ data, resulting frequently in a flat (nonexistent) baseline which, consequently, makes noise estimation impractical. In addition, S/N estimation algorithms included into SCIEX OS (namely, standard deviation and peak to peak) require a noise region to be defined. Nevertheless, due to the use of very narrow RT windows for MRM experiments, selecting this noise region becomes unrealistic and, as a consequence, estimated S/N values turn out to be not representative. For these reasons, we have opted to report, for each analyte, the lowest concentration level that could be detected in the spiked matrix (and not detected in the nonspiked matrix) as a sensitivity comparison basis. Two criteria were considered to define if a peak was detected or not: a minimum intensity threshold of 100 counts and the visual inspection of the baseline (when possible) to estimate S/N. For a few borderline cases, we opted for a more conservative approach to avoid overestimation of the method’s performance. This approach was taken for all compounds that were not detected in the nonspiked matrix (for compounds detected in nonspiked matrix, see next section). For SWATH-only data, the MS^1^ level is reported since compounds could be detected at lower levels when compared to the MS^2^ level (which may be explained for the nonspecific CE chosen for each compound). For MRM-HR + SWATH data, on the other hand, MS^2^ level data showed larger values for peak areas when compared to MS^1^ data, as was also expected due to the optimized CE and the use of the Zeno trapping. In summary, mean detected concentrations in MRM-HR + SWATH were 2.1 and 2.3 times lower for SRM 1950 in ESI^–^ and ESI^+^ modes, respectively. For SRM 1958, a four times higher mean detected concentration was found for ESI^–^, while ESI^+^ presented a twofold increase. Even for compounds in which the lowest detected level was the same between MRM-HR + SWATH and SWATH-only, there were prevalent larger peak areas and calibration curve slopes for the first, as depicted in Figure 2 for four exemplary compounds. The complete description of the lowest detected levels for all compounds using both methods is reported in Table S6.

#### Detection and Quantification of Xenobiotics

Different xenobiotics were detected in both SRM 1958 and 1950 and are summarized in Table 2. Concentrations were calculated based on the calibration curves built for MRM-HR + SWATH and SWATH-only methods (Tables S7 and S8). Concentration values were not significantly different in almost all cases when considering raw *p*-values from a paired *t*-test (performed in MetaboAnalyst using the individual concentrations for each replicate). Exceptions were monobenzyl phthalate and *trans*-3-hydroxy-cotinine for SRM 1958 and nonylphenol and PFOS for SRM 1950. Acetaminophen was found to be different between methods for both the SRM 1950 and SRM 1958. The results showcase the higher capability of compound detection. From the total of 17 compounds detected in SRM 1958, roughly 41% (7) were detected in MRM-HR + SWATH mode only, covering different classes (bisphenols, PFAS, phthalates, and herbicides/pesticides such as metribuzin). Similarly, 44% of the compounds detected in SRM 1950 (7 out of 16) were found only with the use of the combined method.

**Table 2:**
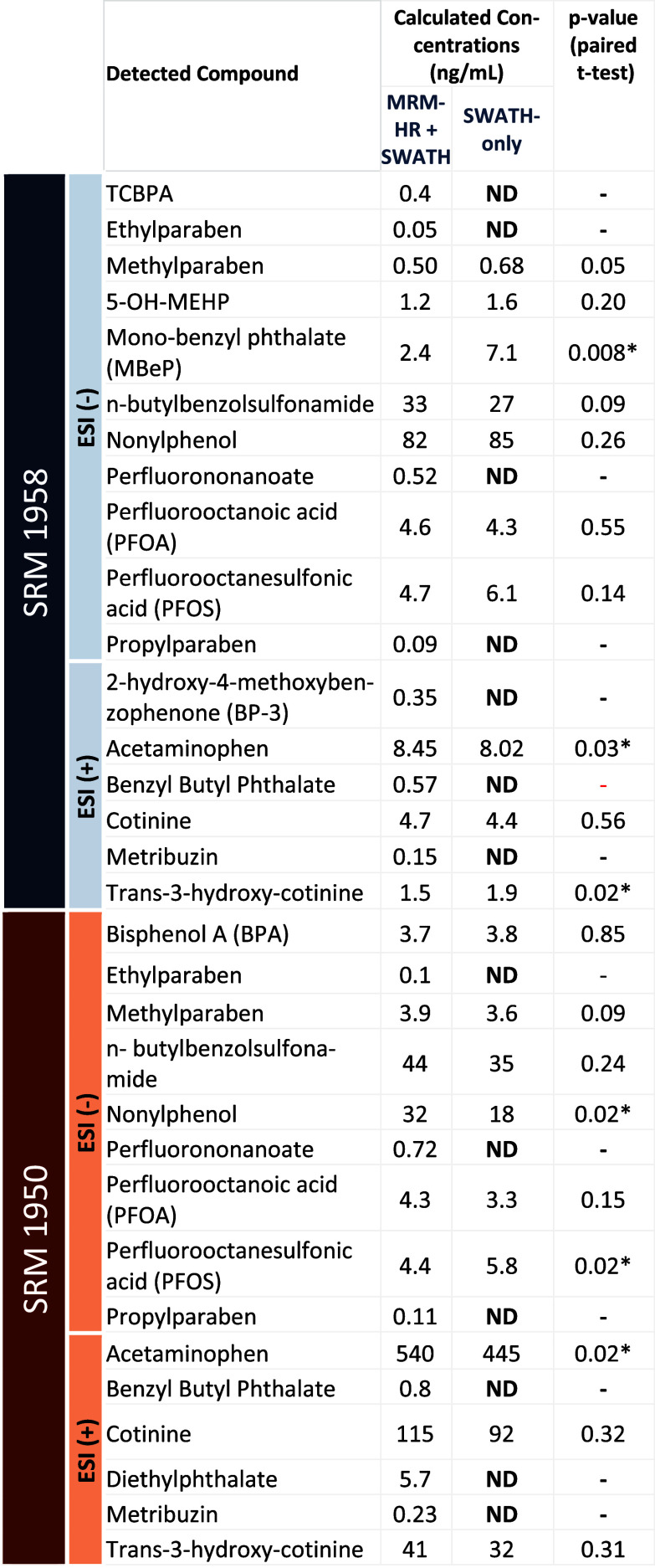
Detected Compounds with Calculated Average Concentrations for MRM-HR + SWATH and SWATH-Only (*n* = 3)^a^

a A paired *t*-test on individual concentrations for each replicate was performed to evaluate possible differences between both methods. *p*-values <0.05 are labelled with an *.

#### Annotation of Endogenous Metabolites for Integrated Metabolome Analysis

For evaluating the performance of each method for compound annotation, the nonspiked samples of SRM 1950 and 1958 were investigated, independently, for both MRM-HR + SWATH and SWATH-only using MS-DIAL (two sets of triplicate nonspiked samples, see Material and Methods, Data analysis, and quality control section for detailed information). As an initial assessment, the number of features obtained using the same processing method was evaluated. As observed in Table 3, MRM-HR + SWATH data retrieved a larger number of features for all experiments. When the total number of MS^2^ matchings retrieved from the libraries was compared, SWATH-only data initially seemed to outperform the newly developed MRM-HR + SWATH method.

**Table 3:**
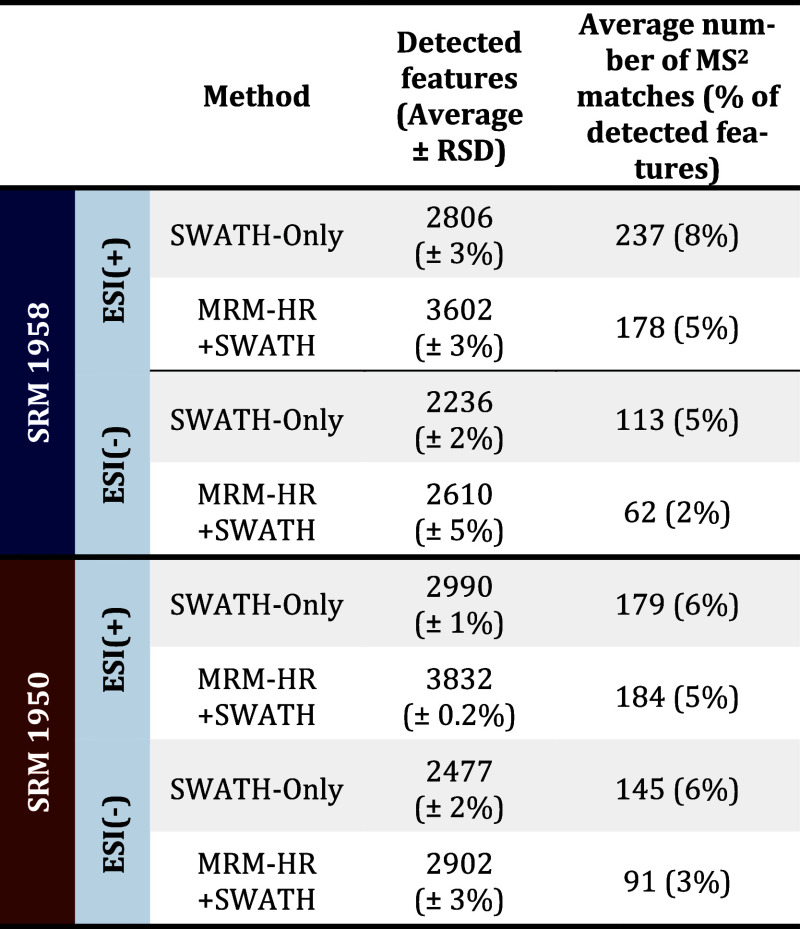
Performance Variables for MS^2^ Matching for Endogenous Compounds against Open-Source MS^2^ Libraries^a^

a Results indicate a superior matching frequency for SWATH-only data. Nevertheless, manual data curation evidences a similar matching rate/score between both methods, confirming the capability of the newly developed methodology to assess different parts of the metabolome.

**Figure 3:**
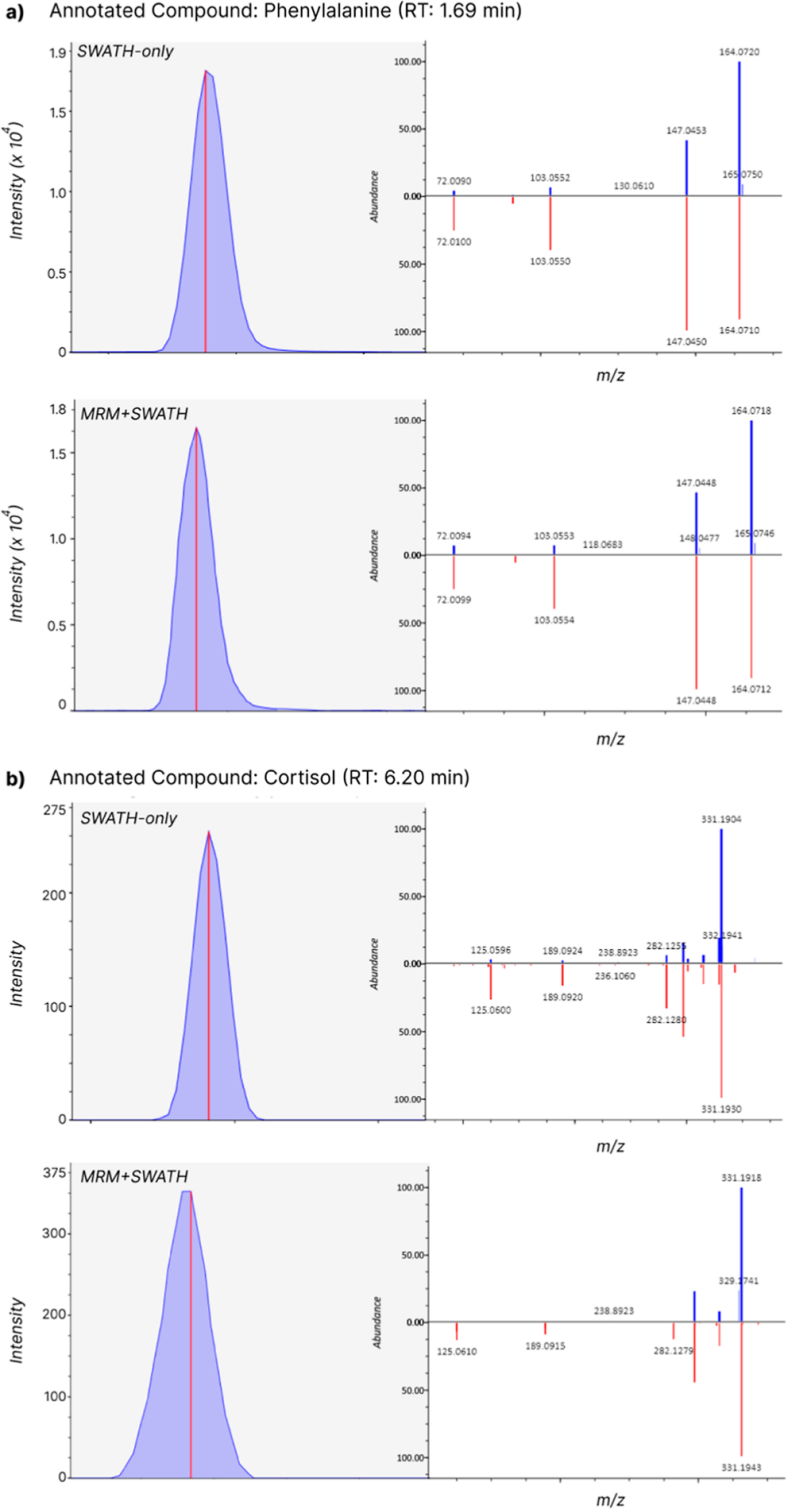
Exemplary MS^2^ spectra matching in ESI^–^ for (a) phenylalanine and (b) cortisol as highly and low correlated spectra (respectively) for SWATH-only and MRM-HR + SWATH methods. For cortisol, three fragments are not present in the deconvoluted spectrum of MRM-HR + SWATH (*m*/z 282, 189, and 125).

After manual curation of the data and removal of false positives, it was observed that the annotation of compounds was, in fact, of high similarity between both methods. More specifically, all compounds that could be reasonably annotated (minimum of two matching ions and mass error below 5 ppm for parent compound) in SWATH-only mode had the same suggested compound in MRM-HR + SWATH, with highly comparable matching scores (dot product, reverse dot product, and total score^15^). Mirror plots of annotated compounds for SRM 1950 in both ESI^+^ and ESI^–^ including matching scores are described in detail in Tables S9 and S10, along with all mirror plots for both methodologies (Figures S1–S45). Since a large overlap was observed for the compounds annotated between SRM 1958 and SRM 1950 in both ionization modes, we opted to not include mirror plots for the first as a way to avoid repetitive description of the data. Despite the clear similarity in MS^2^ matching performance, it is important to highlight that the SWATH-only method indeed outperformed MRM-HR + SWATH data in the quality of a few spectral matches, more prominently for low abundance compounds (peak height <1000 counts). Figure 3 presents two illustrative cases of spectral matches. The first (phenylalanine, Figure 3a) showcases an optimal MS^2^ match with the library for both SWATH-only and MRM-HR + SWATH, outlining the high MS^2^ spectral similarity between both methods. Figure 3b, on the other hand, intends to exemplify a case for which, despite the good matching score for both methods, spectra similarity is lower across both methods with missing fragments for the deconvoluted MS^2^ spectrum from MRM-HR + SWATH. This may be explained by the lower accumulation time for both MS^1^ and MS^2^ data, possibly hindering the deconvolution process (i.e., matching fragments with precursor ions). The same trend can be observed, for instance, for citric acid (Figure S1) and tyrosine (Figure S5) as highly similar spectral matches between both methods (with peak heights all above 1500) as well as for 4-nitrophenol (Figure S21) and glycocholic acid (Figure S22) as less reliable matches in MRM-HR + SWATH due to missing fragments in the deconvoluted spectra and with peak heights for both compounds at around 300 counts.

## Limitations

Despite the clear method capability toward a more sensitive and comprehensive analysis combining metabolomics and exposomics as well as targeted and untargeted in a single run, the method is not out of its limitations. Depending on the number of compounds, optimizing chromatographic separation, CE, DP, and total cycle time is time-intensive, especially for a relatively large number of compounds as described herein. We also acknowledge the lack of labeled internal standards for absolute quantification. Although adding such compounds would lead to a more accurate calculation of the concentrations, it should be considered that additional MRM transitions would be required, further elongating total cycle time. Regarding the untargeted data, there is still the need to evaluate the influence (if any) of different SWATH accumulation times in terms of signal intensity, quality of deconvoluted spectra, and, ultimately, in the total number of annotated compounds. Moreover, total accumulation times (and, consequently, cycle times) may still be fine-tuned. One example is the MRM accumulation time, which can be set as compound-dependent rather than as a unique value, further optimizing total cycle time based on compound-dependent parameters such as peak width as well as the total number of overlapping transitions in a given chromatogprahic time.

## Conclusions and Future Perspectives

A combination of targeted and untargeted approaches for single-injection LC-HRMS exposomics/metabolomics is described. The method makes use of the Zeno trap in the MRM-HR mode, recently developed with the aim to correct for duty cycle issues related to TOF instruments, allowing for increased sensitivity. The results highlight the method’s capabilities and its potential to detect xenobiotics present at low concentrations that otherwise would not be detected (or would be poorly detected) using a SWATH-only methodology. The method also allows for future customized developments, not only in terms of which compounds to include in the targeted list but also regarding the choice of a semi or absolute quantitation. In the field of metabolomics, for instance, this approach could be employed to detect poorly ionized and/or low concentration compounds, e.g., endogenous hormones. Additionally, applications in which a limited amount of sample is available (such as for serum samples of premature infants) or sample amount is limited by the sample collection technique (such as dried blood spots or volumetric microsampling devices) may also benefit, producing targeted and untargeted data acquisition in a single injection. In conclusion, we demonstrated that this type of data acquisition shows great potential for future application in any field of small-molecules “omics” and can be an interesting alternative for the long-time discussed compromise between sensitivity and coverage.

## Data Availability

The raw data files will be provided via the MetaboLights data repository (MTBLS10565).

## Supporting Information Available

Detailed information on the method including compound list, optimized parameters, concentration ranges, calibration curves, and lowest detected level for SRMs 1950 and 1958 (XLSX)
Mirror plots and matching scores for annotated compounds in the ESI- mode and ESI+ mode (PDF)
