# Supplementary material for: Bridging Targeted (Zeno MRM-HR) and Untargeted (SWATH) LC–HRMS in a Single Run for Sensitive Exposomics
Source: Anal Chem. 2024 Jul 26;96(31):12710–7. doi: 10.1021/acs.analchem.4c01630 (PMC11307248; doi:10.1021/acs.analchem.4c01630)

Supporting Information

Bridging targeted (Zeno MRM-HR) and untargeted (SWATH) LC-HRMS in a single run for sensitive exposomics.

Vinicius Verri Hernandes†,#, Benedikt Warth†,#

†Department of Food Chemistry and Toxicology, Faculty of Chemistry, University of Vienna, 1090 Vienna, Austria

#Exposome Austria, Research Infrastructure and National EIRENE Node, Vienna, Austria

Putative annotation of compounds: MS^2 matching

Putatively annotated compounds via MS^2 matching with open-source libraries are showcased for SRM 1950 1958 in both ESI+ and ESI- modes (Levels 3/2b). The same procedure was used to evaluate the performance for SRM 1958 and, since results were very comparable to SRM 1950, we here chose to present the data for SRM 1950 only in order to avoid extensive and repetitive data description.

The purpose of these comparisons is to demonstrate how MRM+SWATH and SWATH-only methods perform similarly. Therefore, in this case, the annotation parameters (mass error, dot product, reverse dot product, score, visual MS^2 matching) are of higher relevance than the compound match itself (i.e., confidence level for identification, possibility of different isomers, etc).

In all mirror plots, experimental spectra are depicted in blue colour (upper panel) and reference spectra in red colour (lower panel). Darker lines in the showcased spectra represent matched ions.

Table S9. Dot and reverse dot scores for annotated compounds in ESI- mode. MS^2 spectra were matched against open source libraries as described in material and methods. Mirror plots for each compound match is described in figures S1 to S26.

NEGATIVE MODE (ESI-)				
Putative annotation	SWATH-only		MRM+SWATH	
	Dot	Reverse Dot	Dot	Reverse Dot
**S1**. Citric acid	437	550	566	625
**S2**. Glutamic acid	826	918	898	904
**S3**. Glutamine	332	878	807	882
**S4**. Histidine	223	870	320	837
**S5**. Tyrosine	285	895	295	904
**S6**. 4-acetamidophenol sulfate	930	876	950	869
**S7**. Phenylalanine	825	873	829	889
**S8**. 3-hydroxybenzoic acid	958	879	987	879
**S9**. Phenylacetylglutamine	831	715	804	655
**S10**. Phenol sulfate	833	750	925	851
**S11**. Alpha-hydroxyhippuric acid	958	866	901	826
**S12**. Tryptophan	940	873	978	935
**S13**. Indolacetic acid	767	873	570	870
**S14**. 2,4,6-Trimethylbenzoic acid	809	773	888	809
**S15**. Acetaminophen (Paracetamol)	890	869	951	863
**S16**. Theophylline	940	869	972	872
**S17**. Indoxyl sulfate	468	644	502	684
**S18**. p-cresol sulfate	969	745	984	743
**S19**. 2-Naphthalenesulfonic acid	923	861	890	814
**S20**. lycochenodeoxycholic acid	747	828	655	688
**S21**. 4-nitrophenol	762	749	843	713
**S22**. Glycocholic acid	855	894	918	750
**S23**. Cortisol	711	855	619	813
**S24**. Indole-3-acetaldehyde	874	860	900	874
**S25**. FA 18:2+2O	440	901	662	794
**S26**. LPE 18:1	675	766	688	770

Table S10. Dot and reverse dot scores for annotated compounds in ESI+ mode. MS^2 spectra were matched against open source libraries as described in material and methods. Mirror plots for each compound match is described in figures S27 to S45.

POSITIVE MODE (ESI+)				
**Putative annotation**	**SWATH-only**		**MRM+SWATH**	
	**Dot**	**Reverse Dot**	**Dot**	**Reverse Dot**
**S27**. Glutamic acid	902	900	948	927
**S28**. Acetylcarnitine	537	860	674	804
**S29**. Methionine	226	595	778	819
**S30**. Tyrosine	186	869	171	795
**S31**. Isoleucine	855	874	915	872
**S32**. Leucine	999	880	999	880
**S33**. Phenylalanine	961	948	972	967
**S34**. Indoline	976	735	974	742
**S35**. Theobromine	896	865	822	863
**S36**. Tryptophan	894	935	894	847
**S37**. 3-Formylindole	883	845	969	856
**S38**. Acetaminophen (Paracetamol)	969	856	827	811
**S39**. Theophylline	971	861	916	859
**S40**. Indole-3-acetamide	513	595	371	419
**S41**. Glycoursodeoxycholic acid	951	967	756	862
**S42**. 1-pentadecanoyl-2-hydroxy-sn-glycero-3-phosphocholine	433	814	390	796
**S43**. 1-palmitoyl-2-hydroxy-sn-glycero-3-phosphoethanolamine	797	832	888	850
**S44**. Di-n-butyl phthalate	988	875	982	868
**S45**. Bis(2-ethylhexyl) phthalate	679	729	692	743

ESI- MODE

1. Citric acid (*m/z* 191.0197, RT 0.58 min)

SWATH-only

Precursor mass error: 0 ppm

(Dot: 437, Rev: 550, Total Score:1.2)

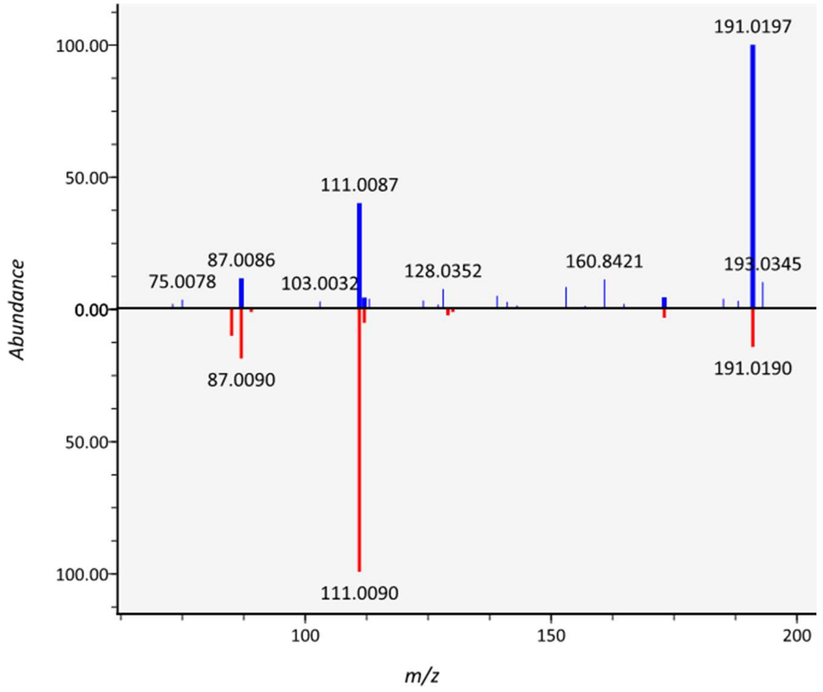

MRM+SWATH

Precursor mass error: 0.5 ppm

(Dot: 566, Rev: 625, Total Score: 1.4)

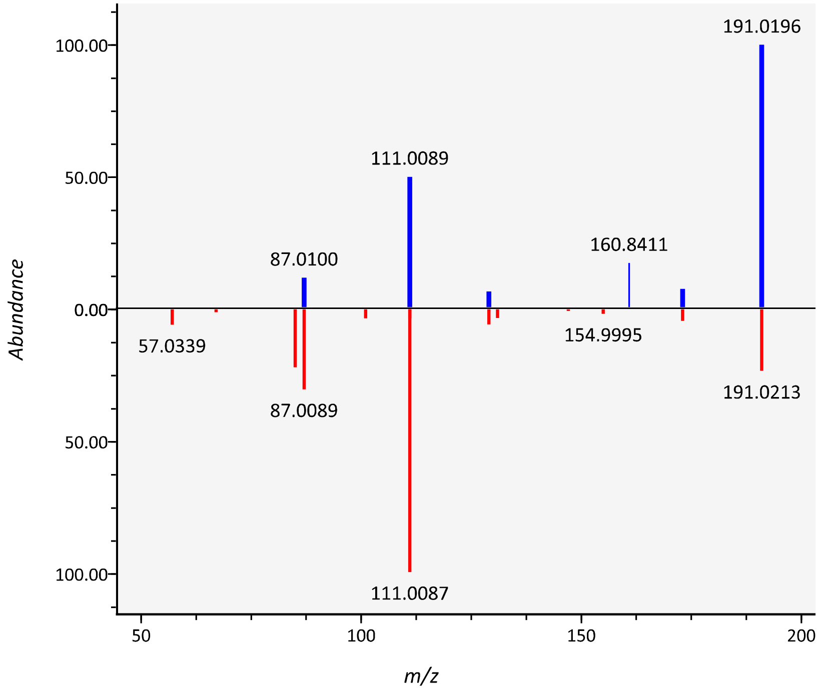

2. Glutamic acid (m/z 146.0457, RT 0.59 min)

<u>SWATH-only</u>

Precursor mass error: 1.4 ppm

(Dot: 826, Rev: 918, Total Score: 1.8)

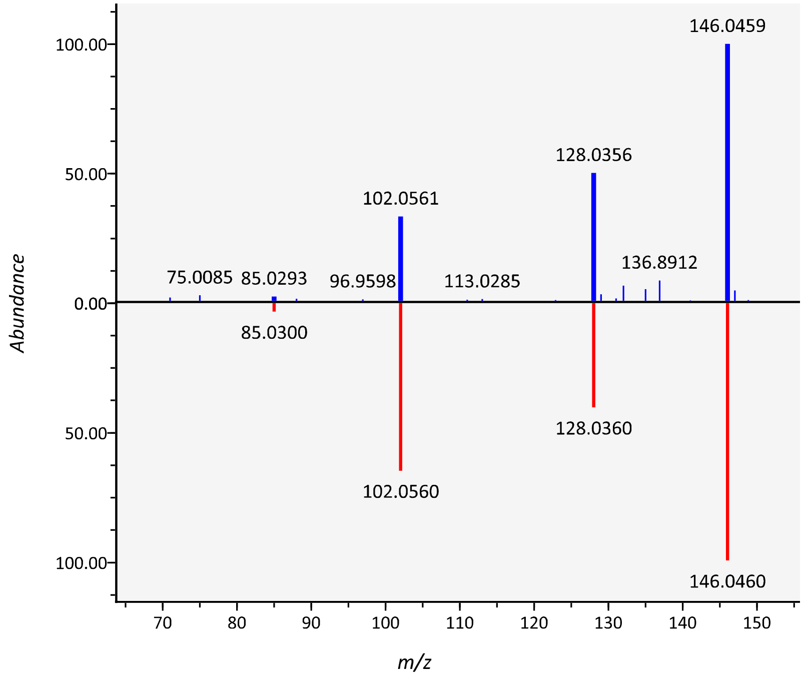

<u>MRM+SWATH</u>

Precursor mass error: 0.7 ppm

(Dot: 898, Rev: 904, Total Score: 1.8)

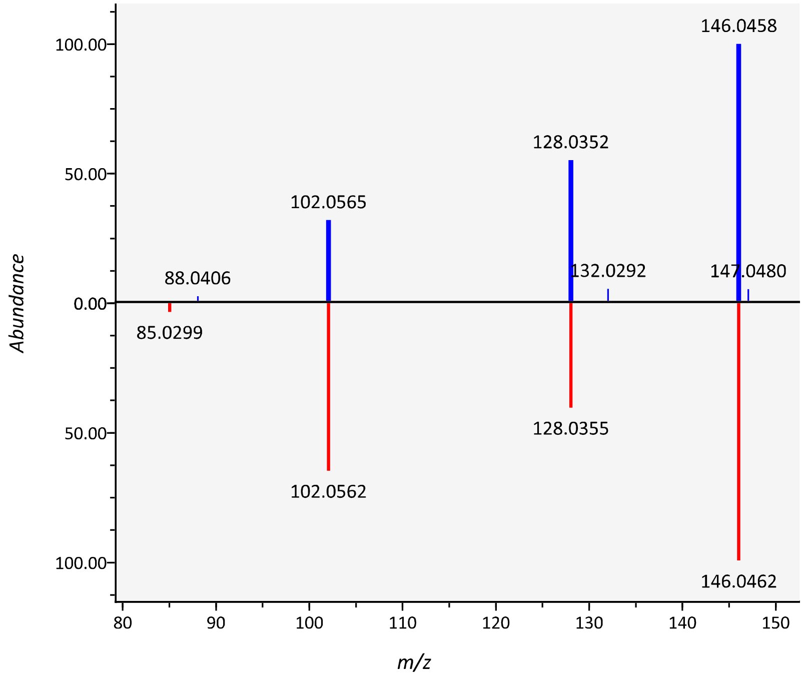

3. L-Glutamine (m/z 145.0619, RT 0.66 min)

SWATH-only

Precursor mass error: 0.7 ppm

(Dot:332, Rev: 878, Total Score: 1.3)

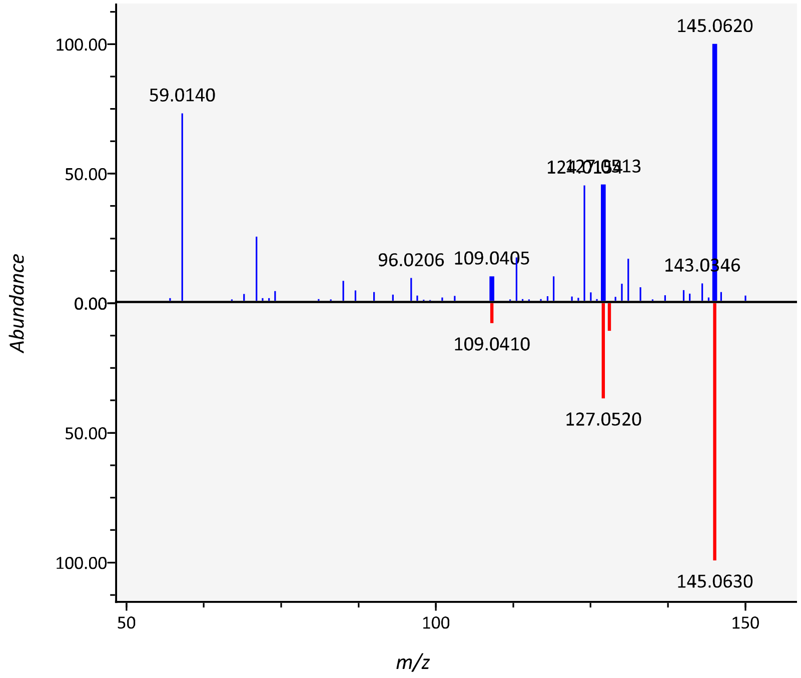

MRM+SWATH

Precursor mass error: 0 ppm

(Dot: 807, Rev: 882, Total Score: 1.7)

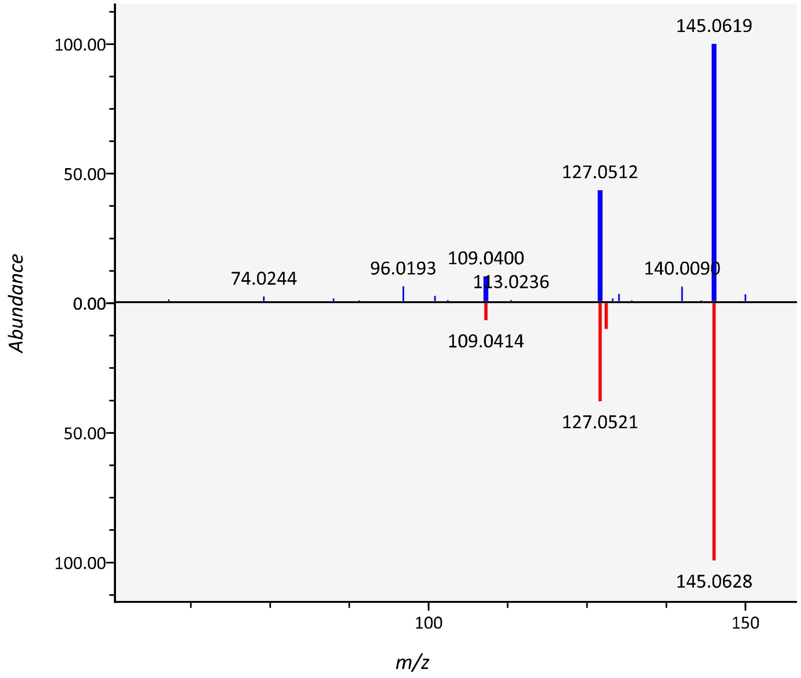

4. L-Histidine (m/z 154.0622, RT 0.71 min)

SWATH-only

Precursor mass error: 1.3 ppm

(Dot: 223, Rev: 870, Total Score: 1.2)

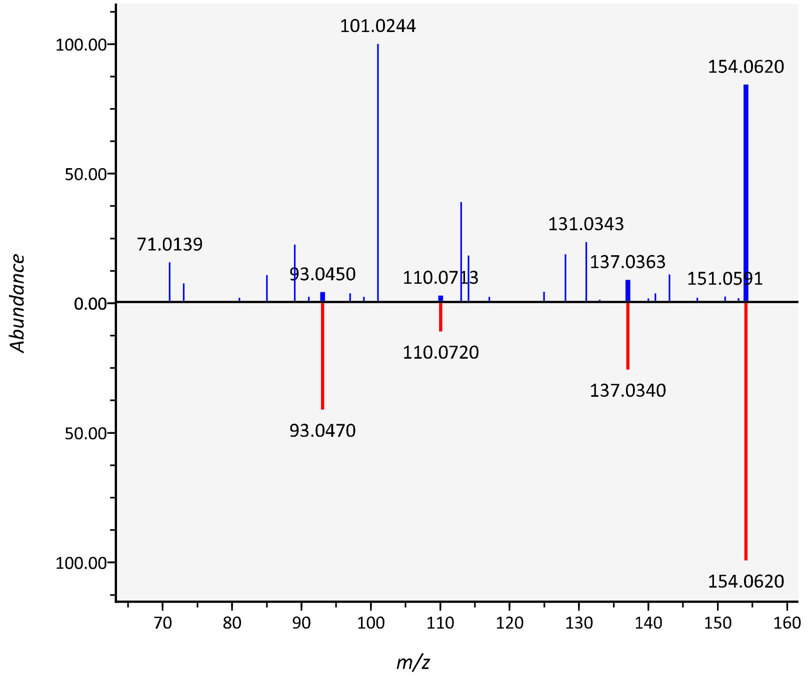

MRM+SWATH

Precursor mass error: 1.3 ppm

(Dot: 320, Rev: 837, Total Score: 1.3)

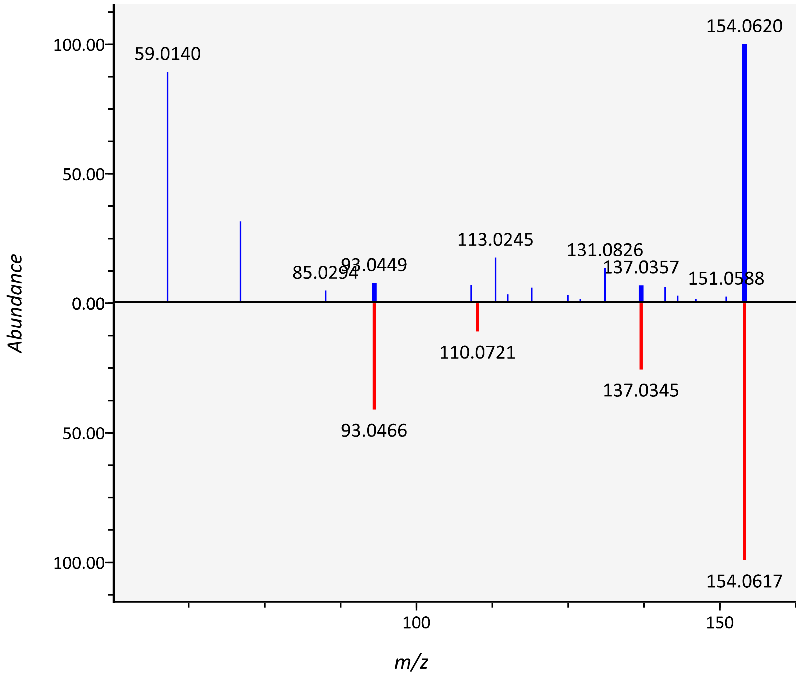

5. Tyrosine (m/z 180.0666, RT 0.92 min)

<u>SWATH-only</u>

Precursor mass error: 1.1 ppm

(Dot: 285, Rev: 895, Total Score: 1.3)

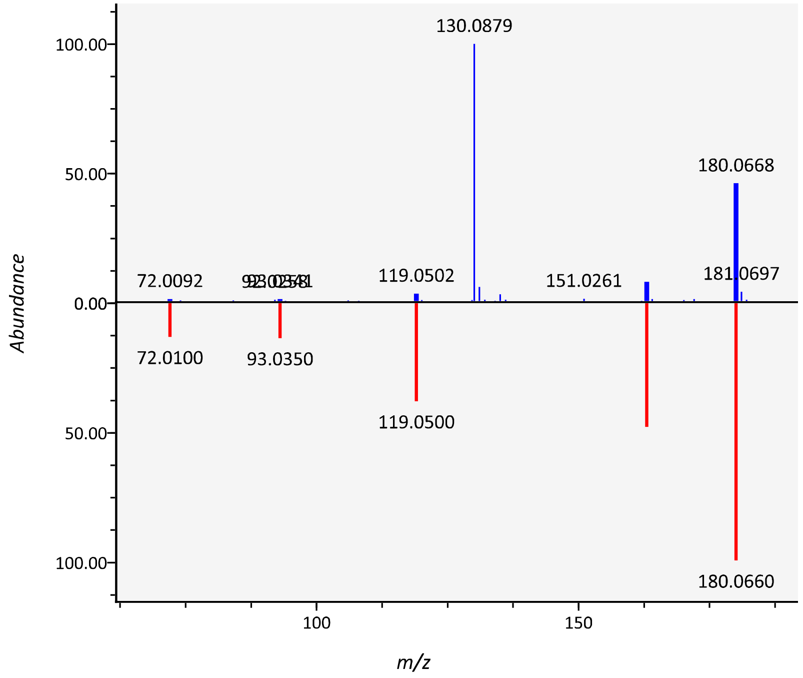

<u>MRM+SWATH</u>

Precursor mass error: 0.5 ppm

(Dot: 295, Rev: 904, Total Score: 1.3)

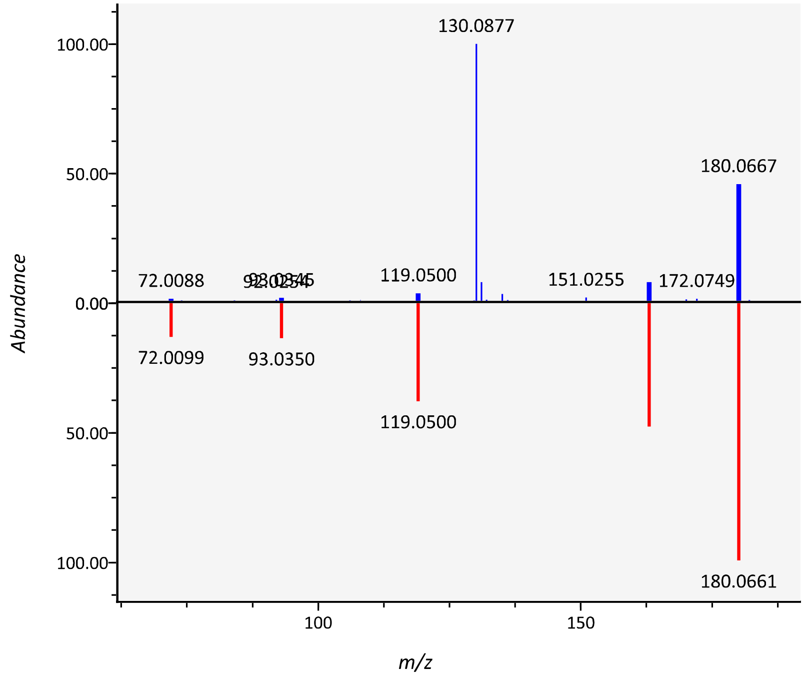

6. 4-acetamidophenol sulfate (m/z 230.0129, RT 1.66 min)

<u>SWATH-only</u>

Precursor mass error: 1.7 ppm

(Dot: 930, Rev: 876, Total Score: 1.8)

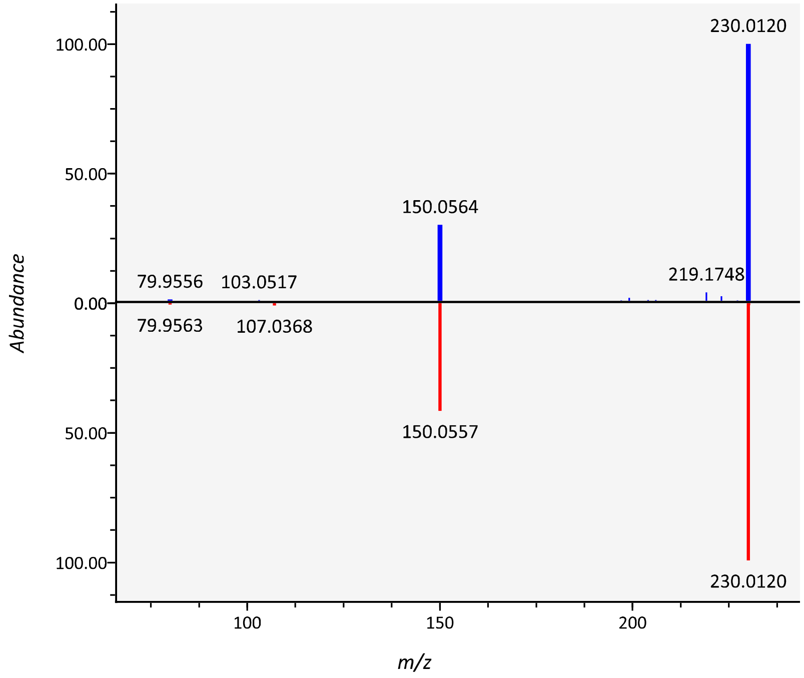

<u>MRM+SWATH</u>

Precursor mass error: 2.2 ppm

(Dot: 950, Rev: 869, Total Score: 1.9)

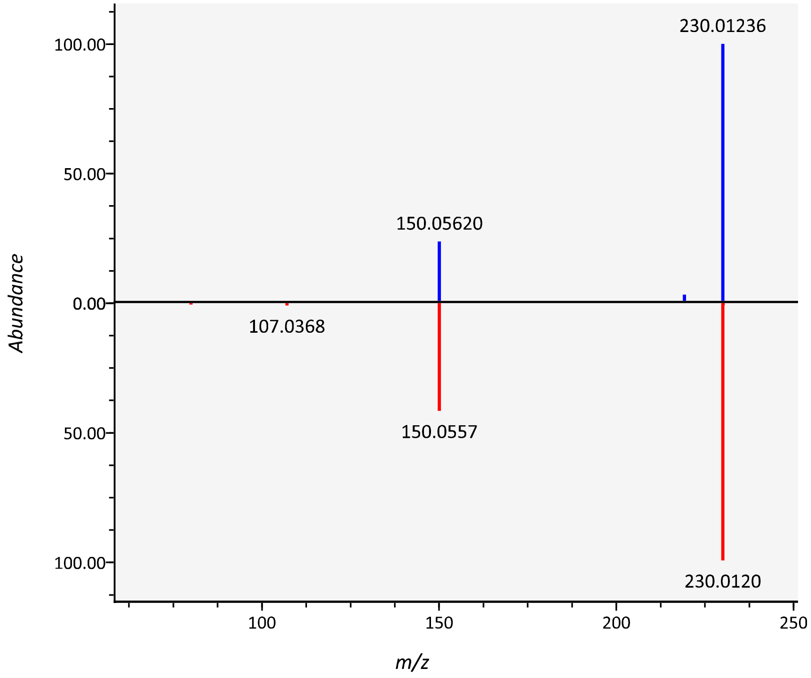

7. L-phenylalanine (m/z 164.0717, RT 1.69 min)

SWATH-only

Precursor mass error: 1.8 ppm

(Dot: 825, Rev: 873, Total Score: 1.8)

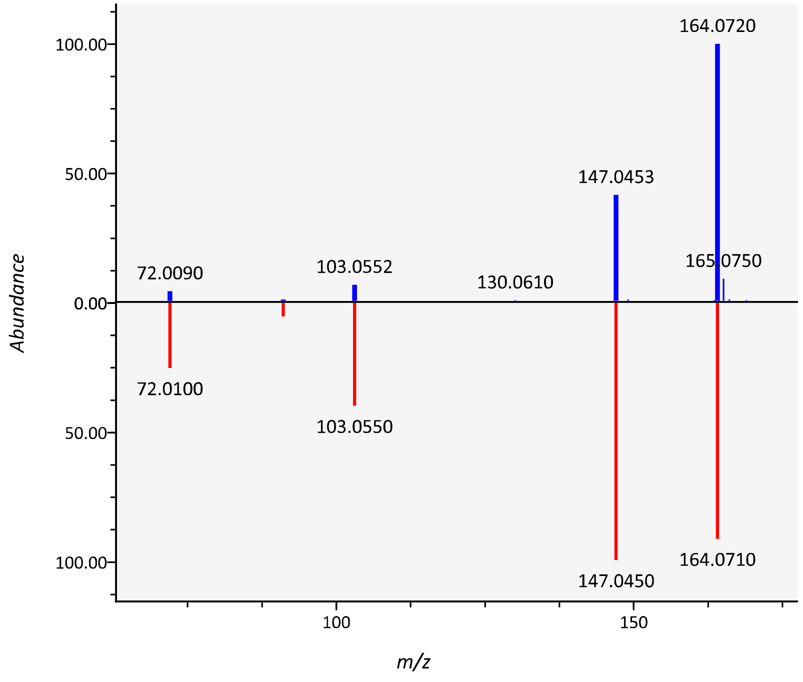

MRM+SWATH

Precursor mass error: 0.6 ppm

(Dot: 829, Rev: 889, Total Score: 1.8)

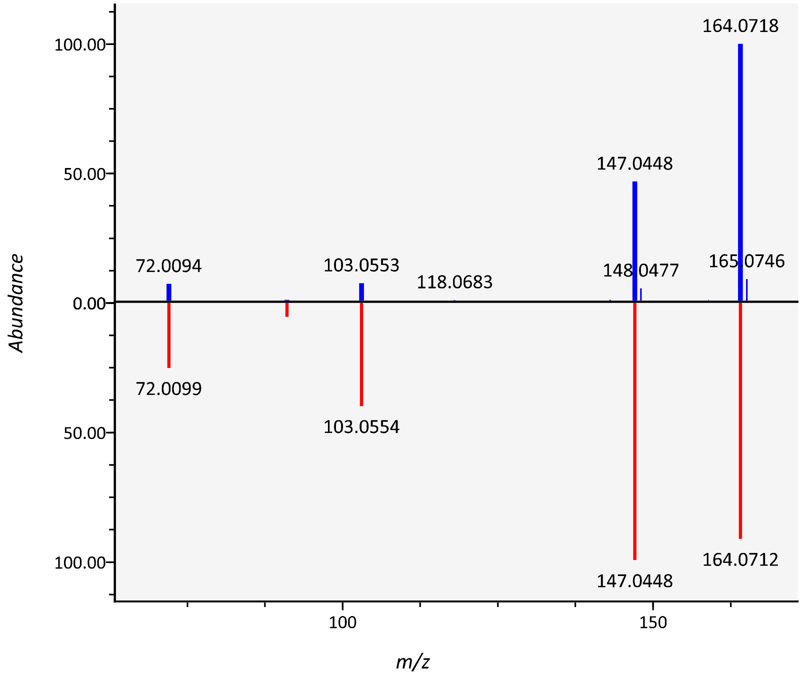

8. 3-hydroxybenzoic acid (m/z 137.0244, RT 2.45 min)

<u>SWATH-only</u>

Precursor mass error: 1.5 ppm

(Dot: 958, Rev: 879, Total Score: 1.9)

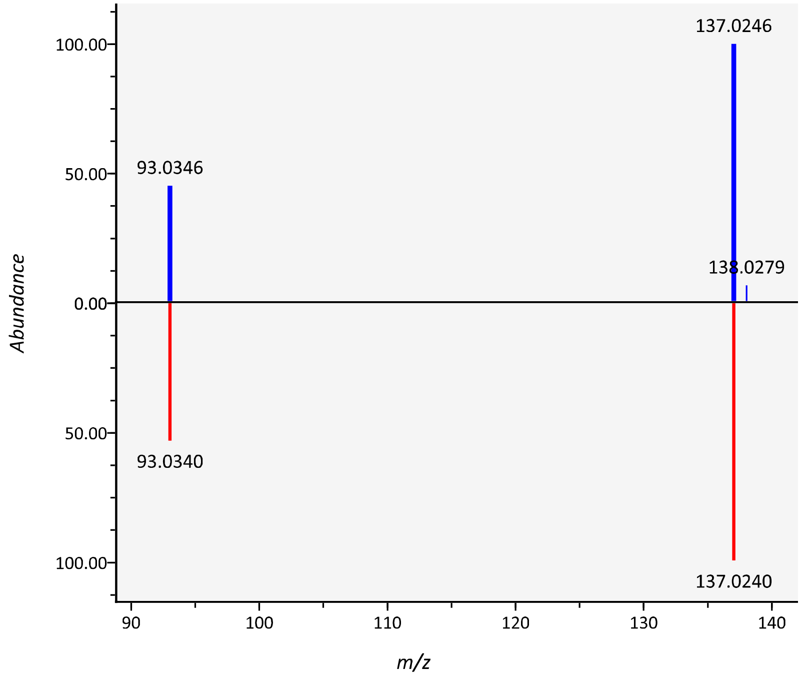

<u>MRM+SWATH</u>

Precursor mass error: 4.4 ppm

(Dot: 987, Rev: 879, Total Score: 1.9)

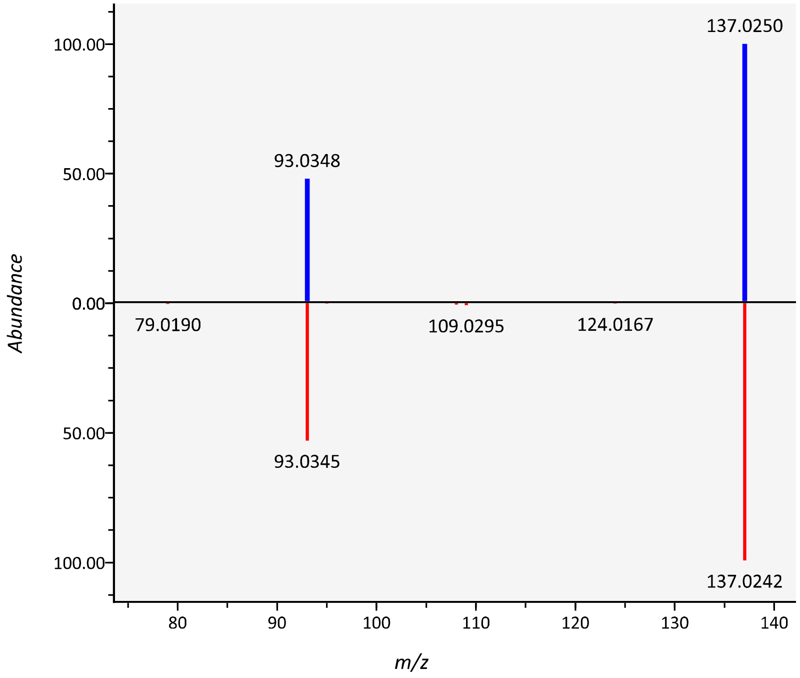

9. Phenylacetylglutamine (m/z 263.1037, RT 2.68 min)

<u>SWATH-only</u>

Precursor mass error: 1.1 ppm

(Dot: 831, Rev: 715, Total Score: 1.6)

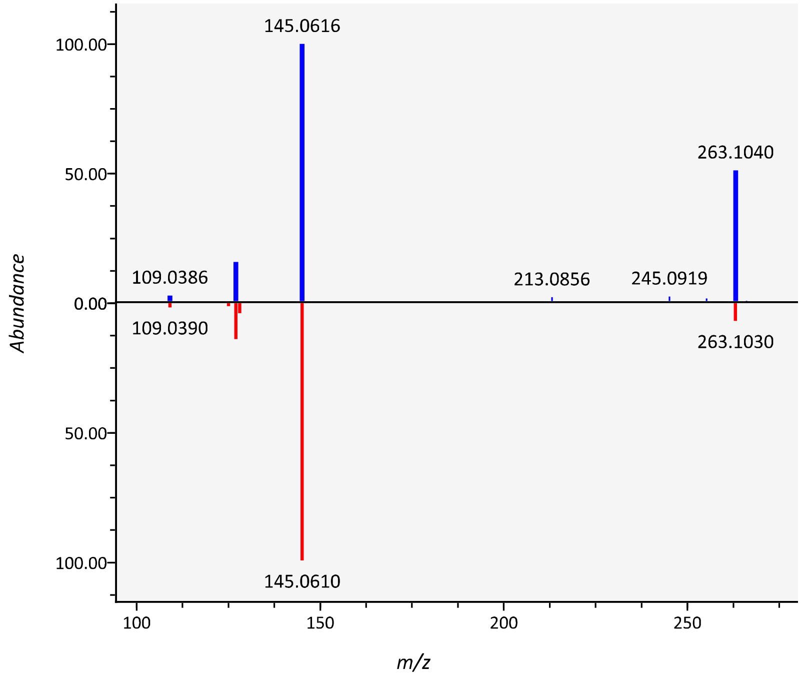

<u>MRM+SWATH</u>

Precursor mass error: 0 ppm

(Dot: 804, Rev: 655, Total Score: 1.6)

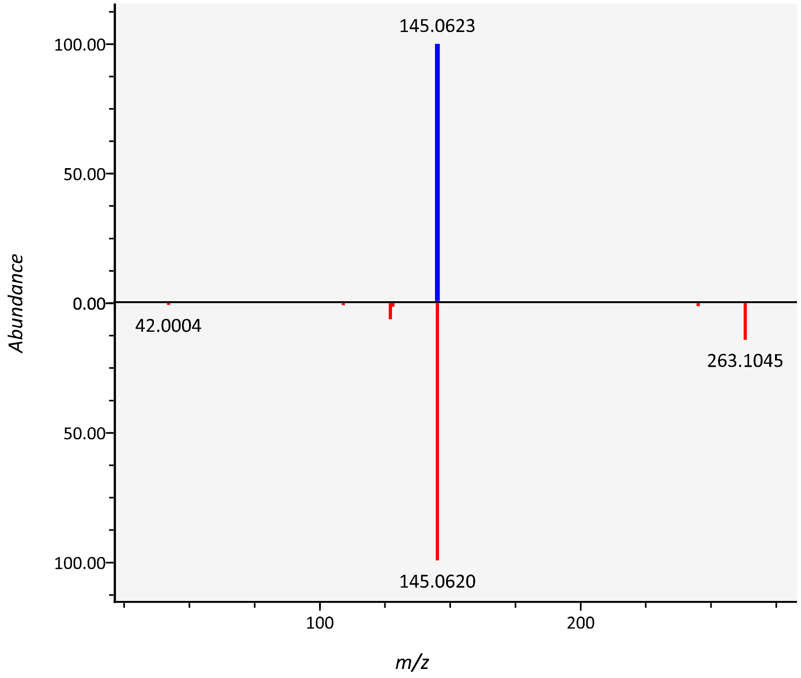

10. Phenol sulfate (m/z 172.9914, RT 2.85 min)

SWATH-only

Precursor mass error: 1.2 ppm

(Dot: 833, Rev: 750, Total Score: 1.7)

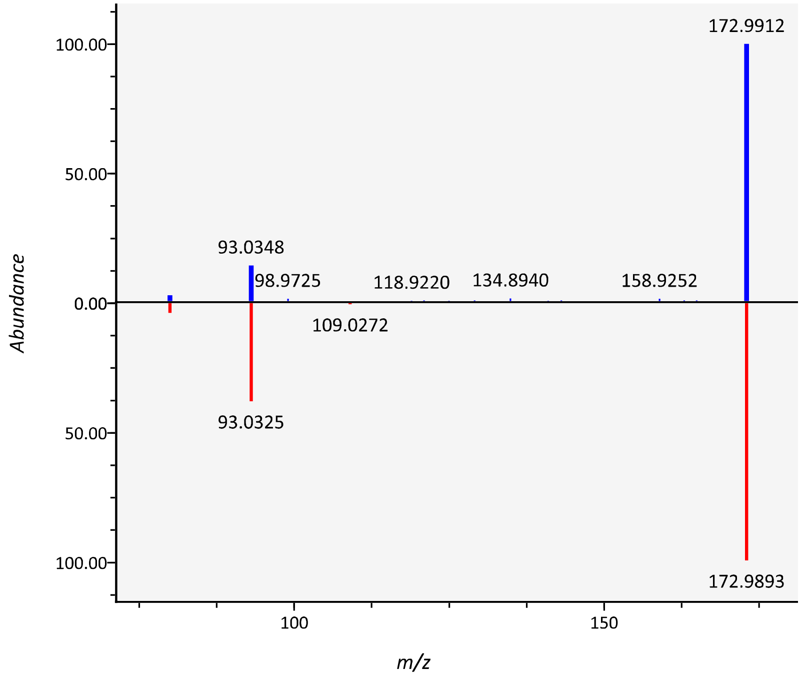

MRM+SWATH

Precursor mass error: 0.6 ppm

(Dot: 925, Rev: 851, Total Score: 1.8)

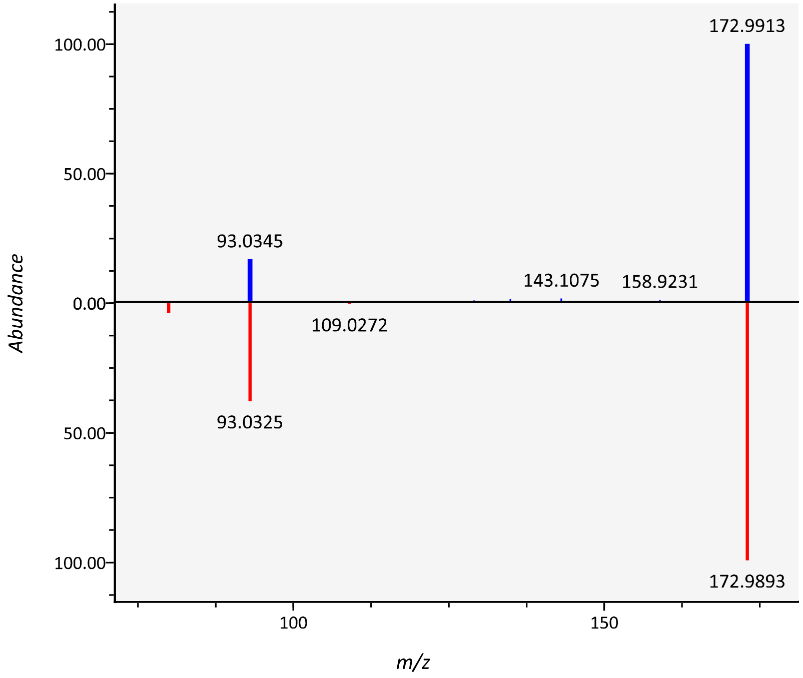

11. alpha-Hydroxyhippuric acid (m/z 194.0459, RT 2.92 min)

<u>SWATH-only</u>

Precursor mass error: 4.6 ppm

(Dot: 958, Rev: 866, Total Score: 1.9)

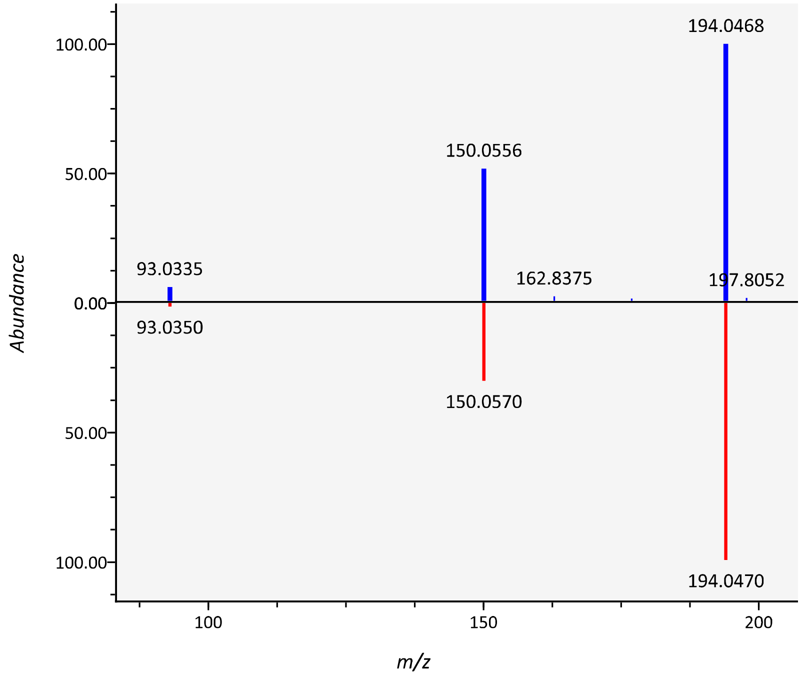

<u>MRM+SWATH</u>

Precursor mass error: 2.0 ppm

(Dot: 901, Rev: 826, Total Score: 1.8)

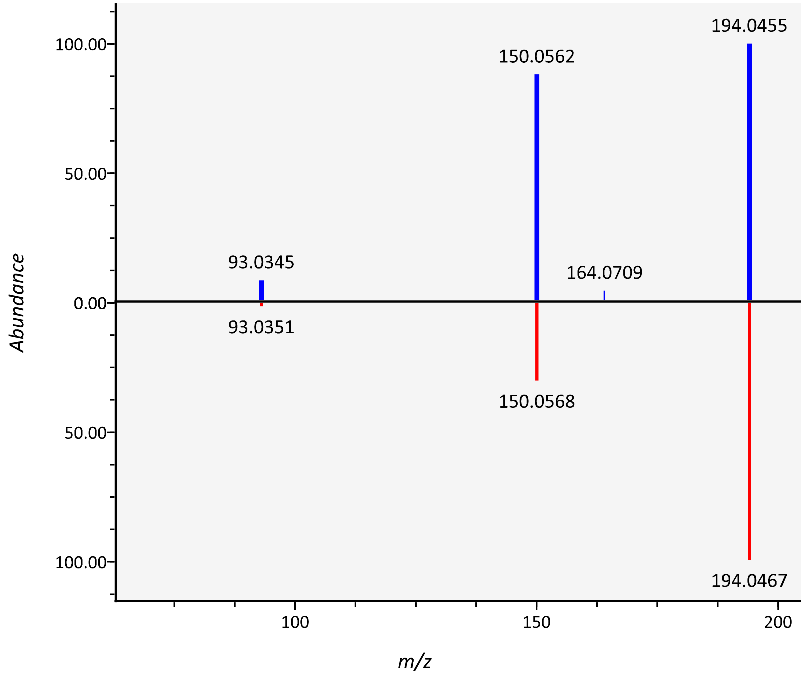

12. Tryptophan (m/z 203.0826, RT 3.00 min)

SWATH-only

Precursor mass error: 1.5 ppm

(Dot: 940, Rev: 873, Total Score: 1.9)

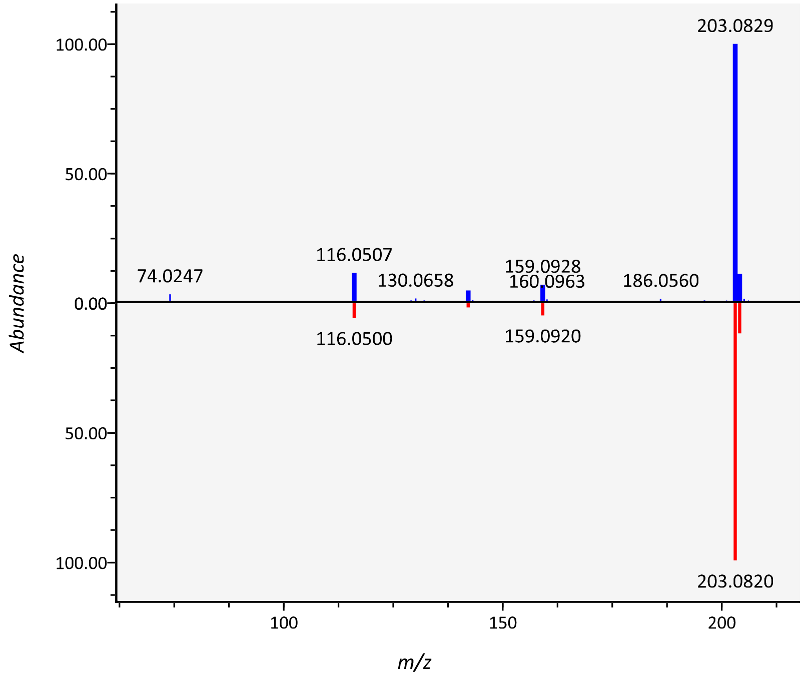

MRM+SWATH

Precursor mass error: 3.9 ppm

(Dot: 978, Rev: 935, Total Score: 1.9)

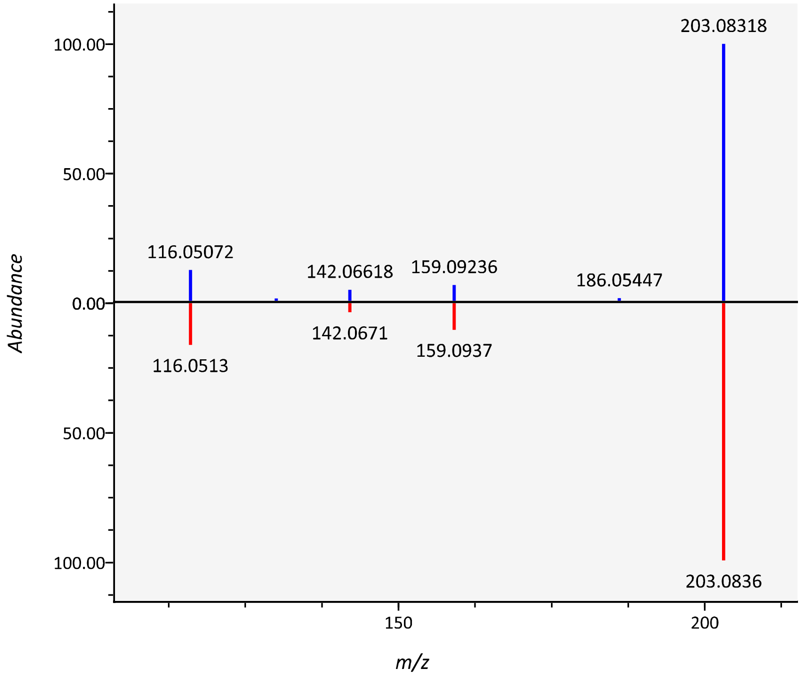

13. Indolacetic acid (m/z 174.0561, RT 3.00 min)

<u>SWATH-only</u>

Precursor mass error: 0 ppm

(Dot: 767, Rev: 873, Total Score: 1.7)

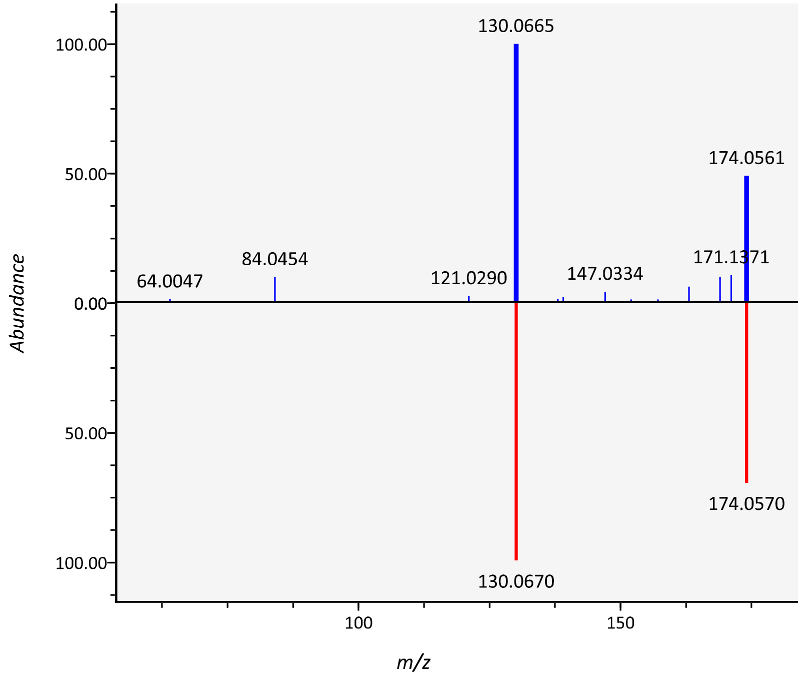

<u>MRM+SWATH</u>

Precursor mass error: 7.5 ppm

(Dot: 570, Rev: 870, Total Score: 1.5)

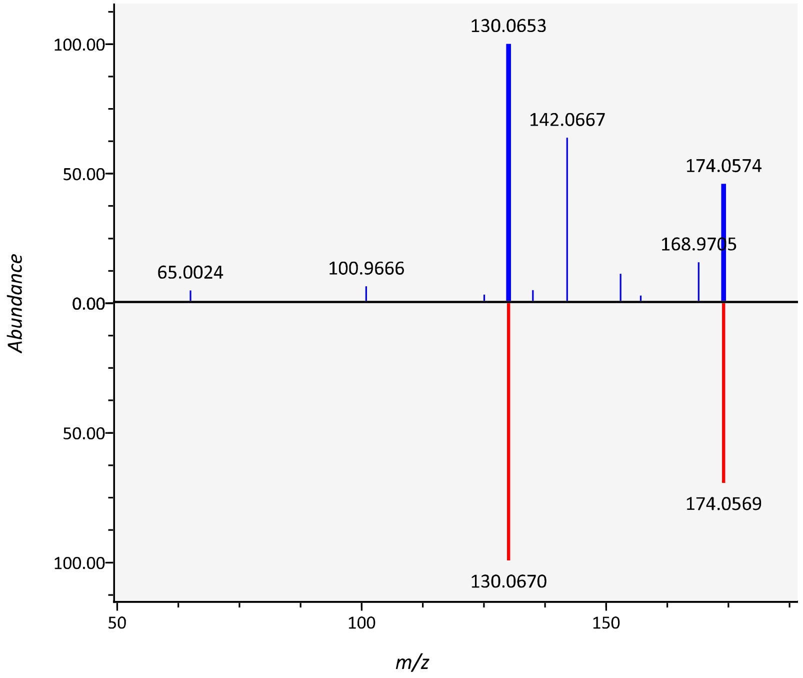

14. 2,4,6-Trimethylbenzoic acid (m/z 163.0765, RT 3.00 min)

SWATH-only

Precursor mass error: 0.6 ppm

(Dot: 809, Rev: 773, Total Score: 1.7)

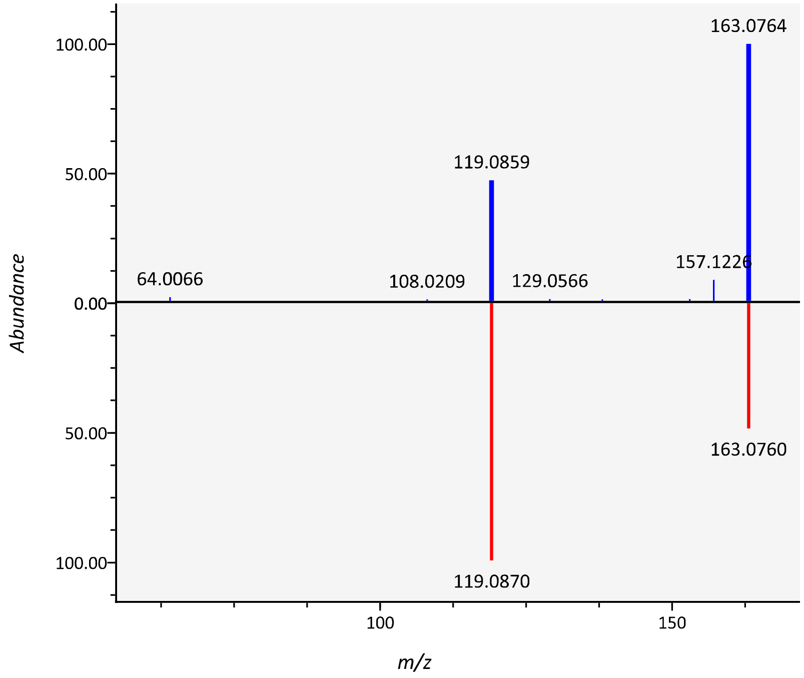

MRM+SWATH

Precursor mass error: 2.5 ppm

(Dot: 888, Rev: 809, Total Score: 1.8)

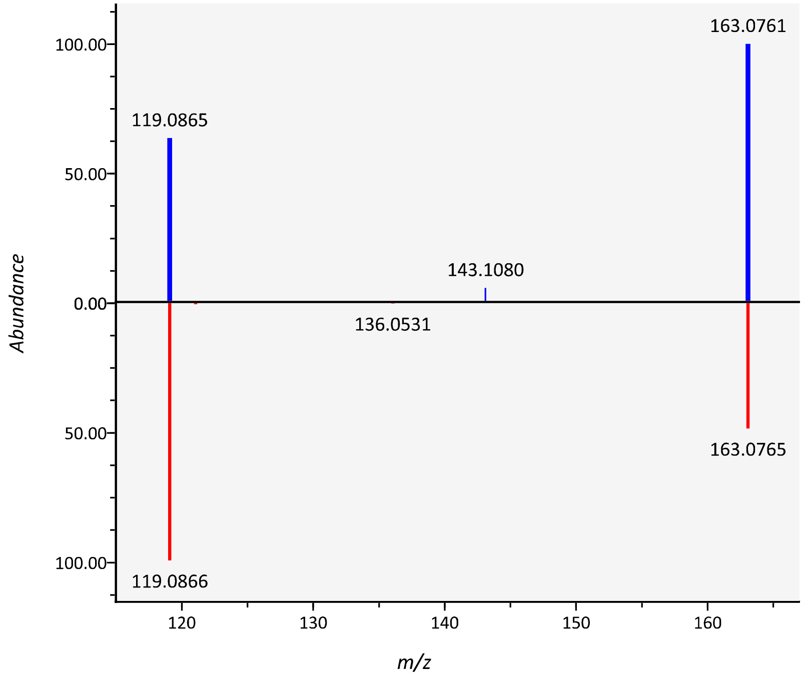

15. Acetaminophen / Paracetamol (m/z 150.0561, RT 3.10 min) – ID confirmed with standard (Level 1)

SWATH-only

Precursor mass error: 1.3 ppm

(Dot: 890, Rev: 869, Total Score: 1.8)

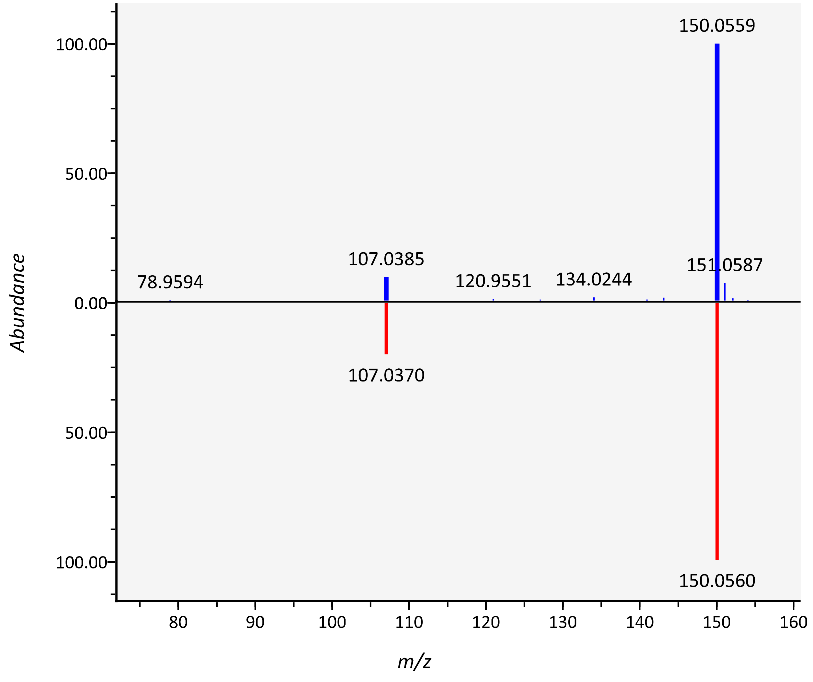

MRM+SWATH

Precursor mass error: 0.7 ppm

(Dot: 951, Rev: 863, Total Score: 1.9)

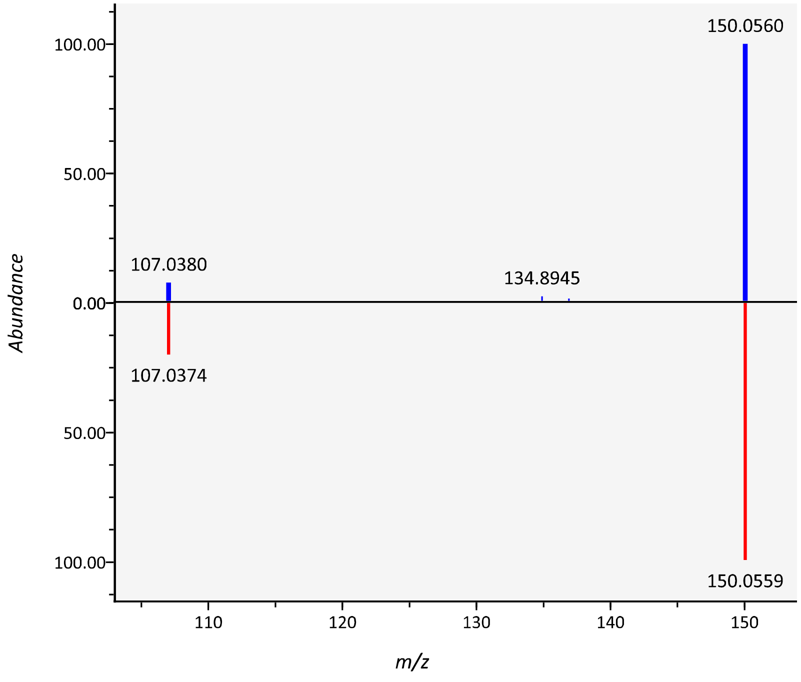

16. Theophylline (m/z 179.0574, RT 3.24 min)

SWATH-only

Precursor mass error: 1.1 ppm

(Dot: 940, Rev: 869, Total Score: 1.9)

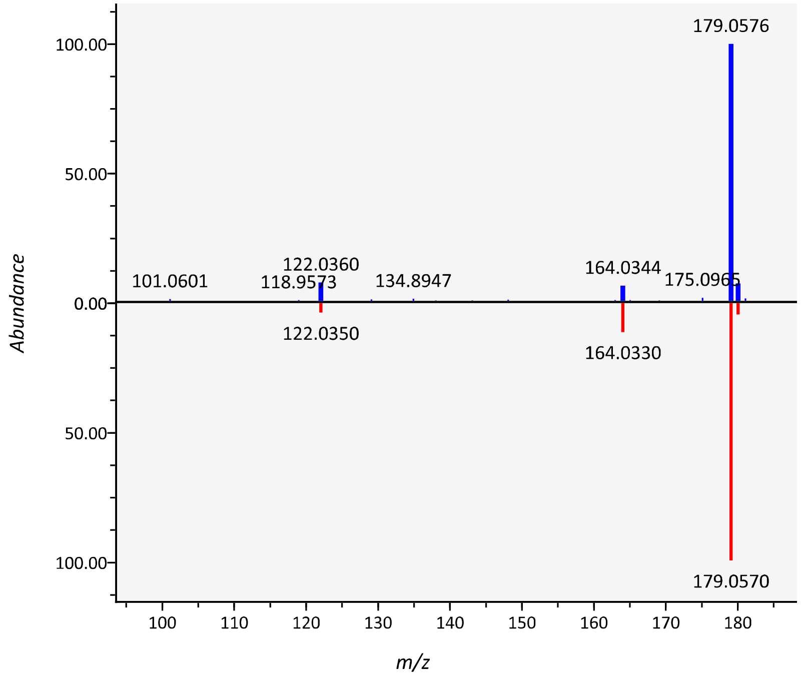

MRM+SWATH

Precursor mass error: 0.6 ppm

(Dot: 972, Rev: 872, Total Score: 1.9)

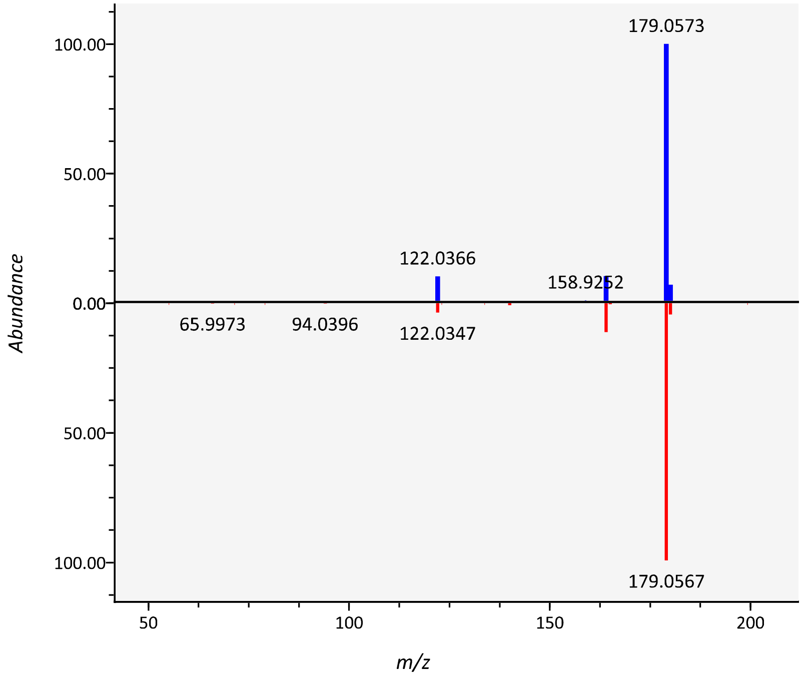

17. Indoxyl sulfate (m/z 212.0023, RT 3.26 min)

<u>SWATH-only</u>

Precursor mass error: 0.5 ppm

(Dot: 468, Rev: 644, Total Score: 1.4)

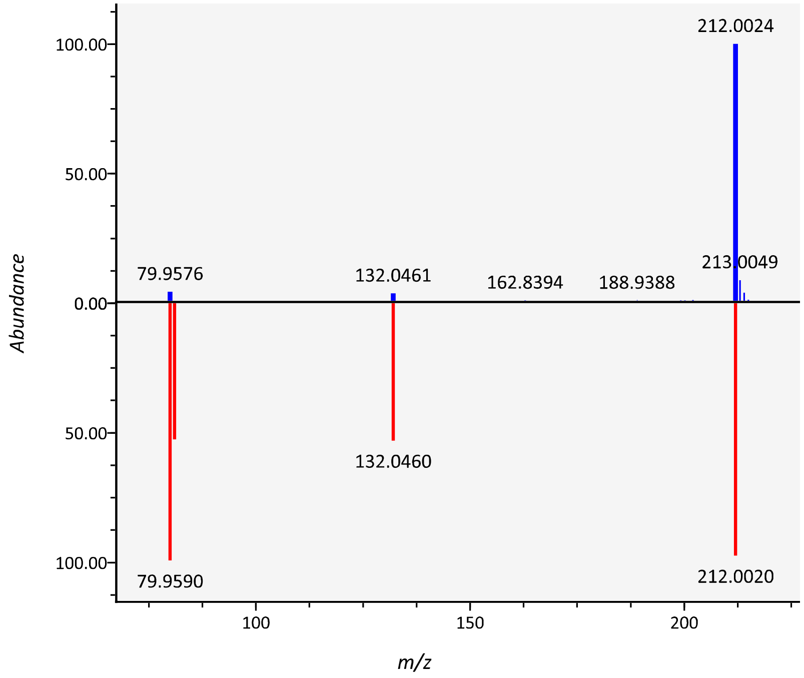

<u>MRM+SWATH</u>

Precursor mass error: 1.4 ppm

(Dot: 502, Rev: 684, Total Score: 1.5)

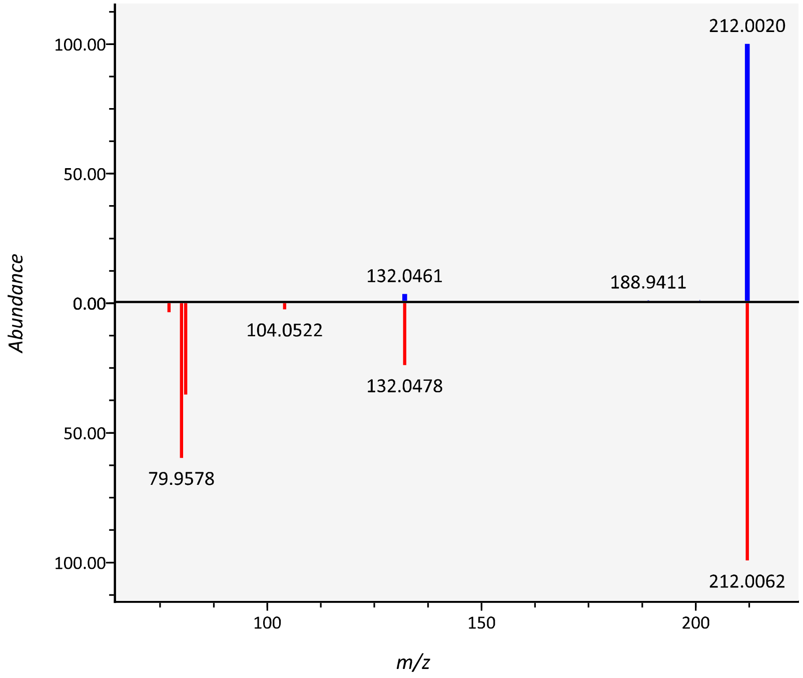

18. p-cresol sulfate (m/z 187.0071, RT 3.68 min)

<u>SWATH-only</u>

Precursor mass error: 1.6 ppm

(Dot: 969, Rev: 745, Total Score: 1.8)

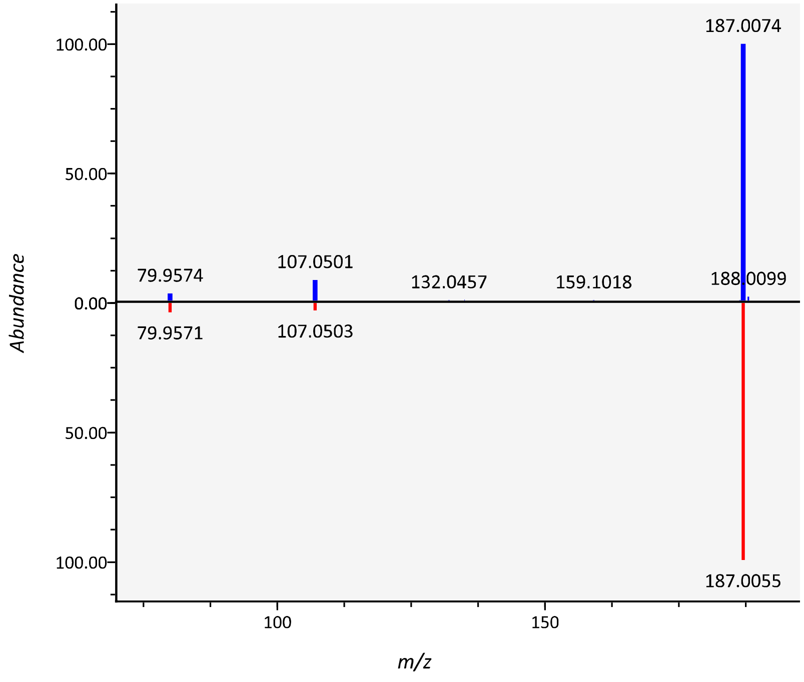

<u>MRM+SWATH</u>

Precursor mass error: 3.2 ppm

(Dot: 984, Rev: 743, Total Score: 1.8)

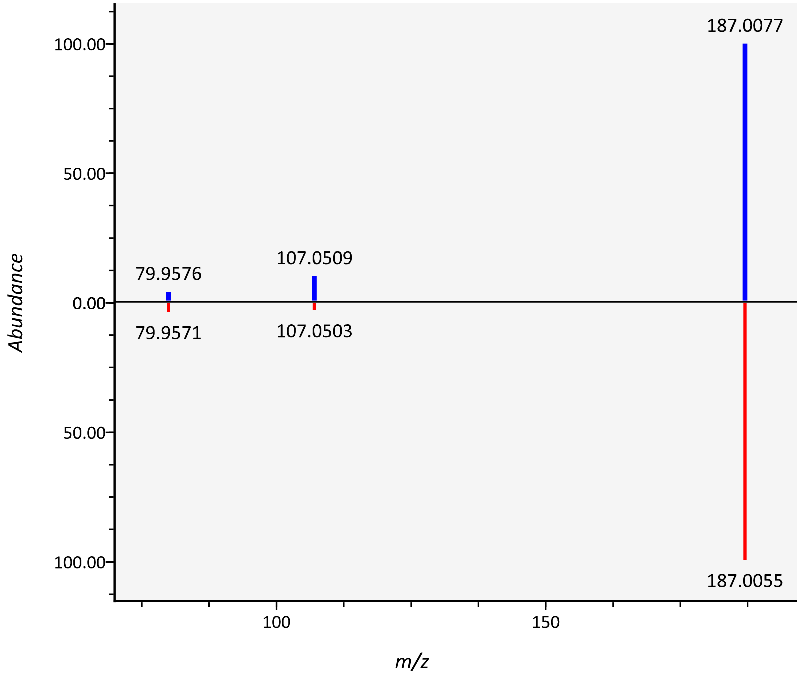

19. 2-Naphthalenesulfonic acid (m/z 207.0121, RT 3.80 min)

<u>SWATH-only</u>

Precursor mass error: 1.4 ppm

(Dot: 923, Rev: 861, Total Score: 1.8)

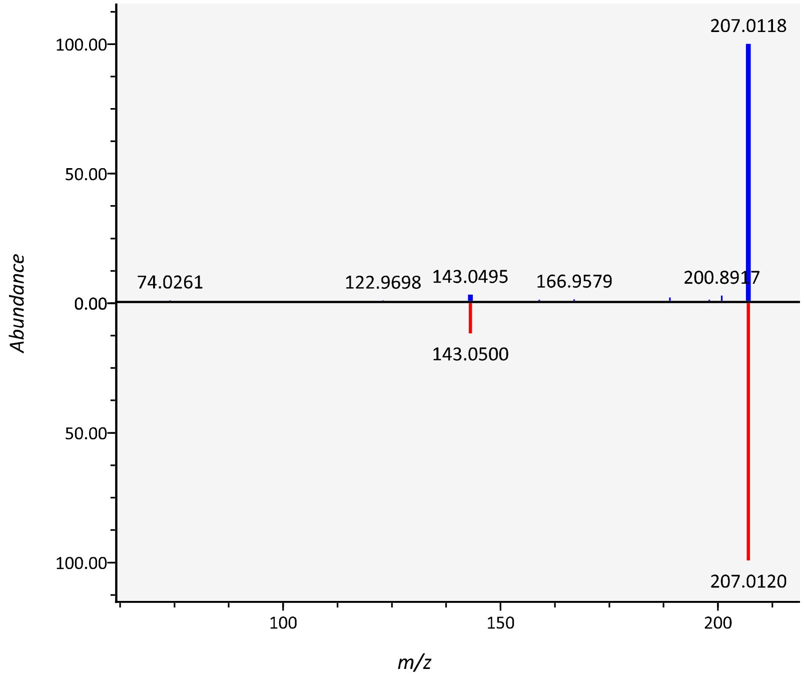

<u>MRM+SWATH</u>

Precursor mass error: 0.5 ppm

(Dot: 890, Rev: 814, Total Score: 1.7)

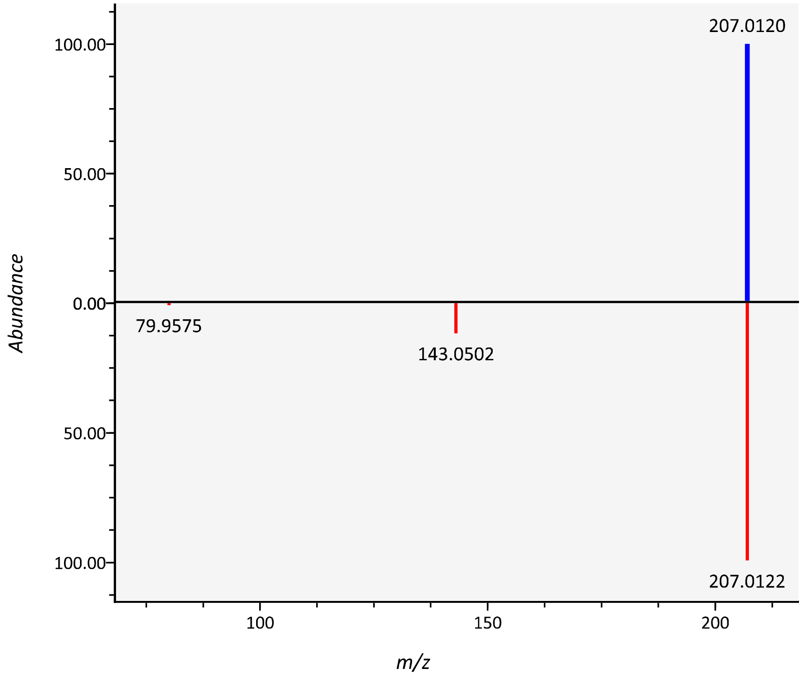

20. Glycochenodeoxycholic acid (m/z 448.3068, RT 5.63 min)

<u>SWATH-only</u>

Precursor mass error: 1.8 ppm

(Dot: 747, Rev: 828, Total Score: 1.7)

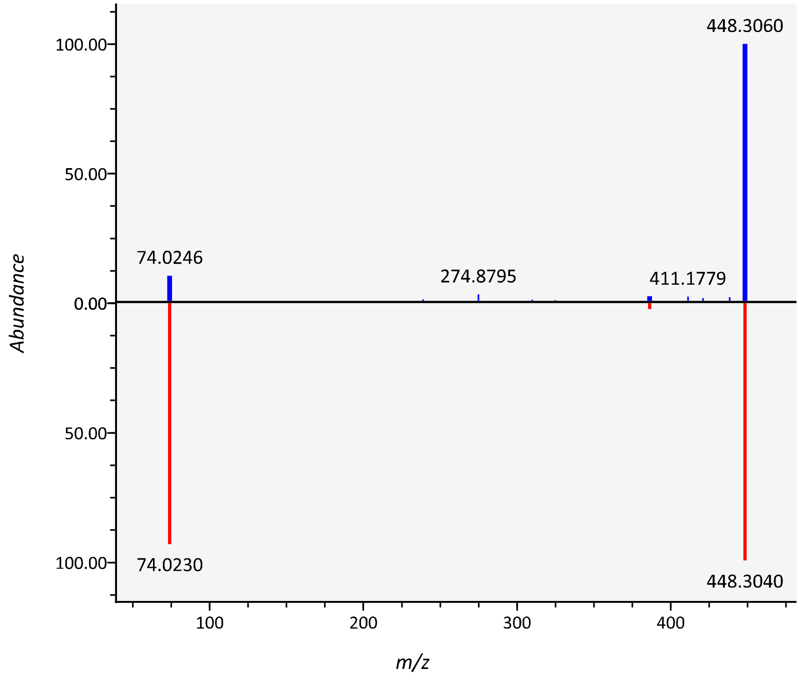

<u>MRM+SWATH</u>

Precursor mass error: 2.9 ppm

(Dot: 655, Rev: 688, Total Score: 1.7)

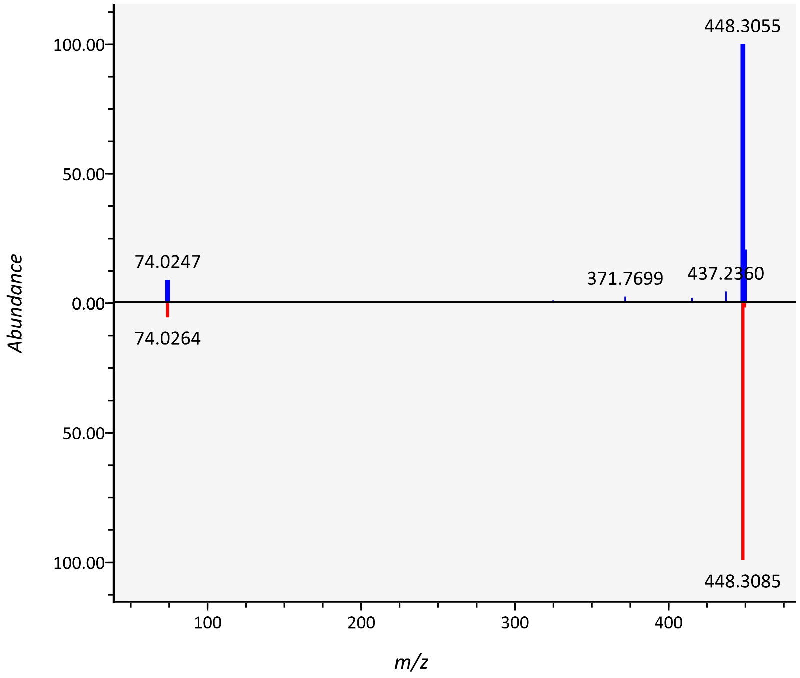

21. 4-nitrophenol (m/z 138.0197, RT 5.64 min)

<u>SWATH-only</u>

Precursor mass error: 1.4 ppm

(Dot: 762, Rev: 749, Total Score: 1.6)

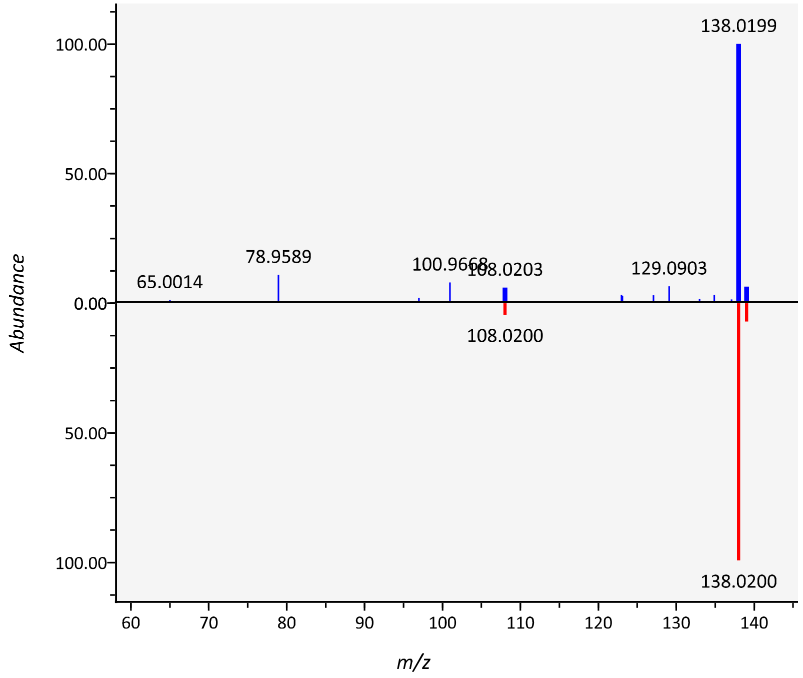

<u>MRM+SWATH</u>

Precursor mass error: 2.9 ppm

(Dot: 843, Rev: 713, Total Score: 1.6)

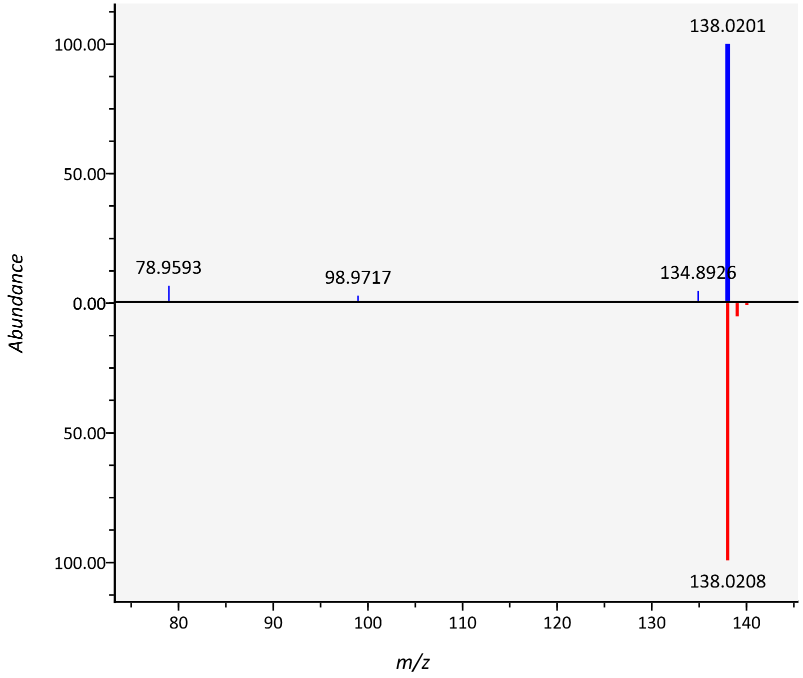

22. Glycocholic acid (m/z 464.3018, RT 5.74 min)

<u>SWATH-only</u>

Precursor mass error: 0.4 ppm

(Dot: 855, Rev: 894, Total Score: 1.8)

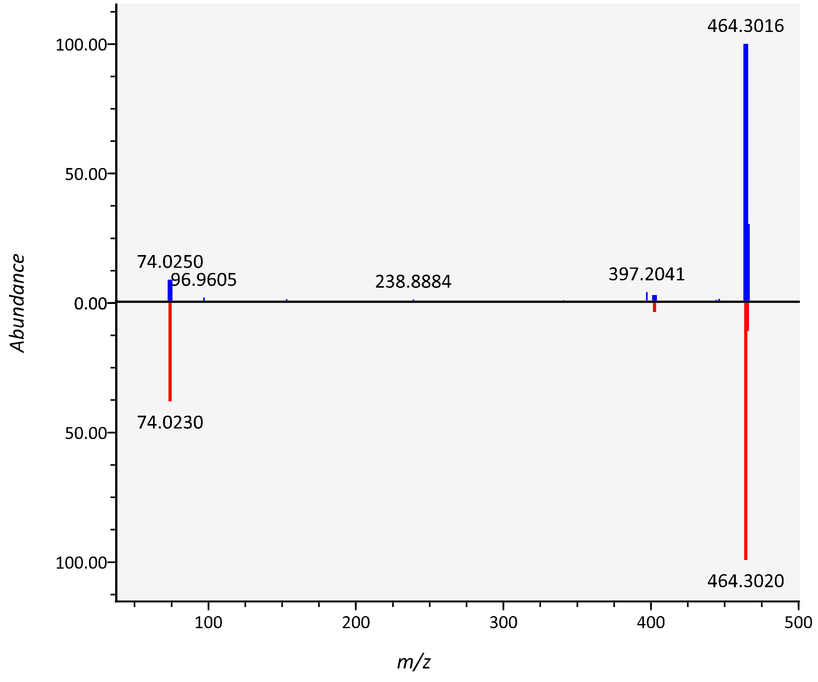

<u>MRM+SWATH</u>

Precursor mass error: 0.9 ppm

(Dot: 918, Rev: 750, Total Score: 1.7)

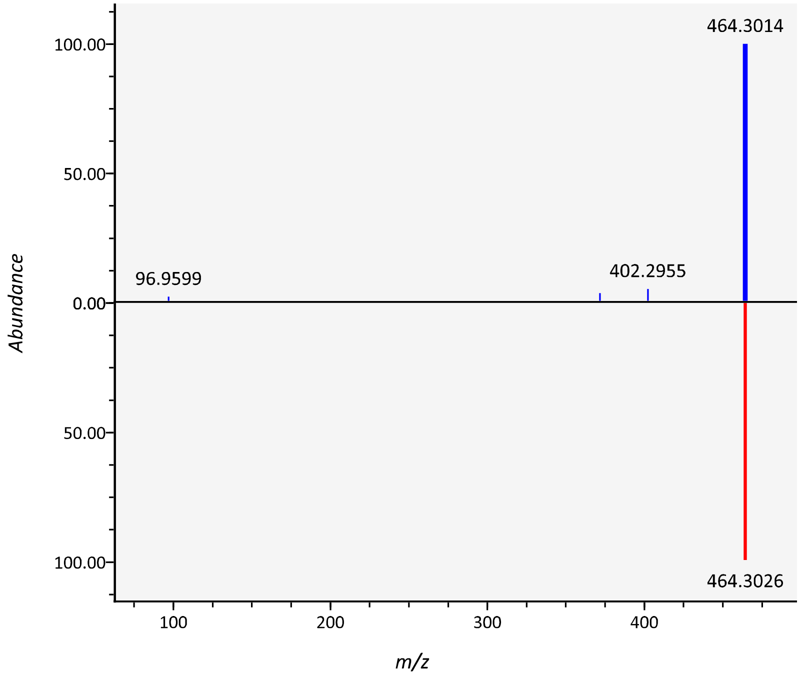

23. Cortisol (m/z 361.2020 RT 6.20 min)

SWATH-only

Precursor mass error: 2.8 ppm

(Dot: 711, Rev: 855, Total Score: 1.6)

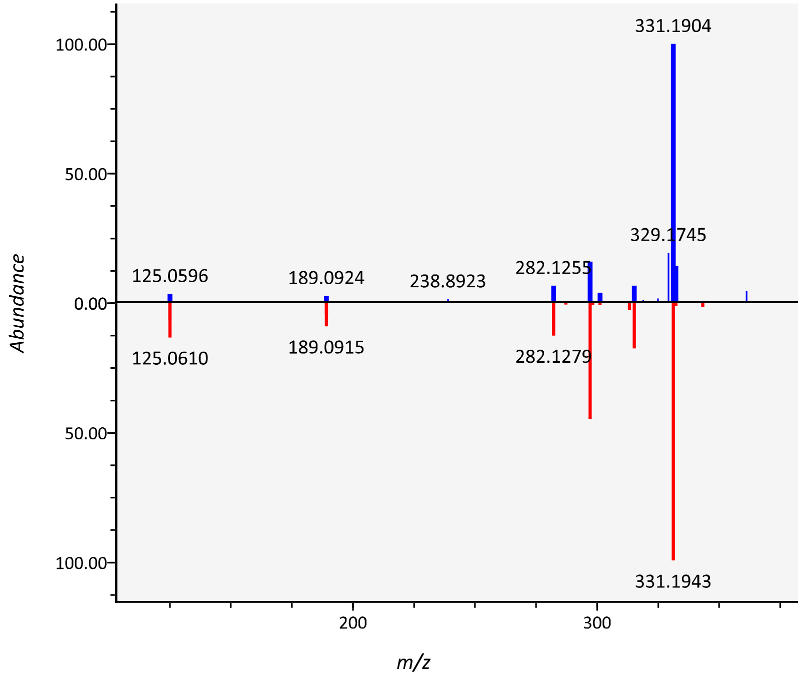

MRM+SWATH

Precursor mass error: 5 ppm

(Dot: 619, Rev: 813, Total Score: 1.5)

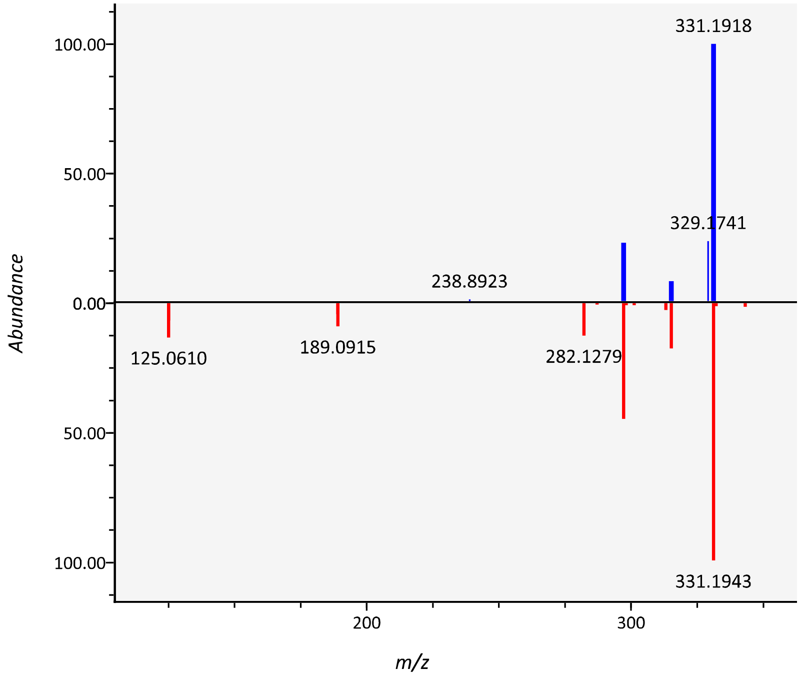

24. Indole-3-acetaldehyde (m/z 158.0611, RT 6.78 min)

<u>SWATH-only</u>

Precursor mass error: 0 ppm

(Dot: 874, Rev: 860, Total Score: 1.8)

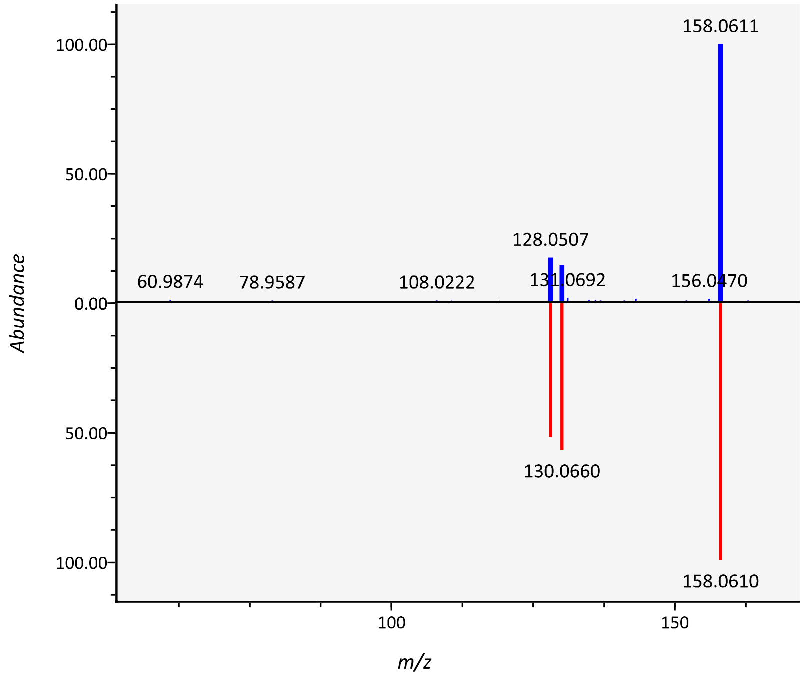

<u>MRM+SWATH</u>

Precursor mass error: 0.6 ppm

(Dot: 900, Rev: 874, Total Score: 1.8)

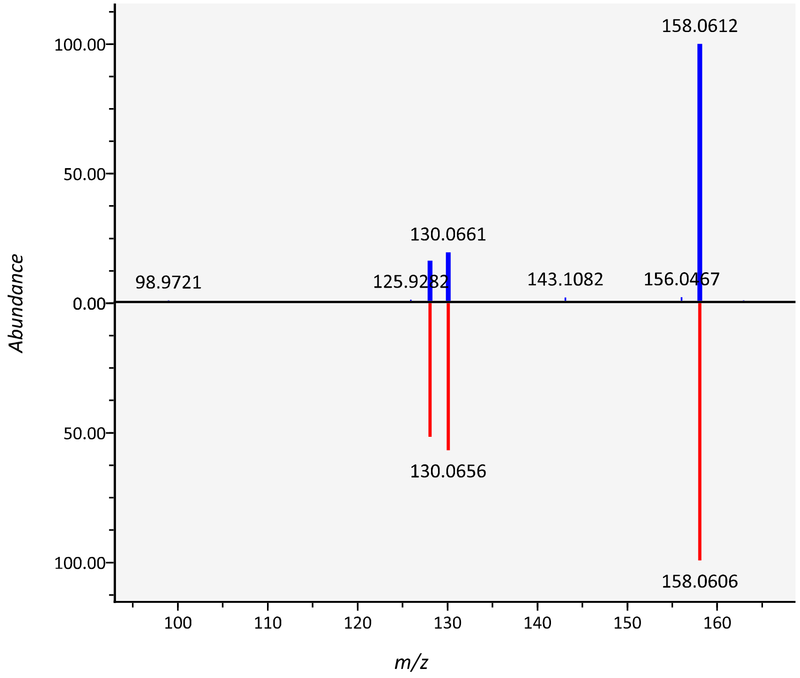

25. Hydroperoxyoctadecadienoic acid / Hpode / FA 18:2+2O (m/z 311.2228, RT 14.79 min)

SWATH-only

Precursor mass error: 5.8 ppm

(Dot: 440, Rev: 901, Total Score: 1.4)

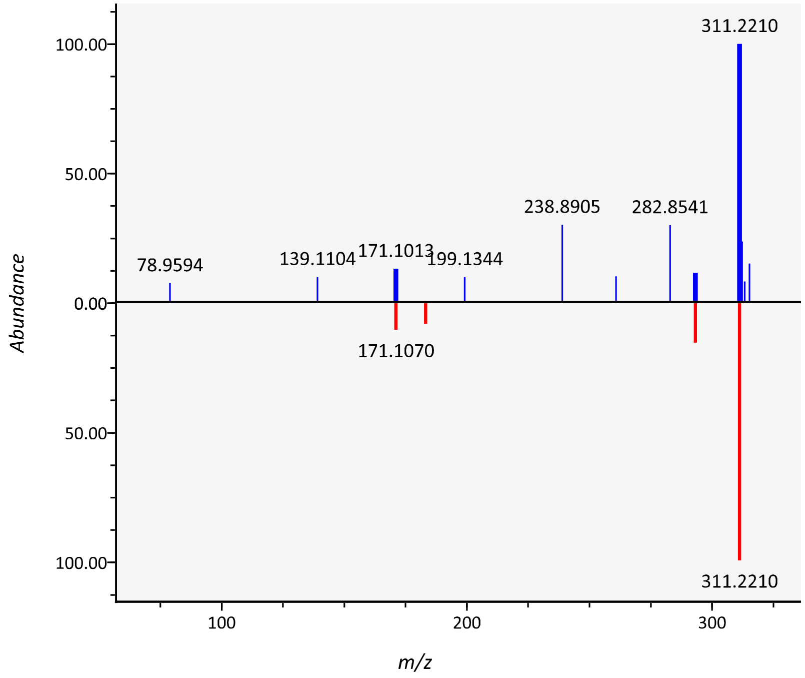

MRM+SWATH

Precursor mass error: 1.3 ppm

(Dot: 662, Rev: 794, Total Score: 1.6)

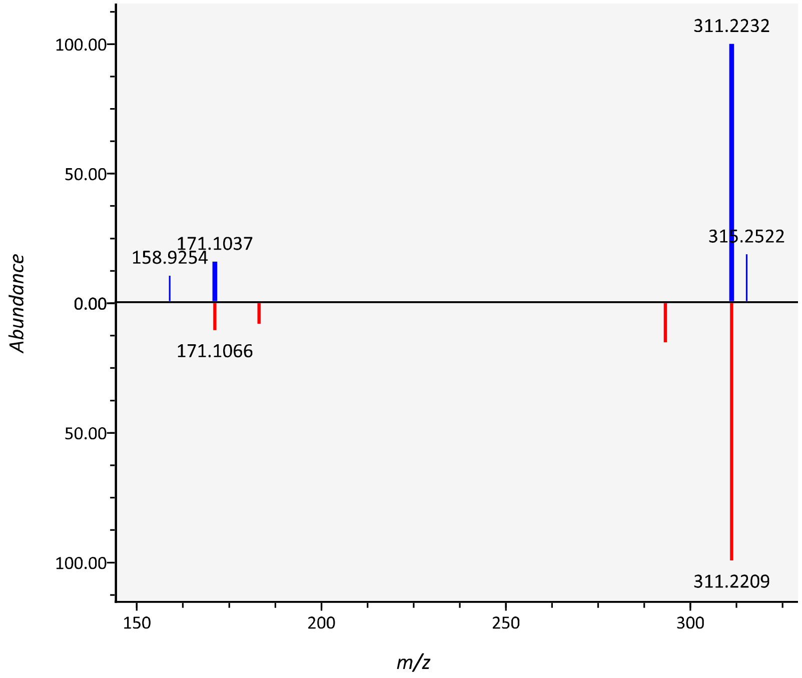

26. Lysophosphatidylethanolamine 18:1/ LPE 18:1 (m/z 478.2939, RT 16.34 min)

SWATH-only

Precursor mass error: 3.8 ppm

(Dot: 675, Rev: 766, Total Score: 1.6)

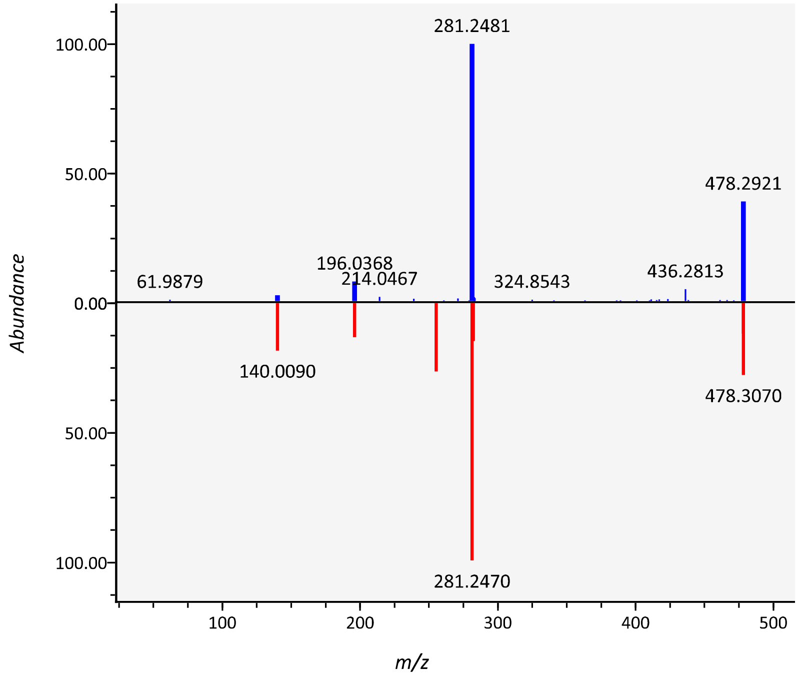

MRM+SWATH

Precursor mass error: 1.0 ppm

(Dot: 688, Rev: 770, Total Score: 1.7)

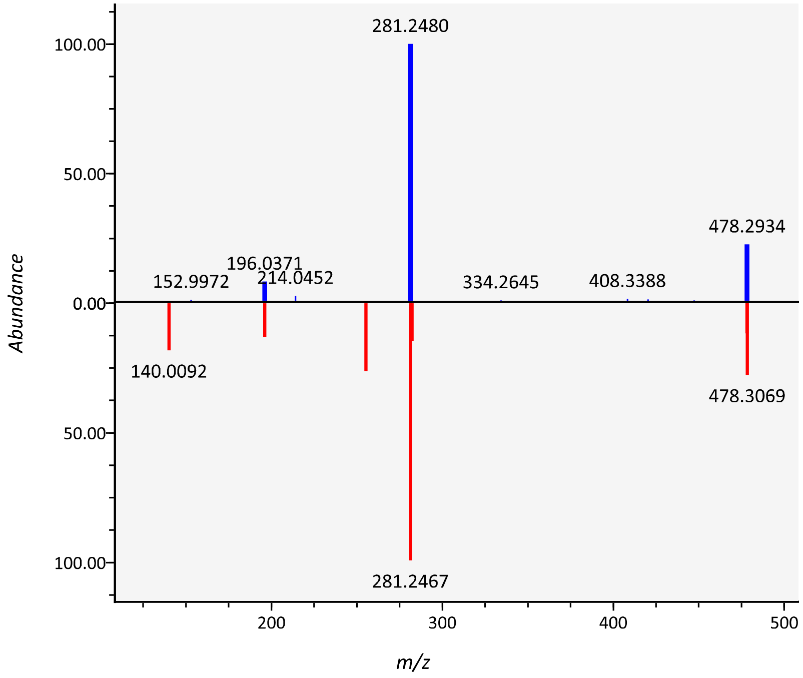

ESI+ MODE

27. Glutamic acid (*m/z* 148.0604, RT 0.58 min)

<u>SWATH-only</u>

Precursor mass error: 1.4 ppm

(Dot: 902, Rev: 900, Total Score:1.9)

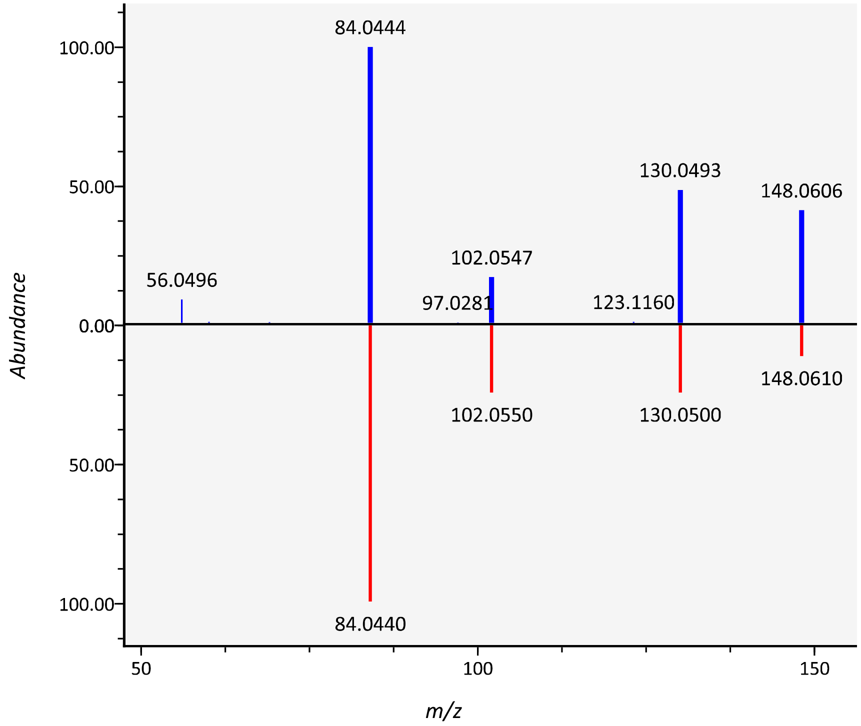

<u>MRM+SWATH</u>

Precursor mass error: 1.4 ppm

(Dot: 948, Rev: 927, Total Score: 1.9)

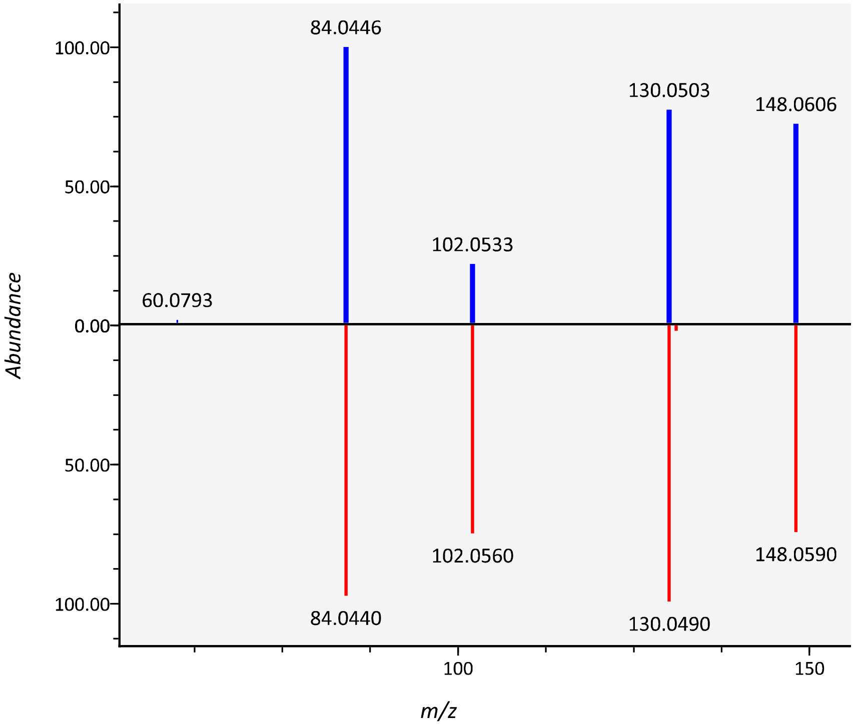

28. Acetylcarnitine (m/z 204.1230, RT 0.76 min)

<u>SWATH-only</u>

Precursor mass error: 0.5 ppm

(Dot: 537, Rev: 860, Total Score: 1.5)

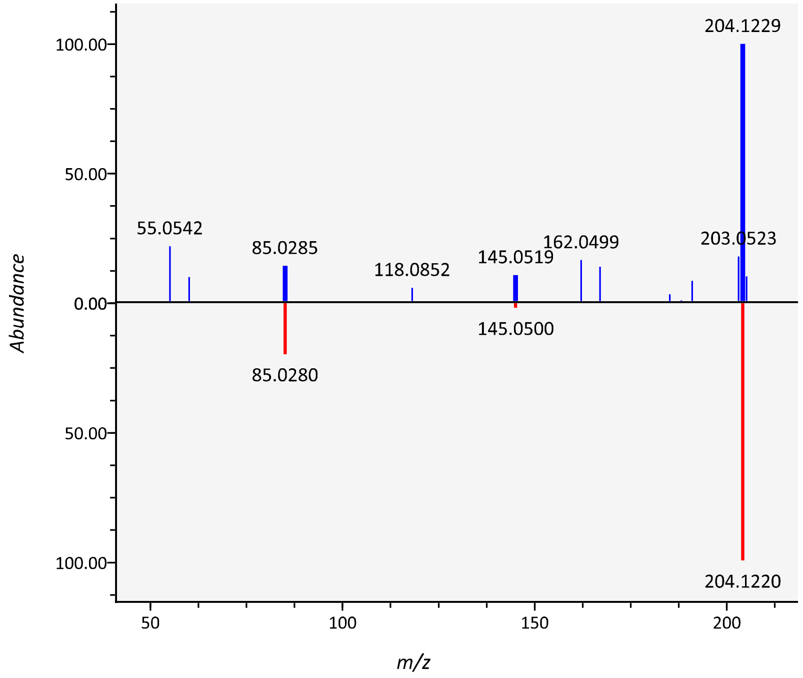

<u>MRM+SWATH</u>

Precursor mass error: 2.4 ppm

(Dot: 903, Rev: 746, Total Score: 1.7)

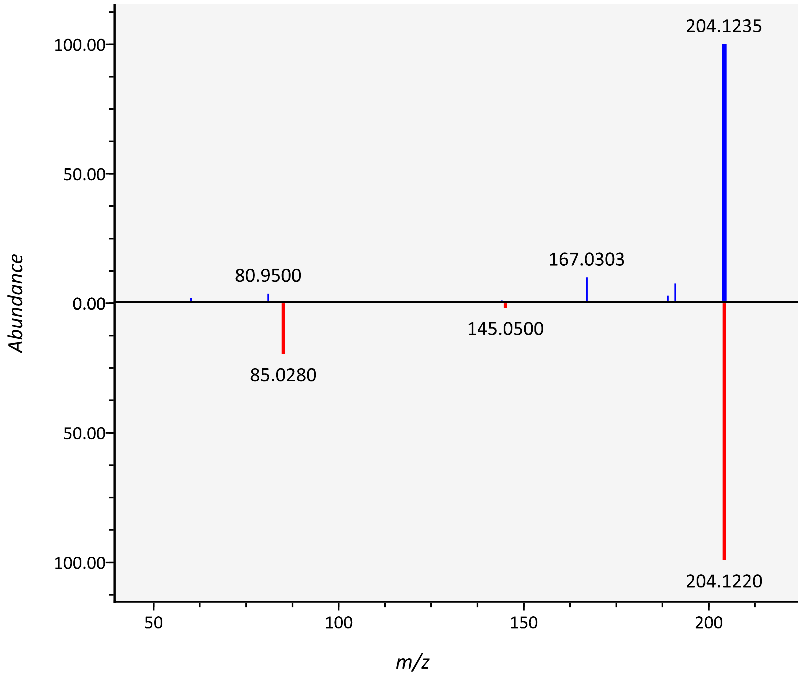

29. Methionine (m/z 150.0583, RT 0.87 min)

<u>SWATH-only</u>

Precursor mass error: 3.3 ppm

(Dot: 226, Rev: 595, Total Score: 1.1)

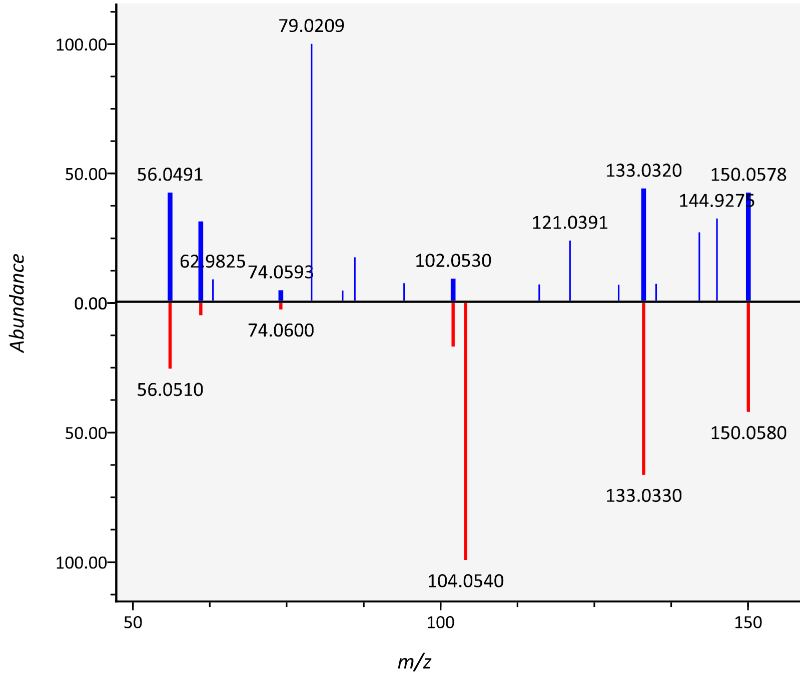

<u>MRM+SWATH</u>

Precursor mass error: 4.7 ppm

(Dot: 778, Rev: 819, Total Score: 1.7)

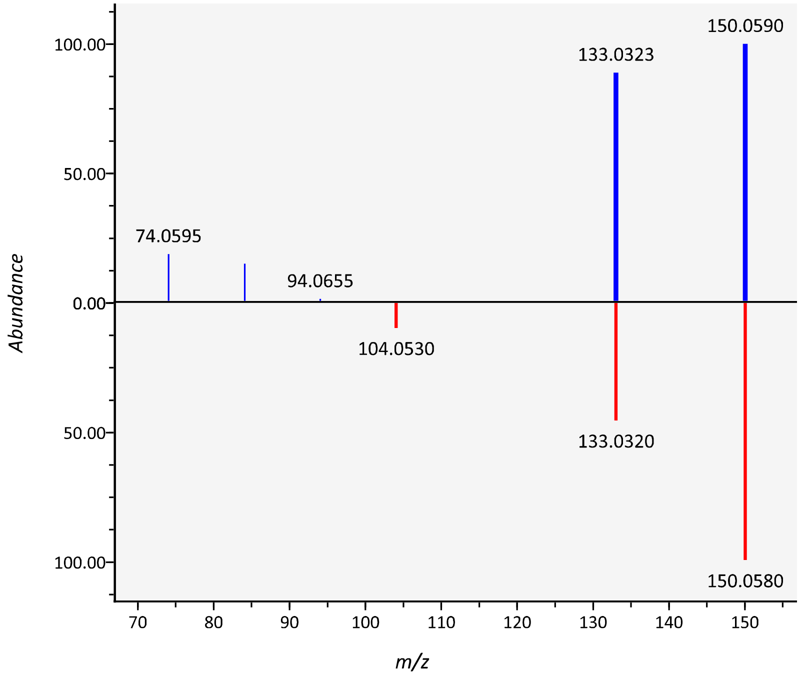

30. Tyrosine (m/z 182.0812, RT 0.92 min)

SWATH-only

Precursor mass error: 1.1 ppm

(Dot: 186, Rev: 869, Total Score: 1.1)

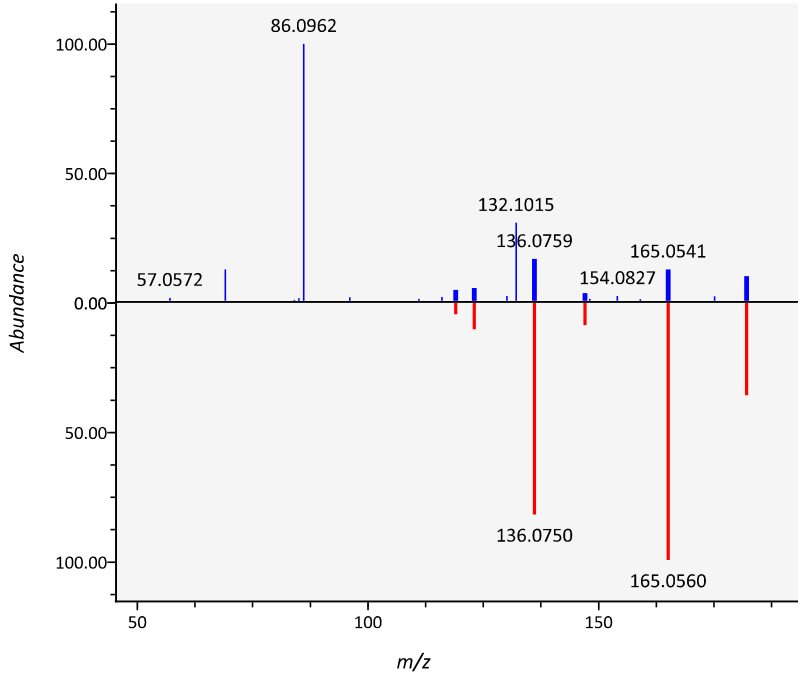

MRM+SWATH

Precursor mass error: 2.7 ppm

(Dot: 171, Rev: 795, Total Score: 1.7)

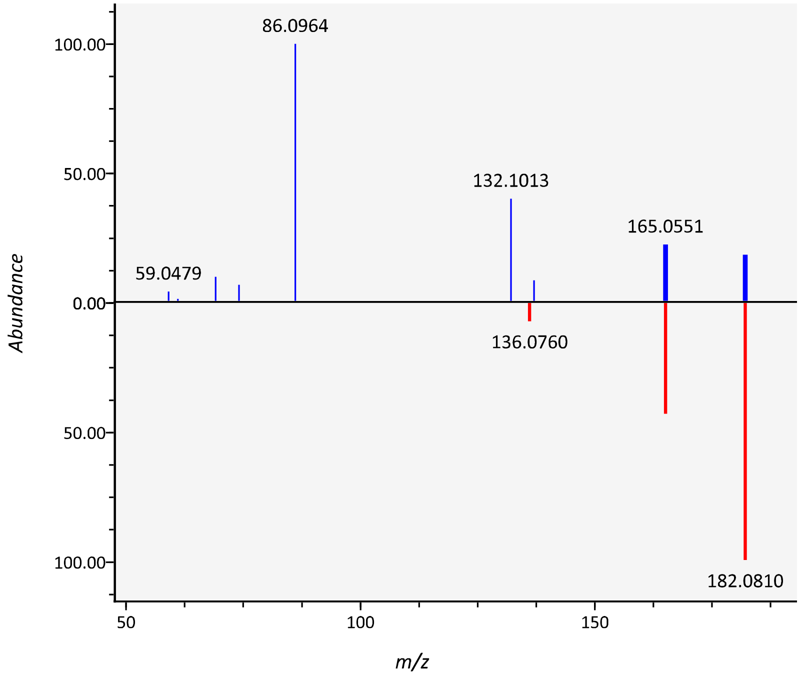

31. Isoleucine (m/z 132.1019, RT 0.92 min)

<u>SWATH-only</u>

Precursor mass error: 2.3 ppm

(Dot: 855, Rev: 874, Total Score: 1.8)

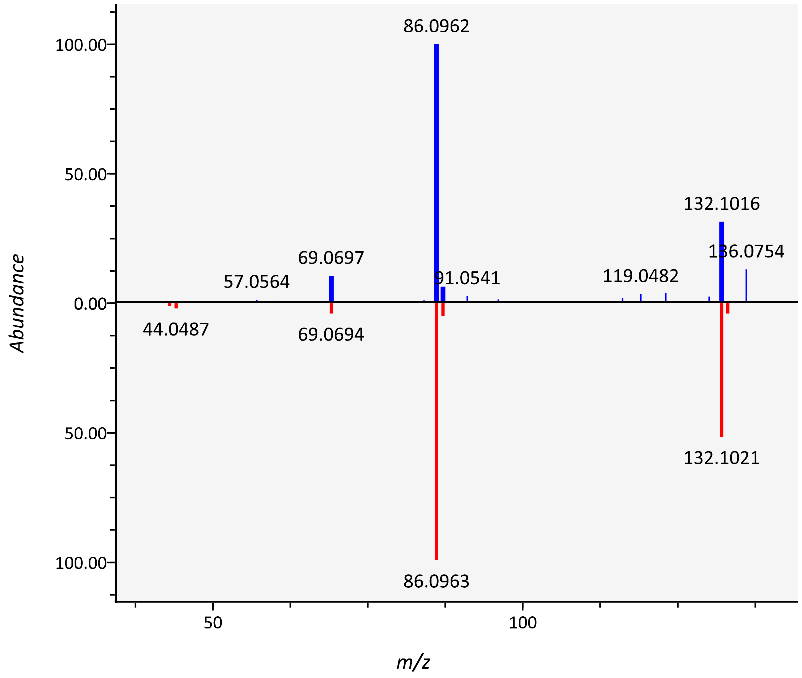

<u>MRM+SWATH</u>

Precursor mass error: 2.3 ppm

(Dot: 915, Rev: 872, Total Score: 1.8)

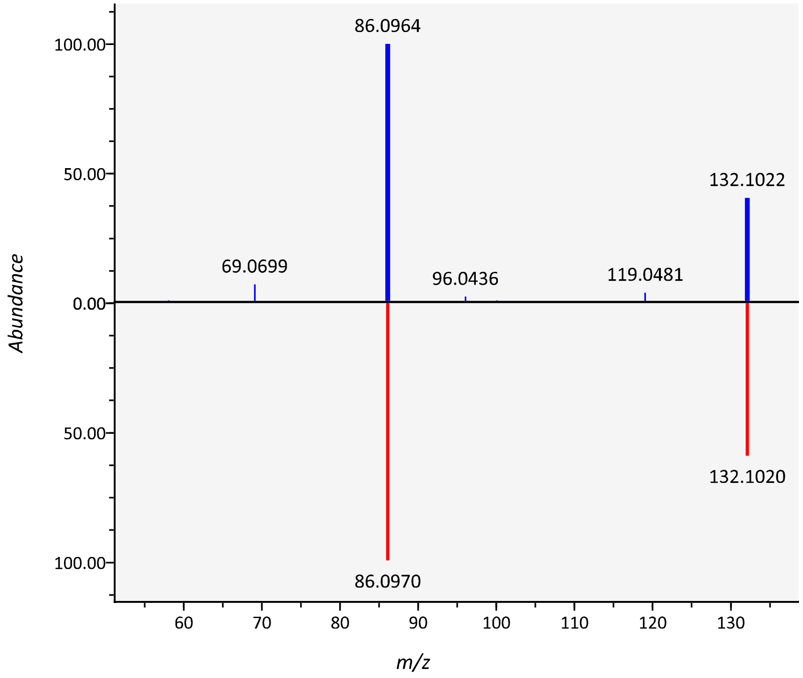

32. Leucine (m/z 132.1019, RT 1.04 min)

SWATH-only

Precursor mass error: 2.3 ppm

(Dot: 999, Rev: 880, Total Score: 1.9)

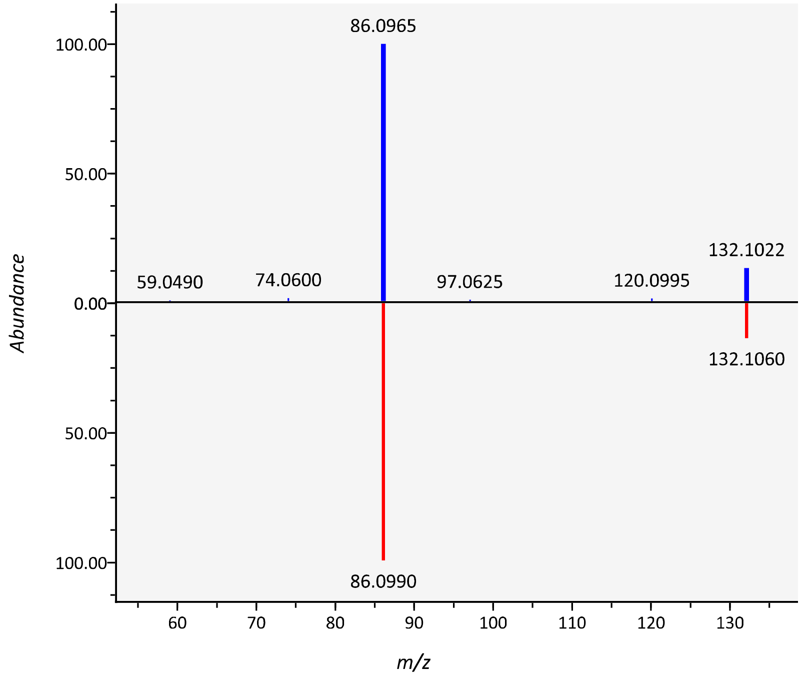

MRM+SWATH

Precursor mass error: 2.3 ppm

(Dot: 999, Rev: 880, Total Score: 1.9)

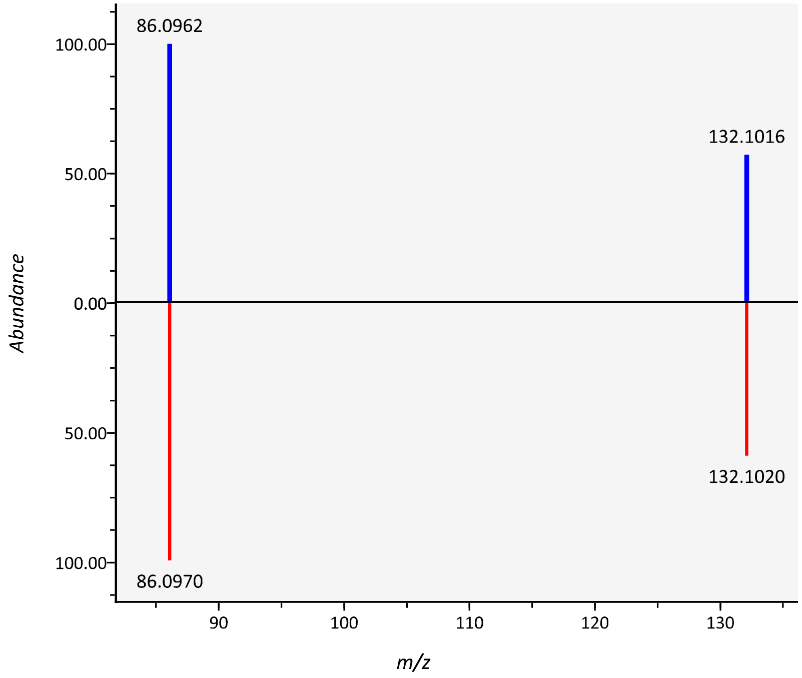

33. Phenylalanine (m/z 166.0863, RT 1.79 min)

<u>SWATH-only</u>

Precursor mass error: 1.8 ppm

(Dot: 961, Rev: 948, Total Score: 1.8)

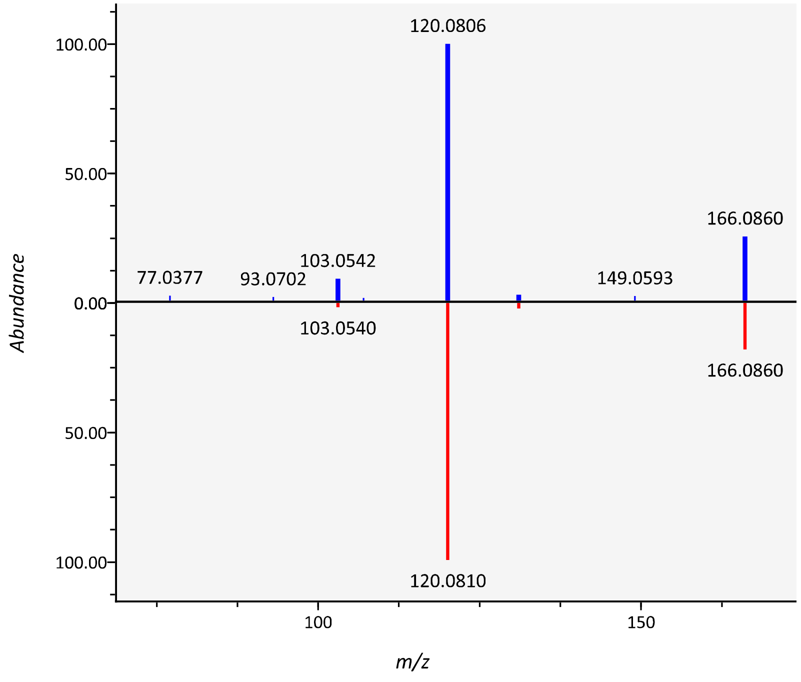

<u>MRM+SWATH</u>

Precursor mass error: 0.6 ppm

(Dot: 972, Rev: 967, Total Score: 1.9)

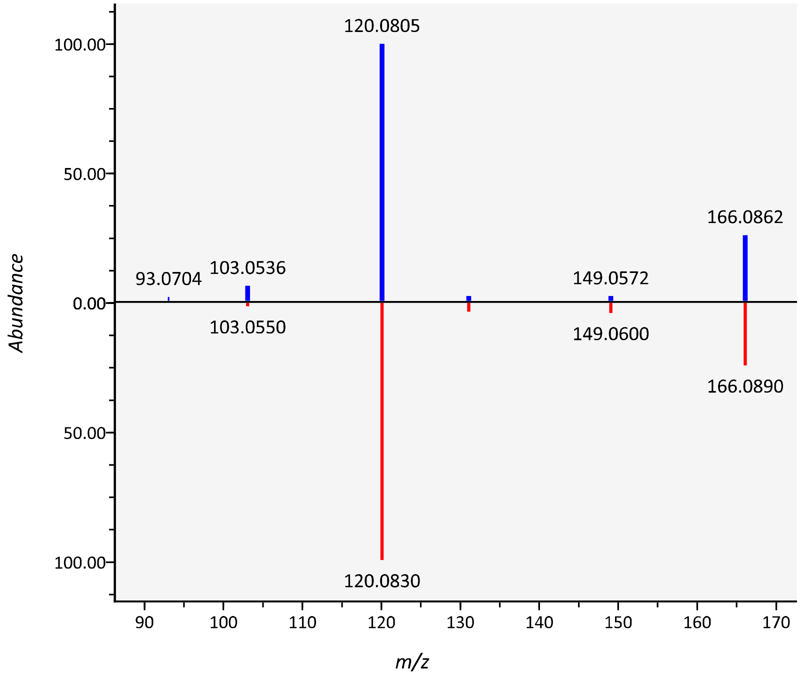

34. Indoline (m/z 120.0808, RT 1.79 min)

SWATH-only

Precursor mass error: 1.7 ppm

(Dot: 976, Rev: 735, Total Score: 1.8)

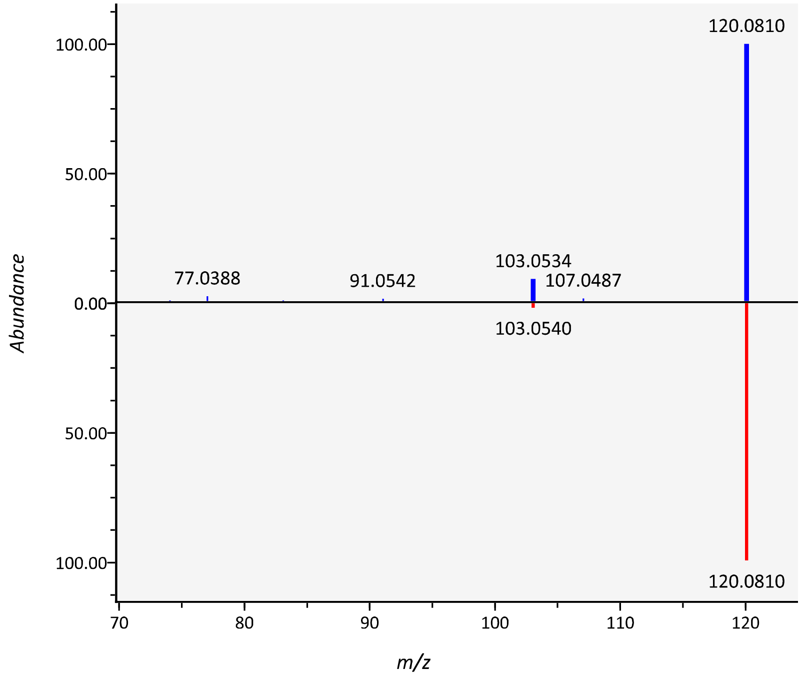

MRM+SWATH

Precursor mass error: 2.5 ppm

(Dot: 974, Rev: 742 Total Score: 1.9)

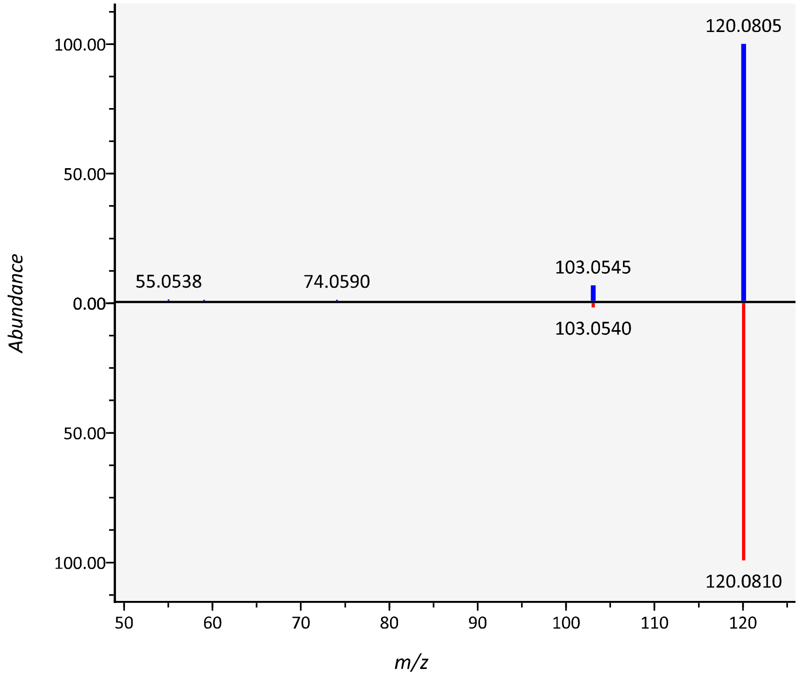

35. Theobromine (m/z 181.0720, RT 2.99 min)

<u>SWATH-only</u>

Precursor mass error: 0 ppm

(Dot: 896, Rev: 865, Total Score: 1.8)

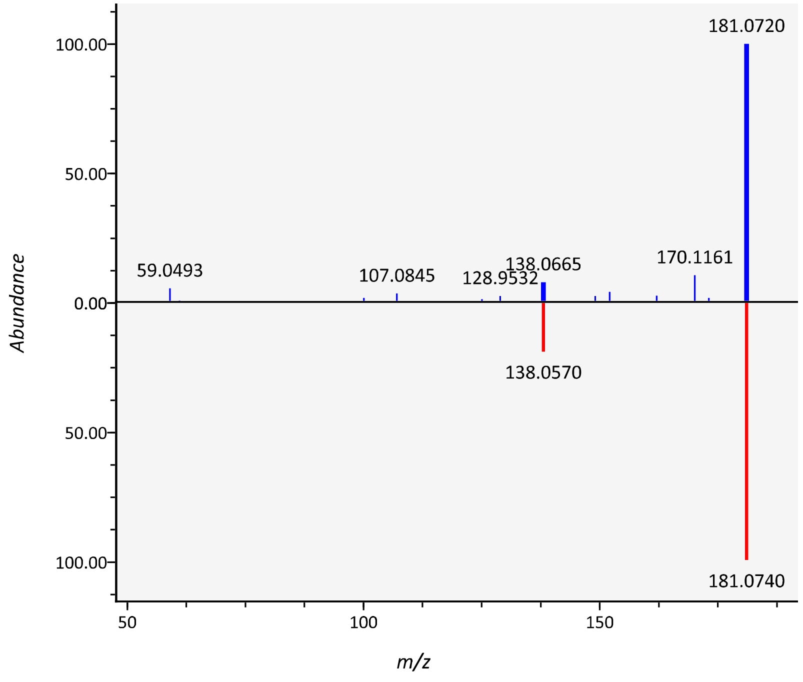

<u>MRM+SWATH</u>

Precursor mass error: 2.2 ppm

(Dot: 822, Rev: 863 Total Score: 1.7)

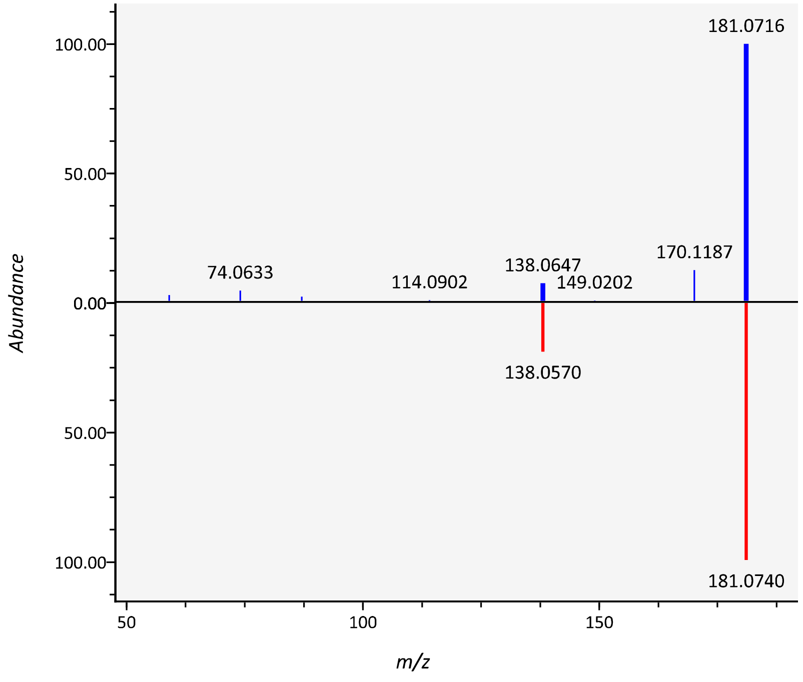

36. Tryptophan (m/z 205.0972, RT 3.04 min)

<u>SWATH-only</u>

Precursor mass error: 5.4 ppm

(Dot: 894, Rev: 835, Total Score: 1.8)

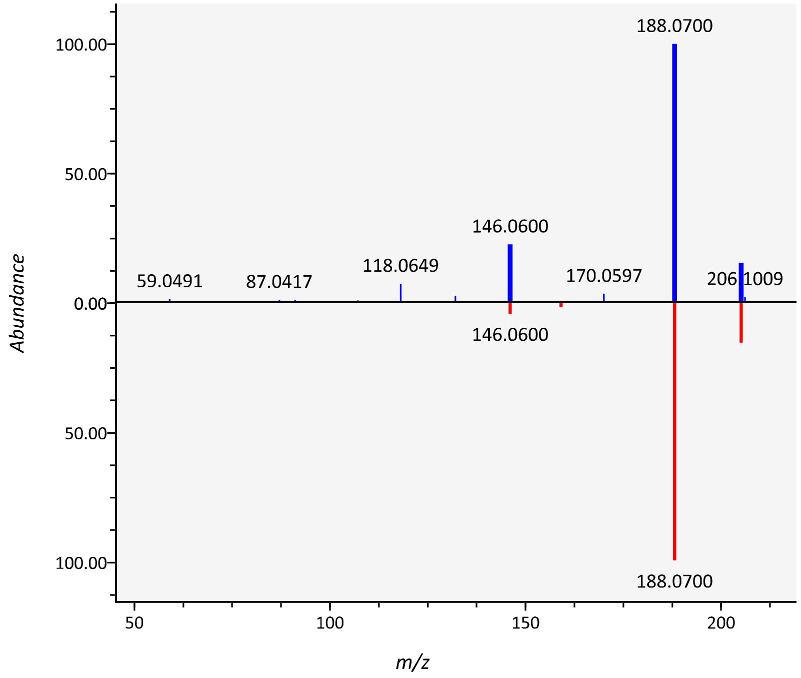

<u>MRM+SWATH</u>

Precursor mass error: 2.9 ppm

(Dot: 822, Rev: 863 Total Score: 1.7)

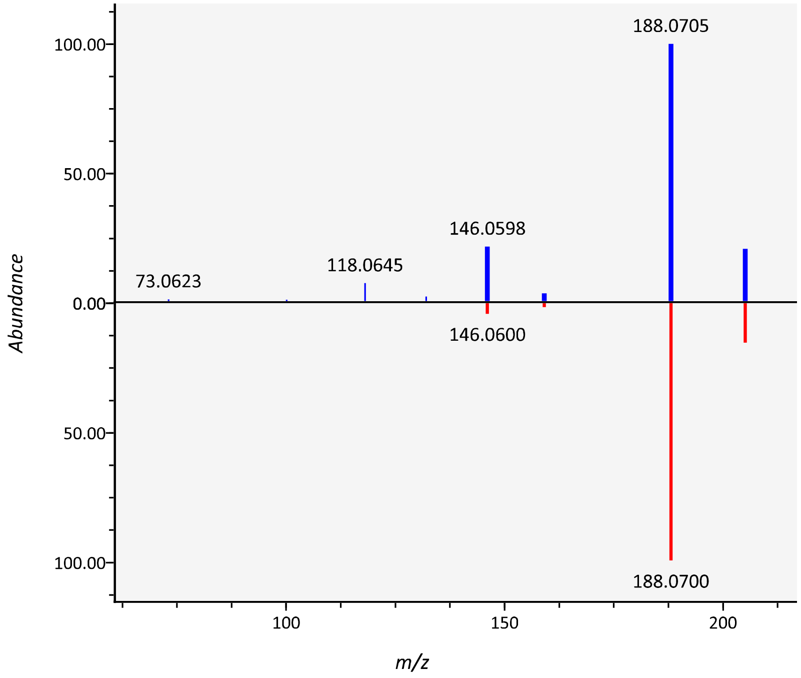

37. 3-Formylindole (m/z 146.0600, RT 3.05 min)

SWATH-only

Precursor mass error: 0 ppm

(Dot: 883, Rev: 845, Total Score: 1.8)

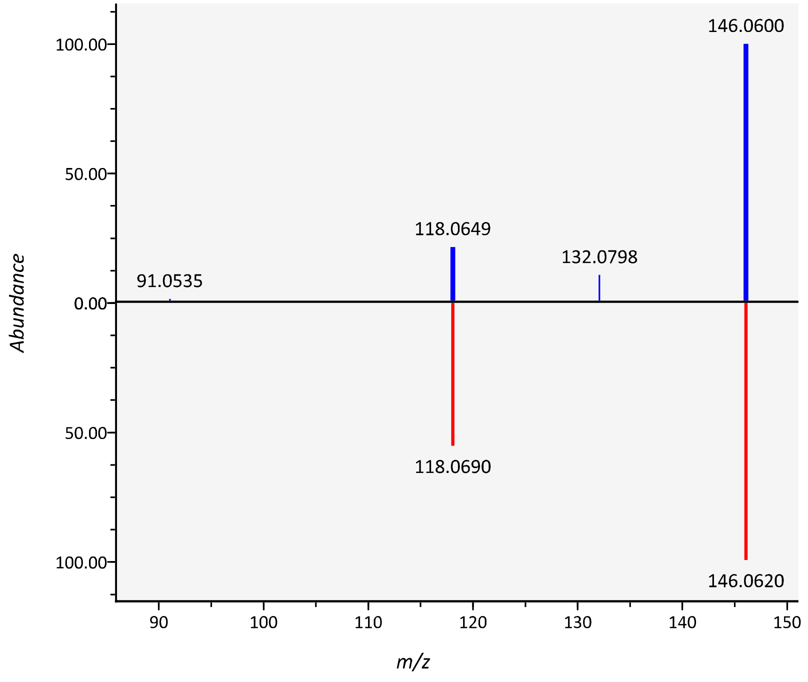

MRM+SWATH

Precursor mass error: 3.4 ppm

(Dot: 969, Rev: 856 Total Score: 1.9)

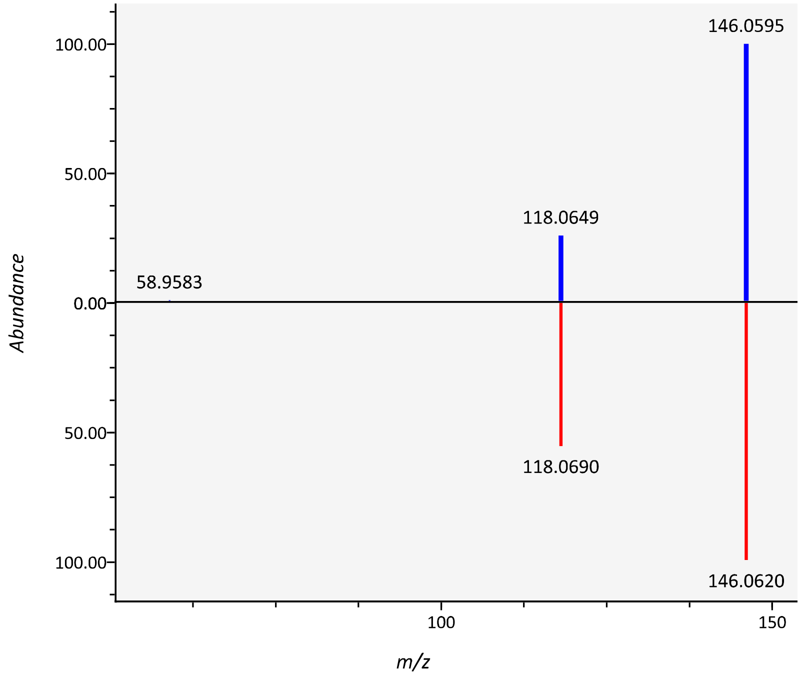

38. Acetaminophen / Paracetamol (m/z 152.0706, RT 3.10 min)

SWATH-only

Precursor mass error: 0.7 ppm

(Dot: 969, Rev: 856, Total Score: 1.8)

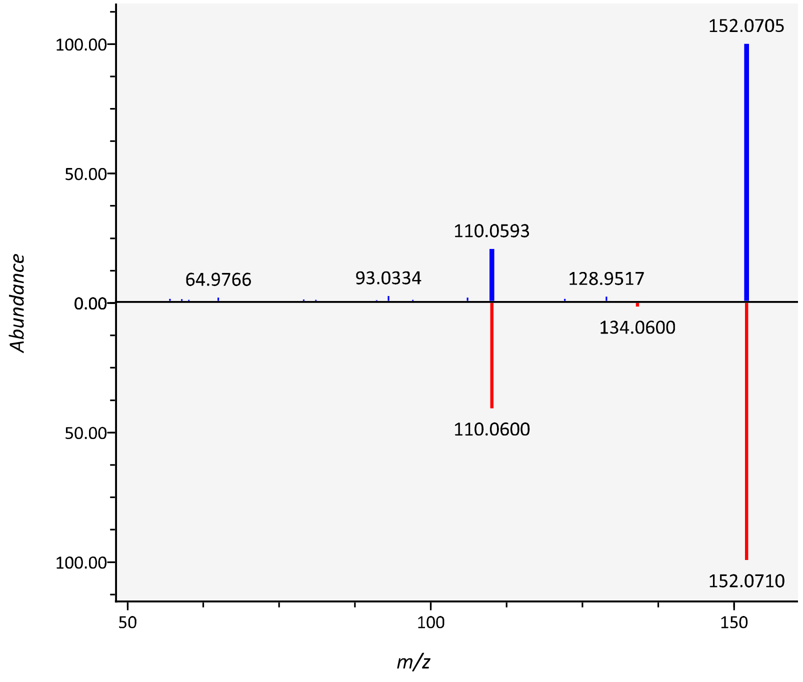

MRM+SWATH

Precursor mass error: 2.0 ppm

(Dot: 827, Rev: 728 Total Score: 1.7)

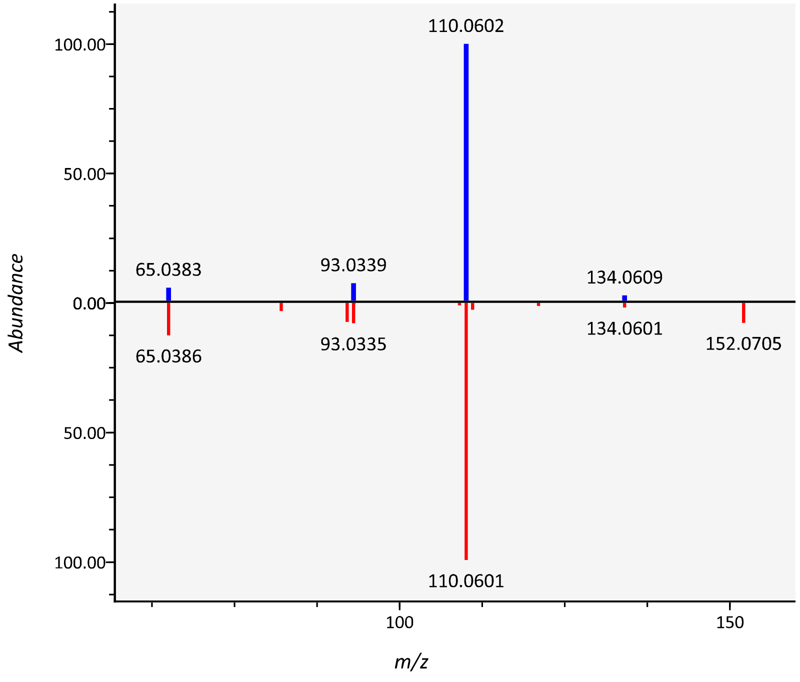

39. Theophylline (m/z 181.0720, RT 3.25 min)

<u>SWATH-only</u>

Precursor mass error: 5.0 ppm

(Dot: 971, Rev: 861, Total Score: 1.9)

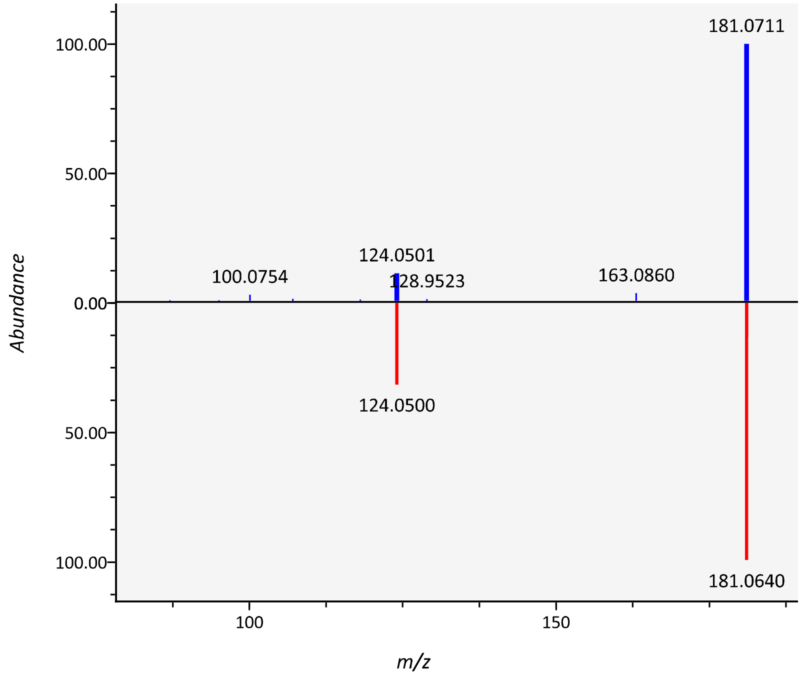

<u>MRM+SWATH</u>

Precursor mass error: 2.2 ppm

(Dot: 919, Rev: 859 Total Score: 1.8)

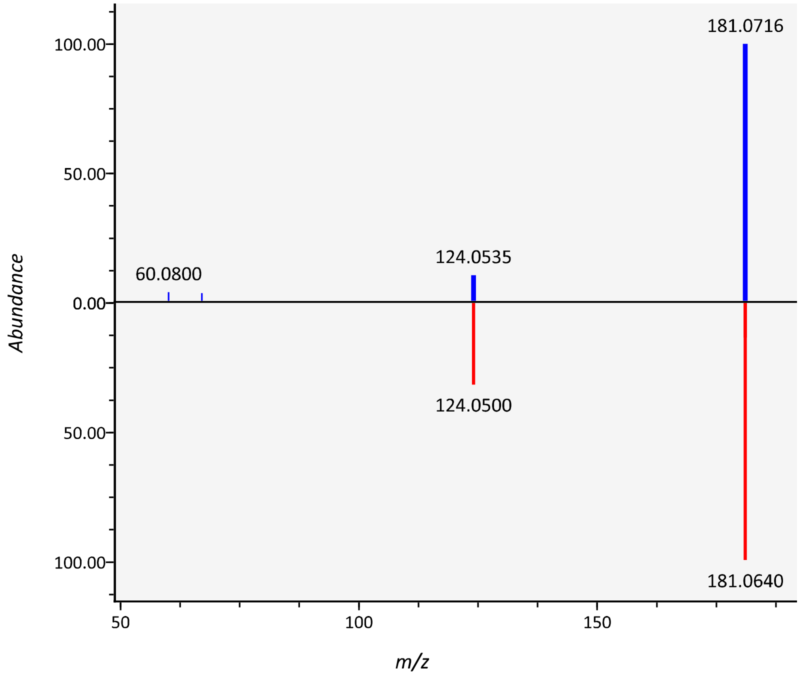

40. Indole-3-acetamide (m/z 175.0866, RT 3.49 min)

SWATH-only

Precursor mass error: 4.0 ppm

(Dot: 513, Rev: 595, Total Score: 1.4)

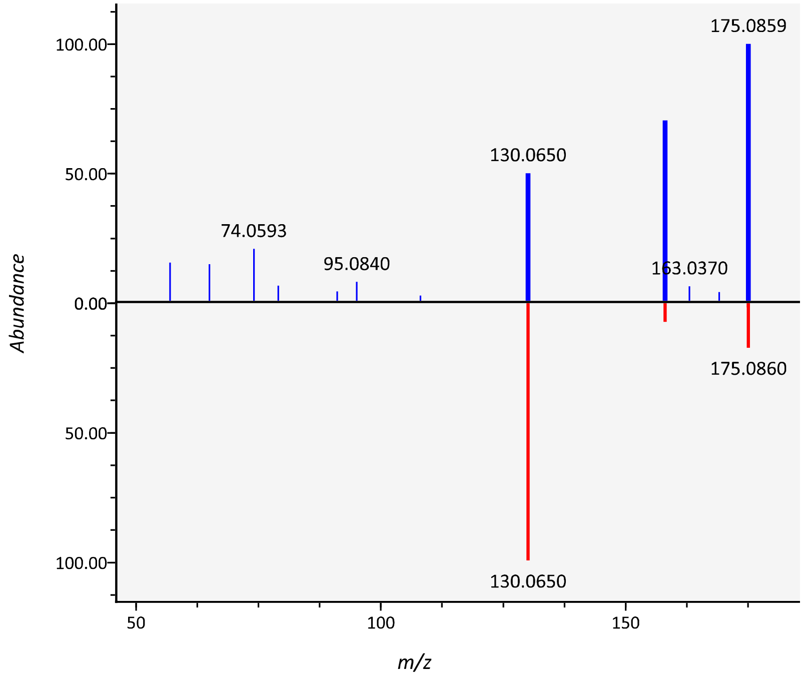

MRM+SWATH

Precursor mass error: 1.1 ppm

(Dot: 371, Rev: 419 Total Score: 1.2)

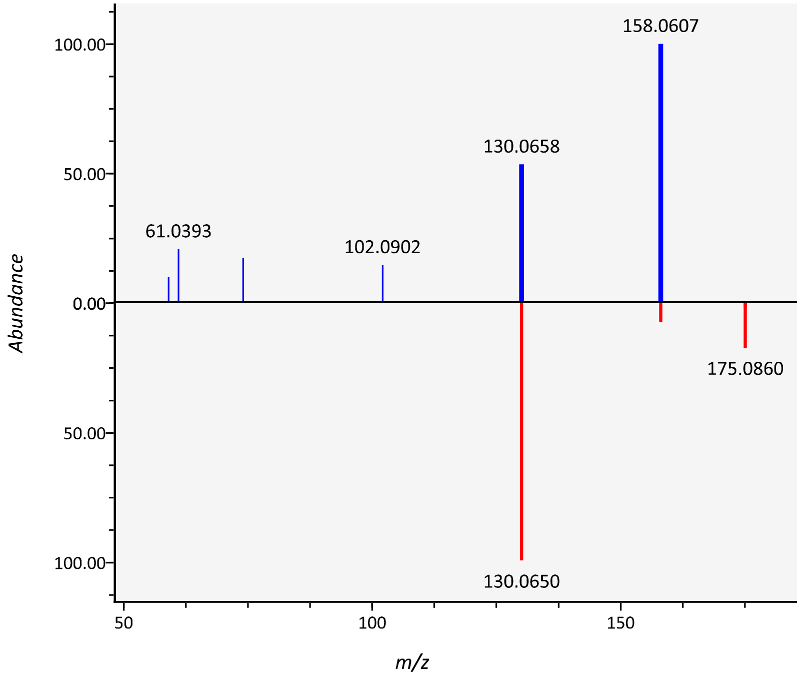

41. Glycoursodeoxycholic acid (m/z 450.3214, RT 6.88 min)

SWATH-only

Precursor mass error: 1.5 ppm

(Dot: 951, Rev: 967, Total Score: 1.9)

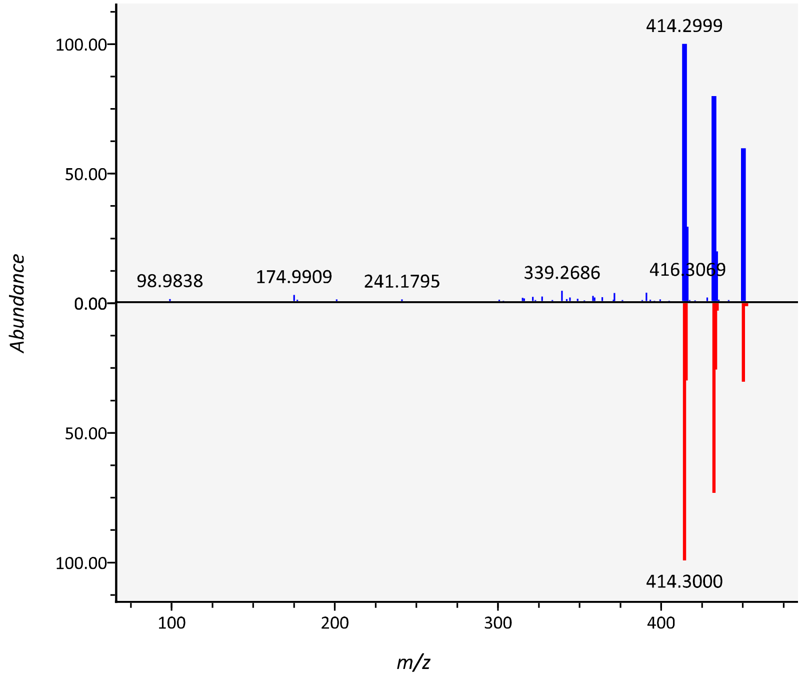

MRM+SWATH

Precursor mass error: 2.4 ppm

(Dot: 756, Rev: 862 Total Score: 1.7)

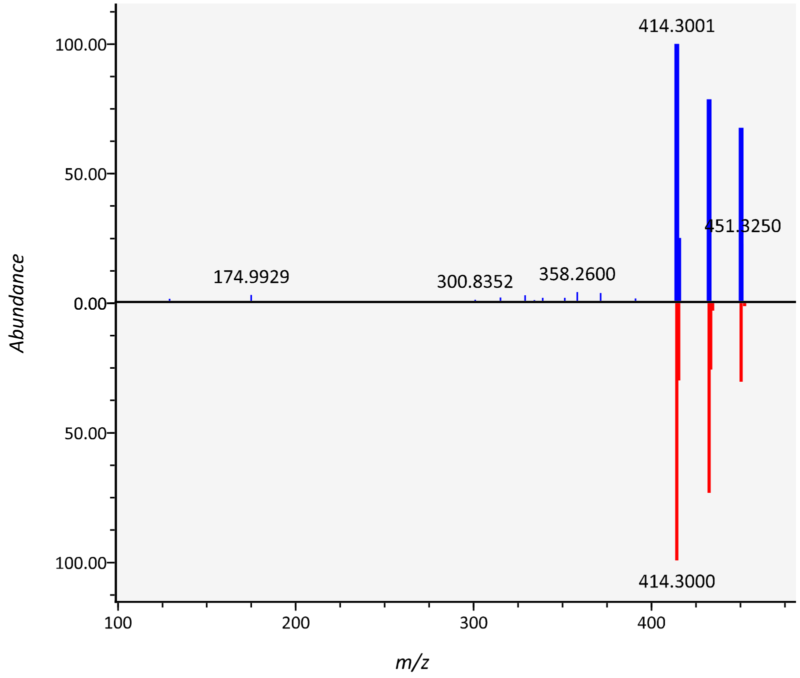

42. 1-pentadecanoyl-2-hydroxy-sn-glycero-3-phosphocholine (m/z 482.3241, RT 15.4 min)

SWATH-only

Precursor mass error: 0.6 ppm

(Dot: 433, Rev: 814, Total Score: 1.3)

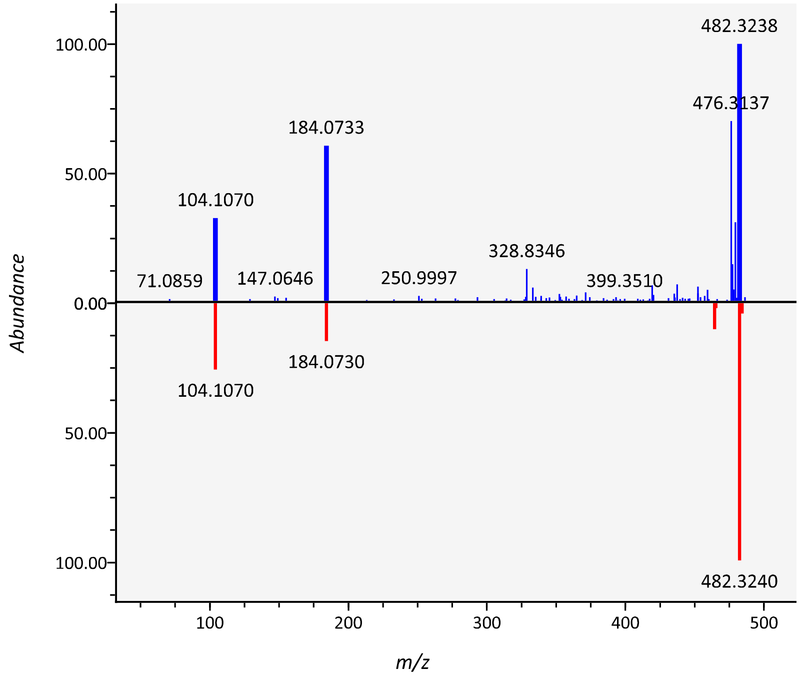

MRM+SWATH

Precursor mass error: 2.1 ppm

(Dot: 390, Rev: 796 Total Score: 1.2)

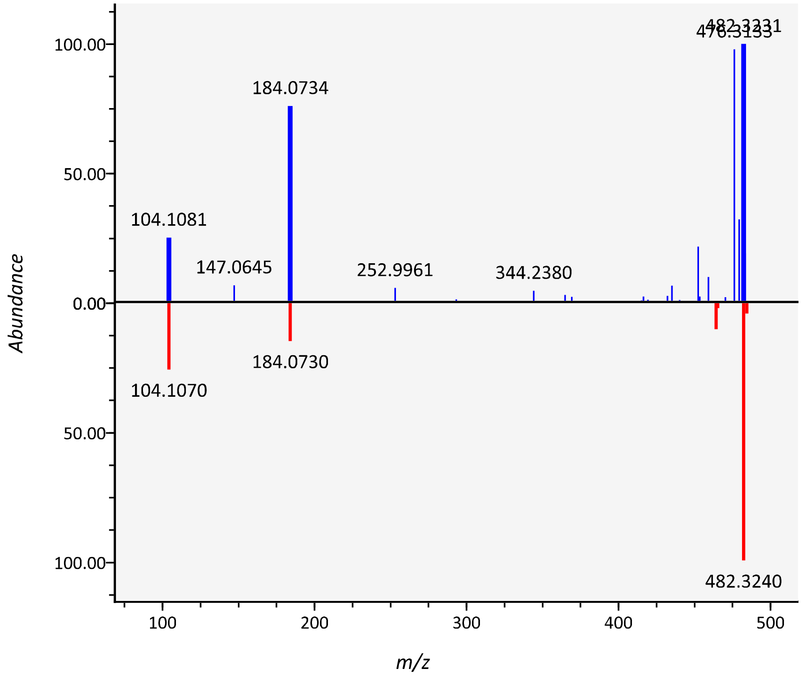

43. 1-palmitoyl-2-hydroxy-sn-glycero-3-phosphoethanolamine (m/z 454.2928, RT 16.1 min)

<u>SWATH-only</u>

Precursor mass error: 1.3 ppm

(Dot: 797, Rev: 832, Total Score: 1.6)

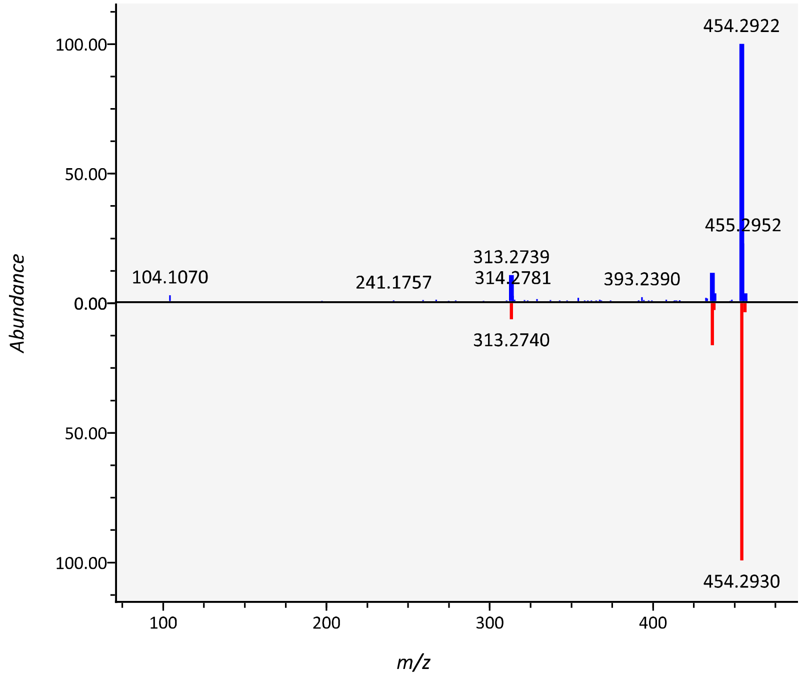

<u>MRM+SWATH</u>

Precursor mass error: 3.1 ppm

(Dot: 898, Rev: 850 Total Score: 1.8)

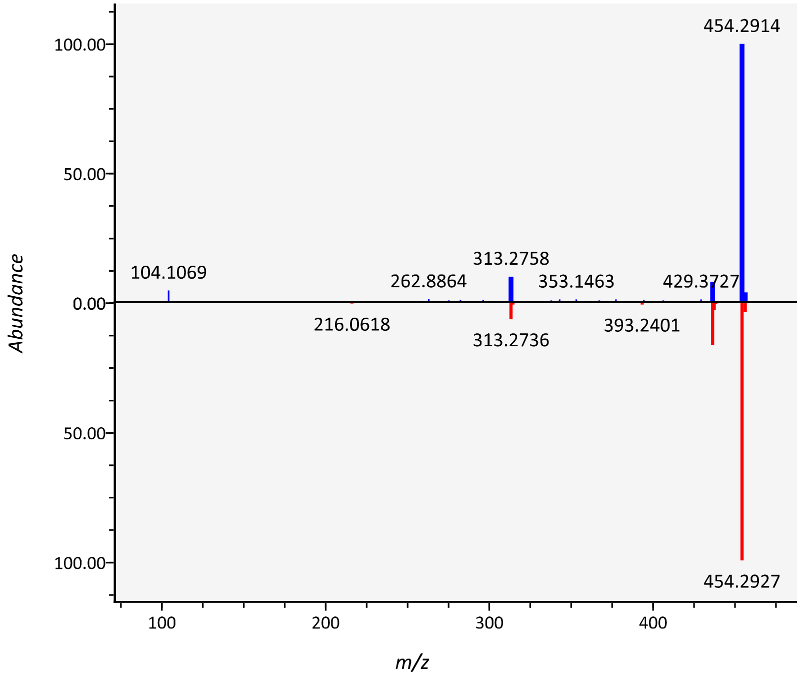

44. Di-n-butyl phthalate (m/z 279.1591, RT 17.01 min) - ID confirmed with standard (Level 1)

<u>SWATH-only</u>

Precursor mass error: 5.0 ppm

(Dot: 988, Rev: 875, Total Score: 1.8)

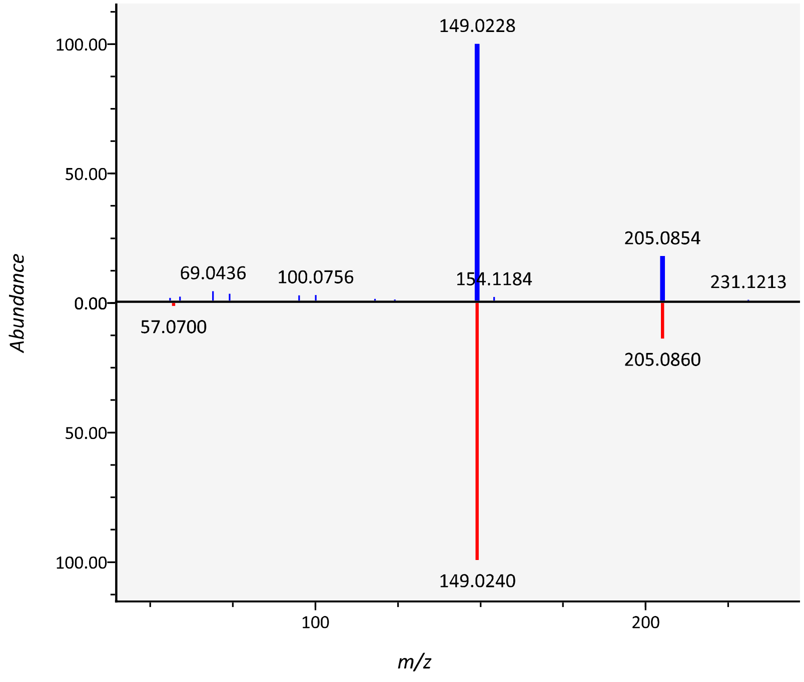

<u>MRM+SWATH</u>

Precursor mass error: 3.2 ppm

(Dot: 982, Rev: 868 Total Score: 1.8)

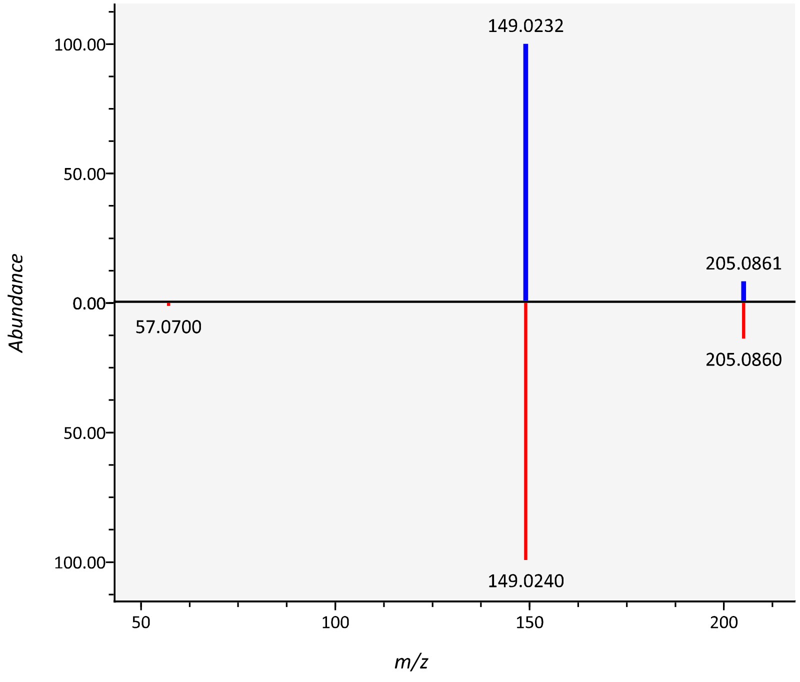

45. Bis(2-ethylhexyl) phthalate (m/z 391.2843, RT 17.02 min)

<u>SWATH-only</u>

Precursor mass error: 1.8 ppm

(Dot: 679, Rev: 729, Total Score: 1.6)

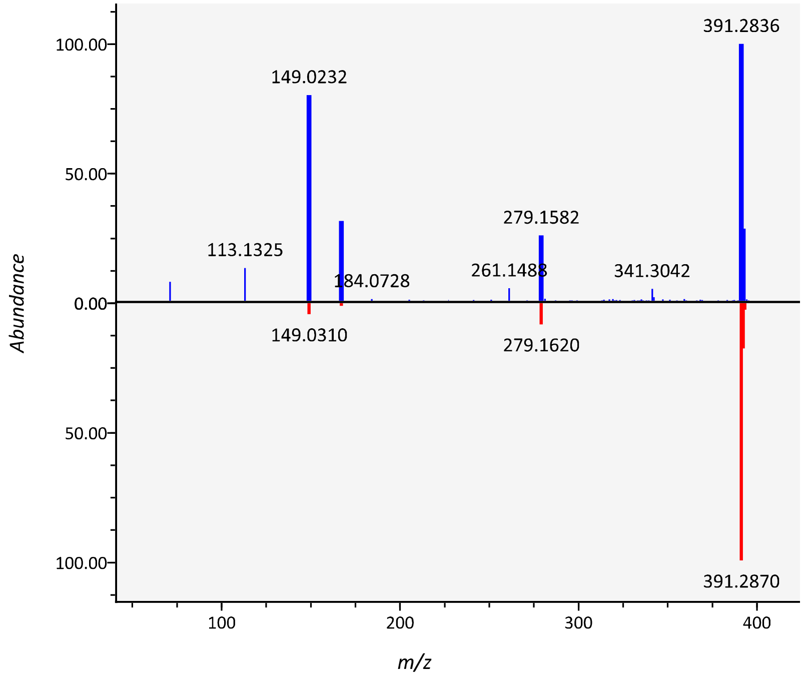

<u>MRM+SWATH</u>

Precursor mass error: 1.8 ppm

(Dot: 692, Rev: 743 Total Score: 1.6)

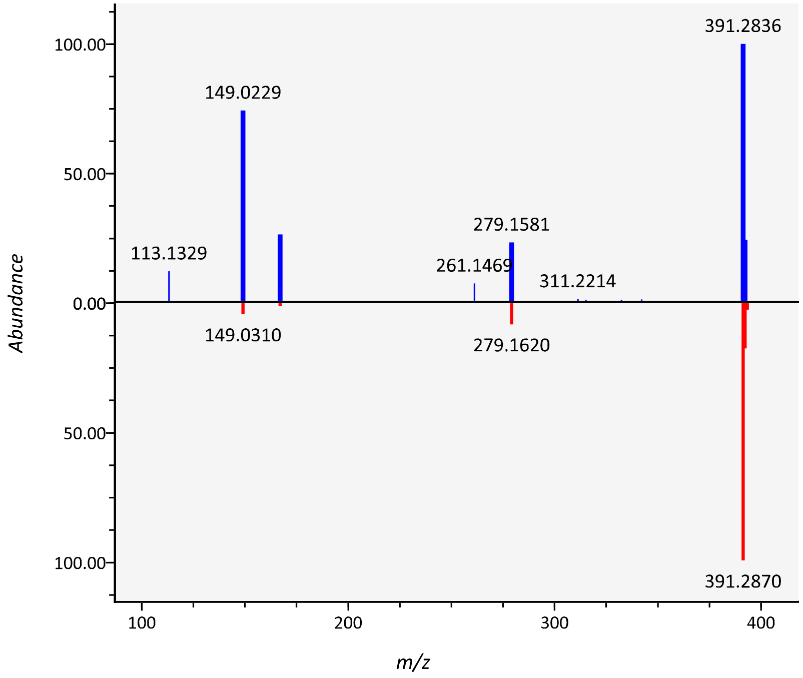

Supplement: Supplementary file 2 — ac4c01630_si_002.pdf [file ac4c01630_si_002.pdf]
